# Skeleton-Forming Responses of Reef-Building Corals under Ocean Acidification

**DOI:** 10.34133/research.0736

**Published:** 2025-06-11

**Authors:** Yixin Li, Hongwei Zhao, Yunpeng Zhao, Xin Liao, J.-Y. Chen, Yanping Qin, Zuhong Lu, Yuehuan Zhang, Chunpeng He

**Affiliations:** ^1^ State Key Laboratory of Coastal and Offshore Engineering, Dalian University of Technology, Dalian 116024, China.; ^2^ State Key Laboratory of Bioelectronics, School of Biological Science and Medical Engineering, Southeast University, Nanjing 210096, China.; ^3^ State Key Laboratory of Marine Resources Utilization in South China Sea, Hainan University, Haikou 570228, China.; ^4^ Guangxi Key Lab of Mangrove Conservation and Utilization, Guangxi Mangrove Research Center, Guangxi Academy of Sciences, Beihai 536000, China.; ^5^ Nanjing Institute of Geology and Palaeontology, Chinese Academy of Sciences, Nanjing 210008, China.; ^6^ Southern Marine Science and Engineering Guangdong Laboratory (Guangzhou), Guangzhou 511458, China.; ^7^ South China Sea Institute of Oceanology, Chinese Academy of Science, Guangzhou 510301, China.

## Abstract

Ocean acidification is becoming more prevalent and may contribute to coral reef degradation, yet our understanding of its role in global reef decline remains limited. Therefore, there is an urgent need to study the impact of reduced pH levels on the growth patterns of major reef-building corals. Here, we studied the skeleton-forming strategies of 4 widely distributed coral species in a simulated acidified habitat with a pH of 7.6 to 7.8. We reconstructed and visualized the skeleton-forming process, quantified elemental calcium loss, and determined gene expression changes. The results suggest that different reef-building corals have diverse growing strategies in lower pH conditions. A unique “cavity-like” forming process starts from the inside of the skeletons of *Acropora muricata*, which sacrifices skeletal density to protect its polyp–canal system. The forming patterns in *Pocillopora damicornis*, *Montipora capricornis*, and *Montipora foliosa* were characterized by “osteoporosis”, exhibiting disordered skeletal structures, insufficient synthesis of adhesion proteins, and low bone mass, correspondingly. In addition, we found that damage from acidification particularly affects pre-existing skeletal structures in the colony. These results enhance our understanding of skeleton-forming strategies in major coral species under lower pH conditions, providing a foundation for coral reef protection and restoration amidst increasing ocean acidification.

## Introduction

Scleractinian reef-building corals first appeared on Earth approximately 250 million years ago and have experienced unprecedented and rapid changes in oceanic pH over the last 2 centuries [1]. Anthropogenic carbon dioxide (CO_2_) emissions have lowered ocean pH by an average of 0.1 units since the Industrial Revolution (~200 years ago), with even greater changes occurring in localized areas subjected to heavy pollution [2]. Over the past 20 million years, oceanic pH has varied by less than ± 0.3 units [3,4]. According to forecasts from the Intergovernmental Panel on Climate Change (IPCC), the global average seawater pH is expected to decline to 7.8 to 7.9 by 2,100 (current pH is 8.1 ± 0.1), potentially devastating organisms with calcium carbonate skeletons, such as corals [5].

Recent research suggests that the acidification of global oceans leads to a reduction in the diversity, biomass, and trophic complexity of benthic marine communities, resulting in substantial negative impacts on certain marine species with potential ecosystem-level consequences, posing a threat to the biodiversity and ecosystem function of coral reef ecosystems [6–10]. It is anticipated that the net calcification of reef-building corals will decline as seawater pH decreases, potentially resulting in net dissolution by the end of this century [11,12]. Furthermore, habitats with lower pH can disrupt calcification efficiency, coral–Symbiodiniaceae holobiont functioning, self-healing ability, and larval attachment due to lack of natural resistance mechanisms in some reef-building corals [13–17]. However, evidence suggests that reef-building corals may possess inherent resilience against extinction events caused by ocean acidification [18]. This resilience is inferred from past large-scale extinctions that occurred alongside severe ocean acidification [19].

Indeed, previous investigations employing morphological assessments have documented structural changes in corals exposed to acidified seawater [20,21]. Studies using techniques such as traditional skeletal analysis and scanning electron microscopy (SEM) have revealed impacts including reduced skeletal density, increased porosity, and alterations to skeletal ultrastructure and crystallography under low-pH conditions [22–24]. For instance, decreased bulk skeletal density has been observed in several coral species under simulated future pH levels [23], while modifications to the arrangement and morphology of aragonite crystals within the skeleton have been reported through ultrastructural analysis [24]. While these studies provided valuable insights into the detrimental effects of acidification on coral skeletons, many focused on endpoint measurements, bulk density changes, or surface ultrastructure. A deeper understanding of the process of skeletal alteration, particularly involving the complex internal architecture and the vital polyp–canal system that integrates colony-wide responses, necessitates high-resolution, time-series, and 3-dimensional (3D) analysis. Therefore, adopting advanced morphological techniques like micro-computed tomography (micro-CT) allows for non-destructive, detailed 3D visualization and quantification of volumetric changes and structural reorganization within the coral colony over time. Complementing this with SEM coupled with energy dispersive spectrometry (SEM-EDS) enables fine-scale examination of ultrastructural integrity and associated shifts in elemental composition at specific growth fronts. This detailed morphological approach is crucial for elucidating the mechanisms underlying coral responses to acidification, moving beyond bulk changes to understand how and where skeletal accretion or dissolution occurs within the complex colony structure.

In this study, we investigated the impact of an acidified environment on the growth process of reef-building corals by examining the growth and mineralization responses of 4 widely distributed coral species (*Acropora muricata*, *Montipora capricornis*, *Montipora foliosa*, and *Pocillopora damicornis*) under reduced pH levels. These species were selected due to their widespread distribution and varying skeletal architectures, which may influence their responses to acidification, allowing for an interspecific comparison to assess the range of potential responses and underlying mechanisms driving resilience or sensitivity to ocean acidification. Previous research has documented considerable variation in coral responses to ocean acidification, both between different species [25,26] and within the same species [27]. Therefore, conducting interspecific comparisons, such as the one undertaken in this study focusing on 4 species with distinct skeletal architectures, remains crucial. Such comparisons allow for the identification of differential physiological, structural, and molecular strategies employed by corals under acidified conditions, shedding light on the traits that may confer resilience or sensitivity and providing a broader perspective on potential ecosystem-level responses. We simulated an ocean acidification habitat for these coral samples with pH values fluctuating rhythmically between 7.8 during the day and 7.6 at night (control conditions maintained pH values between 8.2 during the day and 8.0 at night). The response of coral polyps in a colony and their calcium carbonate skeletons to external influences was jointly regulated by the polyp–canal system [28–30]. The polyp–canal system is an internal network that facilitates the transport of nutrients and ions, crucial for coral growth and skeleton formation. This system plays an important role in maintaining the connectivity and resilience of coral colonies, particularly under environmental stress. We collected samples from these 4 corals in our simulation system on Days 0, 3, 6, 9, and 30 for high-resolution micro-CT, SEM-EDS, and transcriptome sequencing (RNA sequencing [RNA-seq]) analyses. Reconstructions of the polyp–canal system were used to visualize the growth process in coral colonies and quantify skeleton loss during the 30-day reduced pH level exposure. Elemental ratios in the coral skeletons were analyzed to determine calcium loss/deposition trends while gene expression changes in the coral skeletome [31,32] revealed impacts on biomineralization [33].

In summary, we present insights into skeleton-forming strategies of these representative coral species that highlight effects of excessive CO_2_ on coral growth, providing a theoretical basis for protecting reef-building corals and restoring acid-affected reefs under global ocean acidification.

## Results

### Erosion process in the skeletal system

We reconstructed the polyp–canal systems [34] in each coral colony of *A. muricata*, *M. foliosa*, *M. capricornis*, and *P. damicornis* samples from Day 0 to Day 30, visualizing their erosion process under reduced pH levels (Fig. 1 and Figs. S1 to S21).

**Fig. 1.**
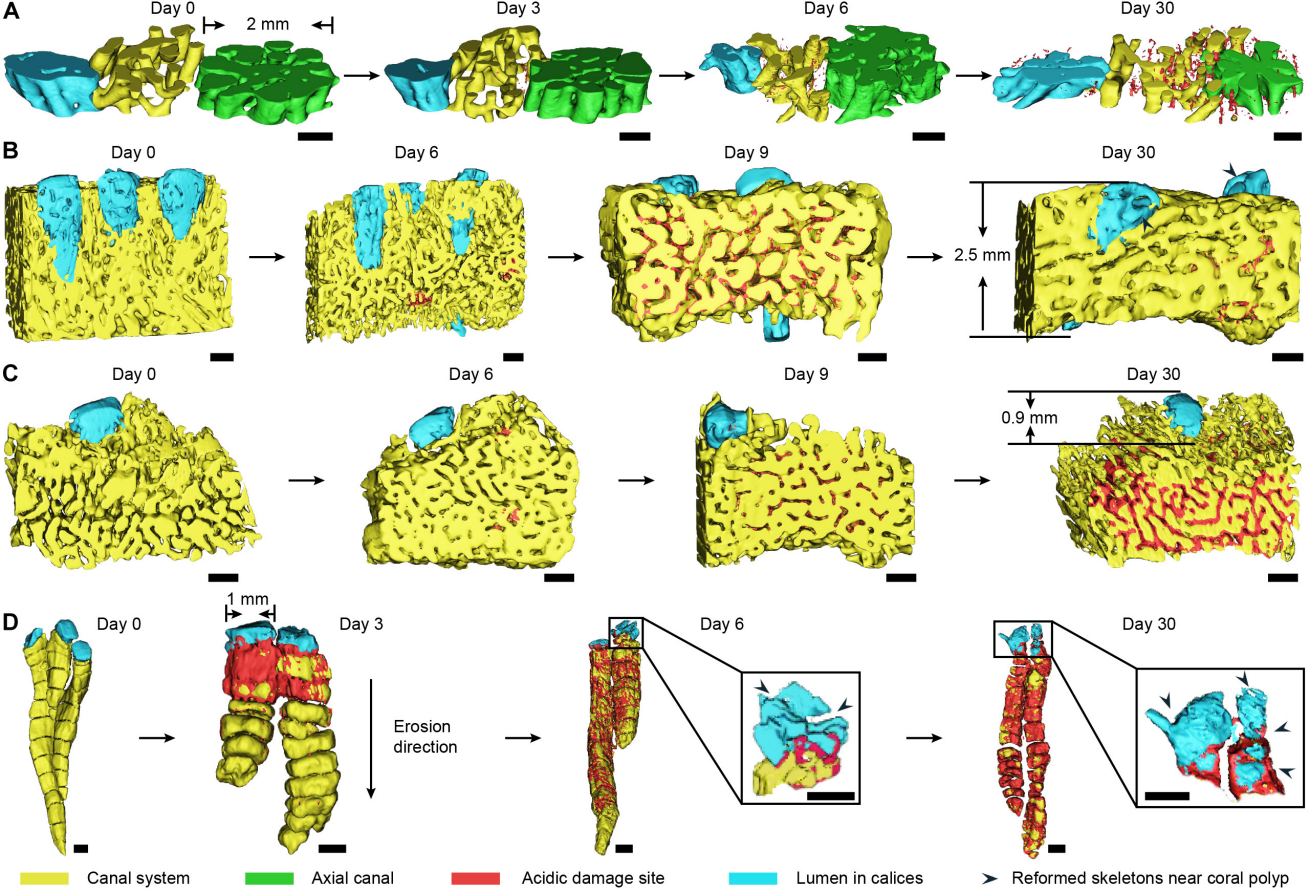
3D reconstructions of polyp–canal systems visualized the corrosion process in reef-building corals under acidic stress. (A) In *A. muricata*, corrosion occurred inside coral skeletons near the canal system on Day 3, and was firstly found in calices on Day 6. Till Day 30, the “cavity-like” acidic damage sites had spread inside skeletons of the colony. Detailed structures can be seen in Figs. S1 to S5, and the formation process is visualized in Fig. S21A. (B) In *M. capricornis*, corrosion occurred on the surface of coral skeletons on Day 6. The acidic damage sites spread on skeletons surrounding the canal system on Day 9, while they were also found in calices near polyps. However, most acidic damage sites were covered by newly formed skeletons on Day 30, and some of them could still be noticed over the canal system away from polyps. Detailed structures can be seen in Figs. S6 to S10, and the formation process is visualized in Fig. S21B. (C) In *M. foliosa*, corrosion occurred on the surface of coral skeletons on Day 6 and could be found in calices on Day 9. The acidic damage sites kept spreading on Day 30, while the skeletons near coral polyps suffered less damages than those away from polyps. Detailed structures can be seen in Figs. S1 to S15, and the formation process is visualized in Fig. S21C. (D) In *P. damicornis*, corrosion occurred over skeletons near the surface of coral colony on Day 3, and spread deep into the colony along the walls and dissepiments around inter-septal spaces. Reformed skeletons occurred in calices on Day 9, covering the acidic damage sites, while irregular reformed skeletons were found in several lumens of the calices, squeezing the lebensraum of coral polyps. Detailed structures can be seen in Figs. S16 to S20, and the formation process is visualized in Fig. S21D. Scale bar: 0.5 mm.

In *A. muricata*, the initial occurrence of acidic damage sites was observed within the skeletons near the canal system on Day 3 (Fig. 1A and Fig. S2). Subsequently, there was a continuous increase in both the quantity and volume of these pore-like sites in the following days. By Day 6, acidic damage sites were also present in axial skeletons and corallites near coral polyps (Fig. S3), leading to internal corrosion and surface erosion across all skeletons within the *A. muricata* colony (Figs. S4 and S5). The adjacent small pore-like acid sites gradually merged to form larger porous sites or fractures within the skeletal structure (Fig. S21A).

In the other 3 coral species, the erosion processes started at the surfaces of coral skeletons. In *M. capricornis* and *M. foliosa*, acid corrosion areas occurred on the surface of the skeletons on Day 6, which increased the volume of the canal system (Fig. 1B and C and Figs. S8 and S13). We could not find any corrosion areas over the corallites until Day 9, and the corrosion areas had already spread over most skeletons around the canal system (Figs. S9 and S14). Fewer corrosion areas remained in the *M. capricornis* colony on Day 30, and the structure of the polyp–canal system was similar to that prior to reduced pH stress (Figs. S10 and S21B). A small amount of irregular reformed skeletons caused tiny gaps in the original canal system and lumen in the calices (refers to the oral cavity enclosed by the corallite within the calices, as illustrated in Fig. S21D) [29,30] of the coral colony (Fig. 1B). However, the corrosion areas surrounding the canal system continued to expand until Day 30 in *M. foliosa* (Fig. 1C), while the corrosive impact near coral polyps was notably less pronounced during this period (Fig. S21C). The polyp–canal system in *M. foliosa* suffered serious damage under reduced pH levels, which had implications for coral growth (Fig. S15).

Corrosion areas first occurred in *P. damicornis* on Day 3 over the walls of coral calices and dissepiments near the surface of the coral colony (Fig. 1D and Fig. S17). From Day 3 to Day 6, the corrosion areas spread inside the colony along the coenosteums around inter-septal spaces in each calyx (Fig. S21D). The corrosion areas near coral polyps became smaller and the appearance of reformed skeletons fragmented the lumen in the calices (Fig. S18). On Day 30, the network of inter-septal spaces in the *P. damicornis* colony had become seriously damaged, corrosion areas covered most of the internal skeletons, and some dissepiments and coenosteums were broken off, causing adjacent inter-septal spaces to merge into larger ones (Fig. 1D and Fig. S20). In contrast, more reformed skeletons occurred in several lumens of the calices, further squeezing the lebensraum of these polyps (Fig. 1D and Fig. S21D).

In conclusion, under reduced pH levels, acid corrosion areas initiated inside the coral skeletons in *A. muricata* and started from their surfaces in *M. capricornis*, *M. foliosa*, and *P. damicornis* (Fig. S21). Among these 4 species, the polyp–canal systems of *A. muricata* and *M. capricornis* were less affected during the 30-day ocean acidification simulation. In addition, we found that skeletons close to coral polyps were less affected by reduced pH levels, with corrosion appearing later in those regions than in other regions of the colony, except in *P. damicornis*. It suggests that ocean acidification primarily affects the dissolution of pre-existing skeletons rather than inhibiting new calcification.

### Skeleton and calcium loss

**Fig. 2.**
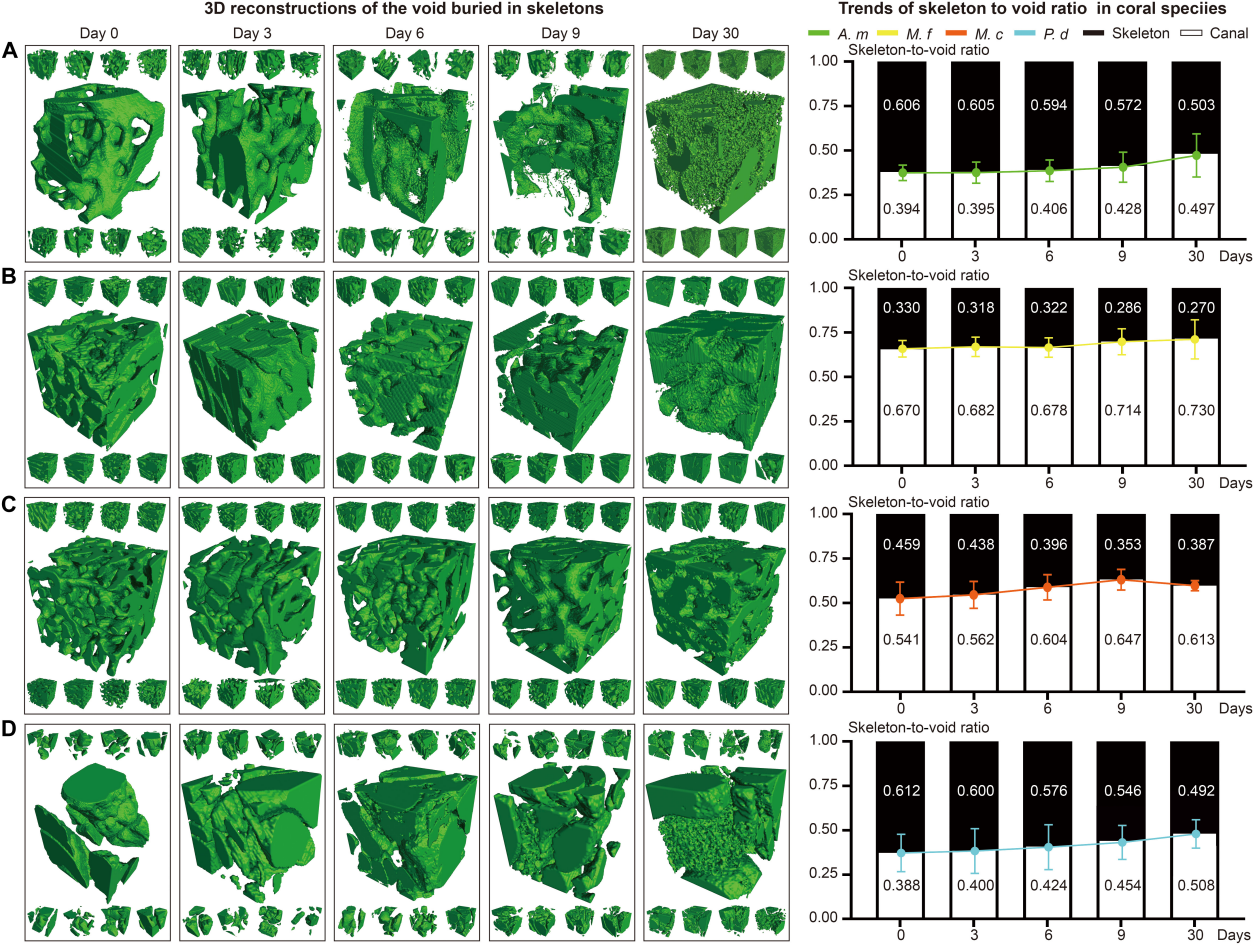
Skeleton-to-void ratio of coral colony revealed the skeleton loss under acidic stress. The green areas in (A) to (D) are the 3D reconstructions of void, which are buried in the skeletons. The analytical error is < ± 1 mm^3^; the error bars represent the standard deviation (SD) for each dataset. A paired-sample *t* test was conducted to assess the *P* values between different time points, reflecting changes in skeleton-to-void ratio of coral samples under lower pH conditions. The results were as follows: Day 0 vs. Day 3, *P* > 0.05; Day 0 vs. Day 6, *P* > 0.05; Day 0 vs. Day 9, *P* < 0.01 (significant difference); Day 0 vs. Day 30, *P* < 0.001 (significant difference). (A) The volume of skeletal matter as a percentage of total coral volume in *A. muricata* colony decreased from 60.6% on Day 0 to 50.3% on Day 30. (B) The volume of skeletal matter as a percentage of total coral volume in *M. foliosa* colony decreased from 33.0% on Day 0 to 27.0% on Day 30. (C) The volume of skeletal matter as a percentage of total coral volume in *M. capricornis* colony decreased from 45.9% on Day 0 to 35.3% on Day 9, and rebounded to 38.7% on Day 30. (D) The volume of skeletal matter as a percentage of total coral volume in *P. damicornis* colony decreased from 61.2% on Day 0 to 49.2% on Day 30.

We quantified skeletal loss during the 30-day acidification experiment by analyzing the skeleton-to-void ratio (Fig. S22A and Table S1). We randomly selected 9 1 mm × 1 mm × 1 mm cuboid areas in each sample and reconstructed their polyp–canal systems. The trends in the average skeleton-to-void ratio in each coral species all decreased with experiment time (Fig. 2). The average volume of canals, as a percentage of the total coral volume in *A. muricata* increased from 39.4% to 49.7%, which meant that nearly 17.0% (10.3% out of 60.6%) of the calcareous skeleton in this colony was lost during the reduced pH level experiment (Fig. 2A). In *M. foliosa*, the canal volume as a percentage of total volume increased from 67.0% to 73.0%, while about 18.2% (6.0%/33.0%) of the coral skeleton was corroded. We observed that the majority of skeleton loss occurred within the first 9 days, with significantly lower losses observed after Day 9 (Fig. 2B). In *M. capricornis*, the canal volume ratio increased from 54.1% to 64.7% between Days 0 and 9 and decreased to 61.3% on Day 30. The volume of coral skeleton increased abnormally between Days 9 and 30, which meant that more skeleton was formed than was eroded during the experiment. The skeleton loss was 23.1% (10.6%/45.9%) on Day 9 and decreased to nearly 15.7% (7.2%/45.9%) on Day 30 (Fig. 2C). As in *P. damicornis*, the canal volume ratio continued to increase, from 38.8% to 50.8%, and the approximately uniform skeleton loss was 19.6% (12%/61.2%) in total (Fig. 2D).

**Fig. 3.**
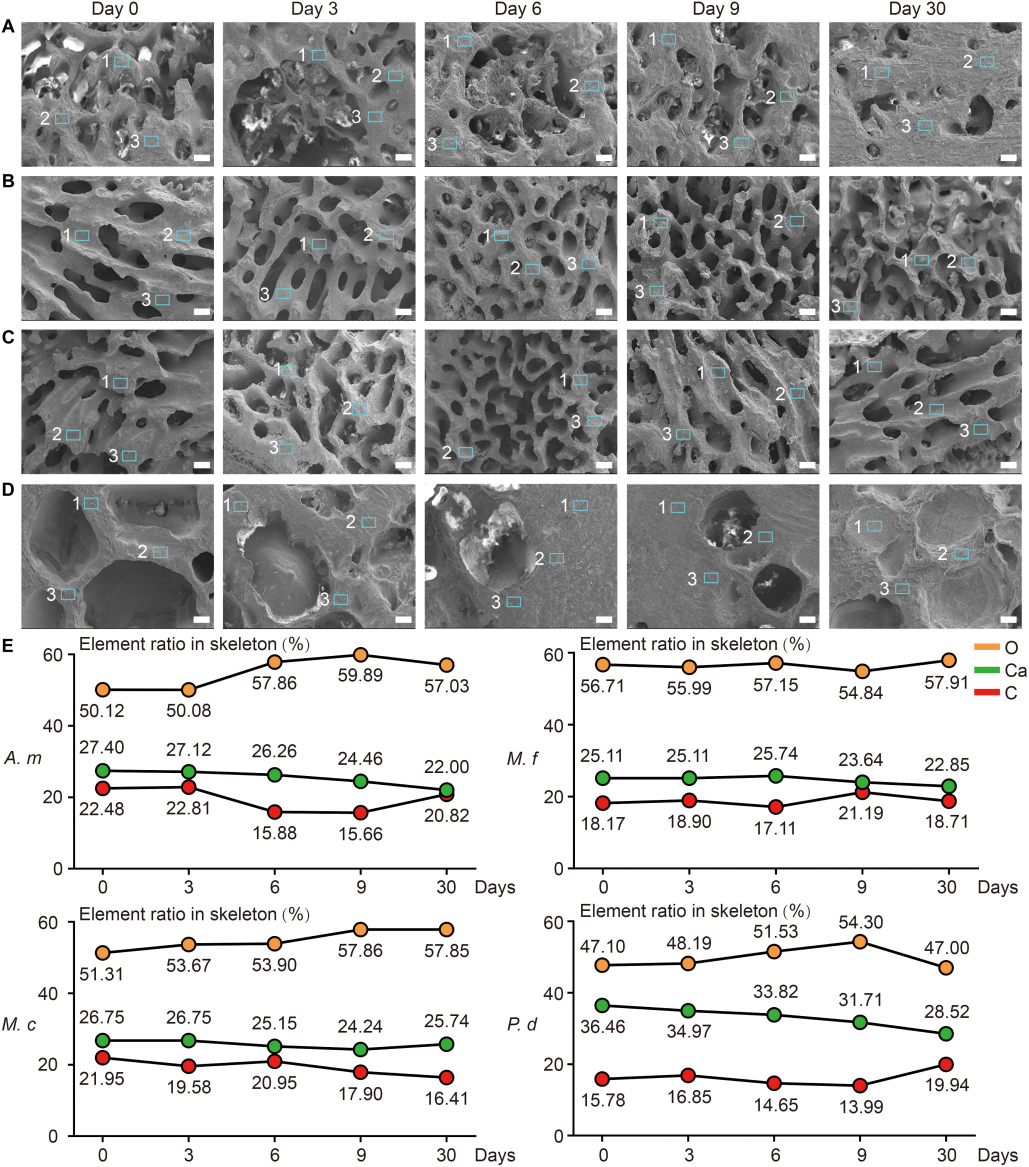
Changes of skeleton element ratio revealed the Ca^2+^ loss in coral colony under acidic stress. (A to D) SEM figures of (A) *A. muricata*; (B) *M. foliosa*; (C) *M. capricornis*; and (D) *P. damicornis* from Day 0 to Day 30, and the signals 1, 2, and 3 in each figure are the sampling sites for the EDS test. (E) The atomic ratio of Ca decreased 5.40% in *A. muricata* (*A.m*), 2.26% in *M. foliosa* (*M.f*), 1.01% in *M. capricornis* (*M.c*), and 5.94% in *P. damicornis* (*P.d*). A paired-sample *t* test was conducted to assess the *P* values between different time points, reflecting changes in Ca elemental composition of coral samples under lower pH conditions. The results were as follows: Day 0 vs. Day 3, *P* > 0.05; Day 0 vs. Day 6, *P* > 0.05; Day 0 vs. Day 9, *P* < 0.05 (significant difference); Day 0 vs. Day 30, *P* < 0.01 (significant difference). The results indicate that there were no significant changes during the first 6 days, suggesting a potential buffering capacity in corals against short-term lower pH stress. However, significant changes began to manifest from Day 9 and were more pronounced by Day 30, indicating the cumulative effect of prolonged lower pH exposure and potential failure of internal buffering mechanisms over time. Scale bar: 0.2 mm.

Calcium (Ca), oxygen (O), and carbon (C) are the main elements present in the corallite of reef-building corals. We used SEM-EDS to obtain the variations in the element atomic ratios of the 4 coral species undergoing reduced pH levels for 30 days (Fig. 3A to D, Fig. S22b, and Table S2). For all 4 coral species, the atomic ratio of Ca decreased between Days 0 and 30 (Fig. 3E and Figs. S23 to S26). Due to the loss of Ca^2+^, the atomic ratio of Ca decreased between Days 0 and 30, with rates of decrease of 5.40% in *A. muricata*, 2.26% in *M. foliosa*, 1.01% in *M. capricornis*, and 5.94% in *P. damicornis* (Fig. 3 and Table S3). Against the background of these decreasing trends, the Ca atomic ratio remained constant between Days 0 and 3 in *M. foliosa* and *M. capricornis*, and appeared to slightly rebound on Day 6 in *M. foliosa* (from 25.11% to 25.74%) and on Day 30 in *M. capricornis* (from 24.24% to 25.74%).

### Gene expression changes in the skeletome

**Fig. 4.**
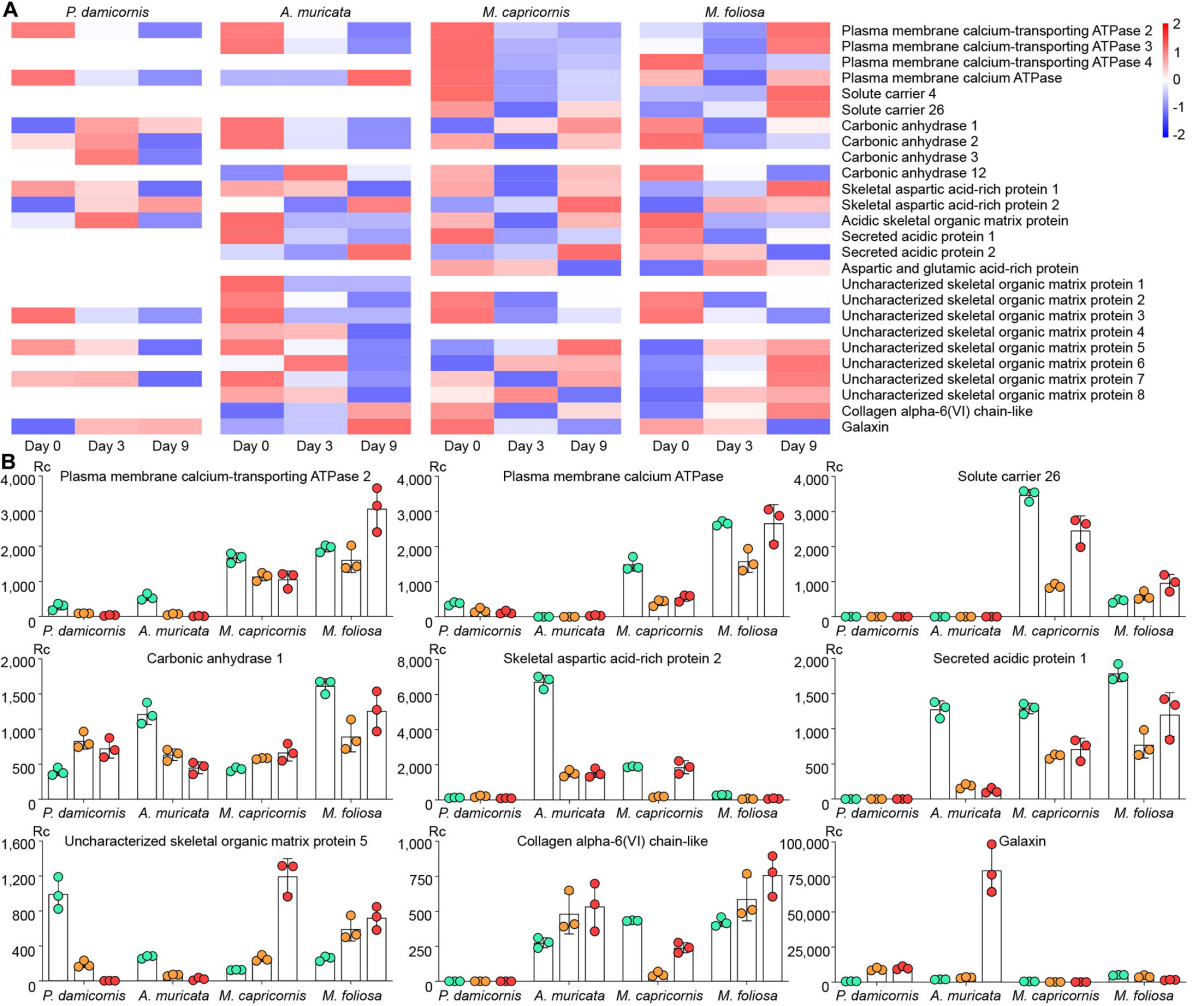
Gene expression changes of skeletome in the 4 reef-building corals under acidic stress. (A) The disparity in gene expression of skeletome on Day 0, Day 3, and Day 9 was shown with a heatmap. There were 3 replicates per time point per species, and the quantities shown in the figure are the average values of the 3 replicates (detailed data can be found in Table S3). (B) Gene expression changes of 9 main proteins. Rc means read count, *P* < 0.001. The 9 genes were selected based on the heatmap analysis in (A), which identified those with the most prominent inter-species differences in gene expression under lower pH conditions. Each coral species has 3 columns above its label, representing the average gene expression levels on Day 0, Day 3, and Day 9, respectively. The 3 filled circles corresponding to each column represent the expression levels of the 3 replicate samples for that day.

The RNA-seq tests showed that the expressions of most genes in these 4 coral species were affected by ocean acidification on Day 3, which we believe to be the stress condition of these coral–symbiodinium holobionts [35]. The expression of most genes returned to a steady state on Day 9; however, the skeletome in the coral polyps still suffered significant effects (Fig. 4). Among the proteins involved in coral skeleton formation and calcium transportation, the following 5 categories suffered most from acidification (Fig. S7 and Table S3): (a) Transporting proteins like plasma membrane calcium-transporting adenosine triphosphatase (PMC-t ATPase), plasma membrane calcium ATPase (PMC ATPase), and solute carrier (SC). The ATPases are involved in calcium transportation through the membranes of entodermic and calicoblastic cells, while SC helps to transport bicarbonate. (b) Carbonic anhydrase (CA). These are enzymes for the interconversion of carbon dioxide and bicarbonate that can regulate their balance to affect the pH of coral cells. (c) Acid-rich proteins (ARPs), like skeletal aspartic acid-rich protein (SAARP), acidic skeletal organic matrix protein (ASOMP), secreted acidic protein (SAP), and aspartic and glutamic acid-rich protein (AGARP). These ARPs can deposit calcium carbonate from seawater directly for coral skeleton formation. (d) Skeletal organic matrix proteins (SOMPs), which control coral skeleton formation through bio-mineralization. (e) Adhesion proteins like galaxin and collagen alpha-6(VI) chain-like (α-C6), which cement the calcium carbonate crystals to each other and to coral skeletons.

In the polyps of *P. damicornis*, nearly half of the skeletome mentioned above were almost unaffected by reduced pH levels, including SC, SAP, AGARP, SOMP-1/2/4/6/8, and α-C6. The expression of ATPases decreased continuously from Days 0 to 3 and Days 3 to 9. The expressions of CA and ASOMP increased from Days 0 to 3 and decreased from Days 3 to 9. The expression of SAARP-1 decreased while that of SAARP-2 increased from Days 0 to 9. The expressions of SOMP-3/5 decreased continuously from Days 0 to 9, while the expression of SOMP-7 increased from Days 0 to 3 and decreased from Days 3 to 9. The expression of galaxin increased continuously from Days 0 to 9 (Fig. 4A). These findings suggest that the polyps of *P. damicornis* exhibit potential for adaptation to ocean acidification, as the expression of approximately half of its skeletome genes remains largely unaffected by reduced pH levels. The ability to transport calcium through cells was reduced on Day 3, while the ability to adjust pH and the sedimentation/adhesion of calcium carbonate were significantly improved. On Day 9, most of the biomineralization abilities were significantly inhibited, except for continuous increases in the adhesion of calcium carbonate (Fig. 4B).

In *A. muricata*, the expression of SC was almost unaffected by reduced pH levels. The expressions of ATPases, CA, ARPs, and SOMPs decreased continuously from Days 0 to 9. The expressions of α-C6 and galaxin increased continuously from both Days 0 to 3 and Days 3 to 9 (Fig. 4A). The lower pH tolerance of *A. muricata* seems weaker than that of *P. damicornis*, and most of its biomineralization processes were markedly inhibited by acidification. However, the secretion of adhesion proteins was greatly increased, which potentially promoted binding among the calcium carbonate crystals that maintain the toughness of the coral colony despite continuous losses of calcium and skeleton (Fig. 4B). This pattern may have mitigated the effects of the retardation in skeleton formation.

In *M. capricornis*, the expressions of ATPases and α-C6 decreased from Days 0 to 3 and rebounded slightly from Days 3 to 9. The expressions of SC, CA, SAARP, ASOMP, and SAP decreased from Days 0 to 3 and sharply rebounded from Days 3 to 9. The expressions of AGARP and galaxin decreased continuously from Days 0 to 9. The expressions of SOMPs showed a downward trend on Day 3 but significantly rebounded on Day 9 (Fig. 4A). The biomineralization ability of *M. capricornis* was significantly inhibited on Day 3 but continued to intensify until Day 9. Despite the compensatory increase in biomineralization processes to counteract skeleton and calcium losses under reduced pH levels, continuous reductions in the secretion of adhesion proteins may render coral colonies more fragile (Fig. 4B).

In *M. foliosa*, the expressions of ATPase and SC decreased from Days 0 to 3 and sharply rebounded from Days 3 to 9. The expressions of CA, ARPs, and galaxin showed a downward trend from Days 0 to 9. The expressions of SOMP-2/3 decreased while those of SOMP-5/6/7/8 increased from Days 0 to 9. The expression of α-C6 increased continuously from Days 0 to 9 (Fig. 4A). In the *M. foliosa* colony, the abilities to precipitate calcium carbonate from seawater and adjust pH were inhibited under reduced pH levels, while the oriented precipitation of calcium carbonate crystals and the transmembrane transport of calcium and bicarbonate were promoted (Fig. 4B).

## Discussion

### Survival strategies of 4 investigated corals

This work revealed distinct survival strategies of *A. muricata*, *M. foliosa*, *M. capricornis*, and *P. damicornis* under reduced pH conditions using micro-CT, SEM-EDS, and RNA-seq tests. These species-specific responses highlight the complexity of predicting coral reef futures under ocean acidification [5,36].

The “cavity-like” corrosion pattern observed in *A. muricata* represents a notable strategy, differing markedly from the other 3 species. Corrosion pores first occurred inside the coral skeletons, which gradually extended to the central area from the near-surface area. During this process, there were no obvious cracks or channels on the surface of the coral skeleton and the polyp–canal system regulating coral growth was minimally affected (Fig. 1A and Fig. S21A). Under reduced pH levels, the number and volume of corrosion pores in the skeletons continued to increase, while the skeleton-to-void ratio in the coral colony and the calcium element ratio in the skeletons decreased (Figs. 2A and 3A and E) and the integrity of the growth-regulating network was maintained. Additionally, the gene expression of most skeletome-related genes in the coral polyps involved in skeleton formation significantly decreased (Fig. 4), a finding that resonates with transcriptomic studies on other *Acropora* species under acidification stress; for instance, certain calcification-related genes are down-regulated in *Acropora millepora* during the initiation of calcification under elevated, suggesting that disrupting the calcification machinery is a common response within this genus [37]. However, our finding that *A. muricata* specifically up-regulates galaxin and α-C6 gene expressions (Table S3), potentially to maintain skeletal toughness despite internal dissolution, thereby enhancing the protective capacity of the coral colony, suggests a more nuanced mechanism for resilience not previously highlighted. Since the polyp–canal system is the basis for the sustainable growth of reef-building corals [30], this survival strategy—of sacrificing internal structures and calcium inside the skeletons to protect the basic canal network—helps *A. muricata* maintain its growth patterns. The cavity-forming strategy may allow *A. muricata* to maintain polyp function and colony integrity, potentially enhancing its resilience compared to species with more severe skeletal degradation. This strategy could confer greater resistance to ocean acidification compared to species exhibiting more surface-driven degradation, thereby maintaining growth patterns essential for reef accretion.

The erosion patterns observed in the other 3 coral species resemble those seen in osteoporosis [38]. They are reflected in the insufficient synthesis of adhesion proteins in *M. capricornis*, which may lead to a decrease in skeletal toughness (Fig. 4). The low bone mass and calcium loss in *M. foliosa* decrease the quality and hardness of the coral colony (Figs. 2 and 3). The microstructure of the coral skeletons is destroyed and become disordered, which may affect the mechanical strength of *P. damicornis* (Fig. 1D and Fig. S21D). Coral polyps form new skeletons over surrounding corrosion areas through biomineralization, similar to the function of osteoblasts [39].

Despite their vulnerabilities, each species exhibits unique compensatory or adaptive mechanisms, reflecting different strategies to cope with acidification stress. *M. capricornis* achieves self-protection under reduced pH levels primarily through its high rate of skeleton formation. The increases in CA, SAARP, ASOMP, SAP, and some SOMPs (uncharacterized SOMPs) from Days 3 to 9 facilitated the formation of new coral skeletons, potentially alleviating the impact of skeleton and calcium losses on the coral colonies (Fig. 1B and Fig. S21B). This strategy also protects the polyp–canal system in *M. capricornis* and maintains the sustained growth of a coral colony under reduced pH levels (Figs. 2 and 3). However, the decreasing gene expressions of galaxin and α-C6 (collagen alpha-6 chain-like) affect the toughness of coral skeletons. The erosion pattern resembling osteoporosis renders *M. capricornis* colonies more susceptible to physical damage under the influence of acidification (Table S3).

Despite the rapid skeleton and calcium losses observed in *M. foliosa* colonies due to acidification damage from Days 6 to 9, there was a significant increase in the expressions of ATPases, SC, SAARP, and some SOMPs by Day 9. This increase effectively mitigate further skeleton loss for the remainder of the experiment (Figs. 2 to 4). This phenomenon was mostly pronounced over or near the walls of calices, while other skeletons in the *M. foliosa* colony still experience acidic damage (Fig. 1C and Fig. S21C). Such molecular responses, involving the up-regulation of genes related to ion transport and organic matrix synthesis, are recognized compensatory mechanisms observed in corals under environmental stress, including acidification and thermal challenges, as shown in studies on the coral holobiont’s response [40,41]. Our results add to this understanding by demonstrating species-specific timing and emphasis in these molecular responses within the *Montipora* genus. These differing responses might influence their competitive interactions or distribution in acidified environments, with *M. capricornis* potentially outcompeting *M. foliosa* under moderate acidification, underscoring the importance of considering interspecific competition dynamics in future reef scenarios.

We found that *P. damicornis* polyps exhibit better tolerance to lower pH levels, as the expressions of most genes are minimally affected by the acidic habitat (Fig. 4). Only half of the genes related to skeleton formation suffered from the erosion process on Day 9, which slightly reduced the biomineralization of the coral polyps (Table S3). This suggests a higher physiological threshold for ocean acidification stress in this species, consistent with some studies indicating that *Pocillopora* species can be relatively resilient [25]. In contrast, morphological analysis revealed substantial costs. Some of the dissepiments and coenosteums around inter-septal spaces became thinner or even fractured due to the skeleton and calcium losses. The thick and dense corallites at the surface of the coral colony, where polyps live, were less affected, as new skeletons were formed in the calices that filled part of the corrosion areas over the corallites. However, several new skeletons were not formed along the original patterns, which separated the lumen in calices into fragmented cubes, reducing the living spaces for polyps (Fig. 1D and Fig. S21D). Since the coenosarcus between polyps are more likely to break down in an acidic habitat [42], these irregularly formed skeletons will further aggravate the polyp detachment [43] of *P. damicornis*. These factors potentially lead to the disorder and destruction of skeletal structures, thereby affecting the mechanical strength of coral colonies. The formation of irregular skeletons could compromise long-term colony survival and mechanical strength despite apparent molecular resilience, potentially reducing its ability to withstand physical stressors such as wave action. This highlights that transcriptomic stability alone does not guarantee ecological success under chronic stress.

Revealing these varied species-specific strategies, from internal structural sacrifice (*A. muricata*) and rapid compensatory growth (*M. capricornis*) to localized repair (*M. foliosa*) and tolerance with structural costs (*P. damicornis*), is crucial for predicting and managing coral reef ecosystems under intensifying ocean acidification. For instance, identifying species like *A. muricata* with potentially unique resilience mechanisms might guide restoration efforts, while understanding the specific vulnerabilities of species like *M. capricornis* (toughness reduction) or *P. damicornis* (irregular growth) highlights the need for management actions that mitigate additional stressors (like physical damage or pollution). Therefore, understanding species-specific responses to acidification can inform targeted conservation efforts, such as prioritizing the protection of species with higher resilience or developing assisted improving programs for vulnerable species.

### The maintenance mechanism of coral skeleton under reduced pH levels

We found that, in *A. muricata*, *M. capricornis*, and *M. foliosa*, skeletons near coral polyps experienced acidic damage later and to a lesser extent than other skeletons within the same colony. As in *P. damicornis*, although the corrosion areas occurred first over corallites at the surface of the coral colony around polyps, most of these areas were filled with newly formed skeletons during long-term reduced pH levels, while other skeletons inside the colony away from polyps suffered serious damage (Fig. 1). This reveals that lower ambient pH did not initially influence coral biomineralization but could lead to the dissolution of pre-existing skeletons. Other skeletons farther away from coral polyps were more severely eroded, and this observation may indicate that the transport of calcium, SOM, calcification fluid, and other skeleton-forming materials via the gastrovascular canals was inhibited by reduced pH levels (Fig. S21).

Additionally, we found that the expressions of various skeletome-related genes increased in both *M. capricornis* and *M. foliosa* by Day 9, thereby enhancing their biomineralization ability and maintaining biomineralization in areas near the polyps under lower pH conditions. However, the skeleton protected by this mechanism was much greater in *M. capricornis* samples compared to *M. foliosa* (Fig. 4). We believe that this difference is primarily due to variations in calyx volume and density. The calices in *M. capricornis* are larger and more densely distributed and border most of the skeletons in the colony, while the calices in *M. foliosa* are much smaller, and the pre-existing skeleton farther from the polyps is more susceptible to dissolution under lower ambient pH (Figs. S11 to S20).

## Materials and Methods

### Sample collection

Coral colonies of *A. muricata*, *M. capricornis*, *M. foliosa*, and *P. damicornis* were collected in 2018 from shallow reef sites at water depths of 5 to 10 m around the Xisha Islands (15°40′ to 17°10′N, 111° to 113°E). All samples were found in tropical shallow reefs where the daily mean temperature was 23.2 to 29.2 °C. All coral colonies were collected from the same reef region with similar pH values and variation ranges (pH 8.0 to 8.2). Three colonies of each species were collected, with approximate sizes as follows: *A. muricata*, 15 × 15 × 25 cm, 20 × 20 × 28 cm, and 12 × 12 × 20 cm; *M. capricornis*, 28 × 25 × 18 cm, 25 × 20 × 15 cm, and 22 × 18 × 12 cm; *M. foliosa*, 18 × 16 × 10 cm, 23 × 20 × 8 cm, and 20 × 18 × 8 cm; *P. damicornis*, 20 × 20 × 15 cm, 18 × 16 × 12 cm, and 22 × 20 × 18 cm. Before the experiments, all coral samples were maintained intact and housed in our laboratory coral tank for at least 3 months under conditions designed to mimic their natural habitat in the South China Sea.

### Coral culture system

The coral samples were temporarily cultured in a standard RedSea tank (redsea575, Red Sea Aquatics Ltd., London, UK) before and after micro-CT, following the Berlin method. The temperature was kept at 25 °C and the salinity was 35‰ (specific gravity = 1.025). The culture system was maintained using a Protein Skimmer (regal250s, Honya Co. Ltd., Shenzhen, China), a water chiller (tk1000, TECO Ltd., Taiwan, China), 3 coral lamps (AI, Red Sea Aquatics Ltd., London, UK), 2 wave devices (VorTechTM MP40, EcoTech Marine Ltd., Bethlehem, USA), and a calcium reactor (Calreact 200, Honya Co. Ltd., Shenzhen, China). The coral cultivation and maintenance system was prepared for the full duration of the experiment. In the experiment, we utilized 4 sets of such cultivation systems, with 3 sets used for parallel experimental groups to simulate ocean acidification, and the remaining set used for the non-acidified control group. The corals were fragmented into uniform-sized pieces to ensure consistency across experimental conditions.

### Simulation of ocean acidification

The experiment was conducted in 200-l (40 cm × 50 cm × 100 cm) tanks with a seawater flow rate of 10 l/min. Carbonate chemistry in the coral tanks was manipulated by bubbling CO_2_ through the water using CO_2_ reactor. The control tanks were maintained at ambient pH (8.0 to 8.2), while the acidified tanks were set to a target pH of 7.8. To simulate natural diurnal pH fluctuations observed in coral reef environments, a microfluidic device periodically released small quantities of acetic acid into the acidified tanks, causing the pH to decrease to 7.6 during the night and return to 7.8 during the day. This approach was adopted to mimic natural pH variability. During the simulation process, pH was monitored continuously using a pH electrode (Fig. S28) connected to a monitoring system. Discrete water samples were collected to measure total alkalinity (TA) via an alkalinity titrator, and calcium (Ca^2+^), carbonate (CO_3_^2−^), and bicarbonate (HCO^3−^) ion concentrations using ion chromatography (Table S5). The partial pressure of CO_2_ (pCO_2_) was calculated from pH and TA measurements.

### Micro-CT test

Coral samples from the South China Sea were analyzed using 3D models constructed with a 230-kV latest-generation x-ray microfocus computed tomography system (Phoenix v|tome|x m; General Electric, GE; at Yinghua NDT, Shanghai, China; Table S4). Two-dimensional image reconstructions of each specimen from the matrices of scan slices were assembled using proprietary software from GE. We conducted micro-CT tests on 3 samples per species at each time point, with the samples being collected from different colonies and exhibiting nearly identical sizes. The sample sizes of the 4 coral species were as follows: *A. muricata*, length of about 4 cm and a diameter of about 0.8 cm; *M. capricornis* and *M. foliosa*, dimensions of about 2 cm × 2 cm × 0.5 cm; and *P. damicornis*, dimensions of about 1.5 cm × 1 cm × 1.5 cm. The micro-CT experiment requires no sample preparation, enabling direct examination of living specimens while ensuring that all samples remain viable throughout the imaging process. This non-destructive approach offers a distinct advantage for studying coral growth patterns, preserving the natural state of the specimens during analysis. The coral samples analyzed using micro-CT included both the skeleton and tissue. The scan results provided detailed structural information about the skeleton and canal. The grayscale values from the imaging data allowed for the localization of tissue (such as polyp) for visual reconstruction. The SEM-EDS test was conducted on skeleton samples exclusively. This test was focused on analyzing elemental variations within the coral skeleton. The RNA-seq test utilized tissue obtained from the coral. The SEM-EDS test and RNA-seq test respectively complement and support the experimental results and findings revealed by the micro-CT test, from the perspectives of skeleton and tissue.

### Micro-CT reconstruction

Slice data derived from the scans were then analyzed and manipulated using 3D reconstruction software. Polyp–canal system reconstructions and skeleton-to-void ratio measurements were performed using VG Studio Max [28,44] (v3.3.0). The 3D reconstructions were created following the method previously described. Images of the reconstructions were exported from Mimics and VG Studio Max and finalized in Adobe Photoshop CC 2019 and Adobe Illustrator CC 2019.

### Identification of acidic damage sites

The identification of acidified regions was primarily based on comparing the structures in the layer-by-layer slices of reconstruction data and measuring the grayscale values (Fig. S30). 3D reconstructions of the samples were obtained, followed by segmented slices (10 μm per layer) along the growth axis for subsequent analysis and identification.

By comparing tens of thousands of reconstructed slices from control samples and experimental samples (3, 6, 9, and 30 days), we observed numerous irregular surface and internal skeletal damage or pore-like structures in the experimental group, which were absent in the control group. These structural features increased in number, expanded in volume, or gradually merged with prolonged acidification. In some *Pocillopora* samples, additional irregular skeletal structures were observed inside calices. By capturing and identifying these characteristic features, we were able to preliminarily identify and reconstruct changes of skeleton or polyp–canal system in coral colonies resulting from lower pH environments (Fig. S30B).

Branchlets from the same coral species and growth period typically displayed consistent grayscale distribution (Fig. S30A). The grayscale value is an indicator parameter in micro-CT monitoring, associated with the material composition and density of the sample, while higher density corresponds to a greater grayscale value. In this work, noticeable differences were observed between acidified and control samples. Comparison between the experimental and control groups revealed that grayscale values in acidified regions markedly decreased compared to the same locations in normal skeletons, although they remained higher than those in polyp–canal systems. In samples subjected to prolonged acidification, some acidified pores exhibited larger volumes (commonly observed in *Acropora* samples), or the acidified regions displayed structural properties similar to pre-existing canals (often occurring in *Montipora* samples). In such cases, distinguishing between naturally occurring canals and acidified regions through structural comparison alone was challenging. Thus, grayscale measurements were employed to differentiate acidified damage from pre-existing polyp–canal systems, thereby excluding non-target areas based on the initial analysis (Fig. S30B). This 2-step method was utilized to mark areas of erosion caused by acidification in the studied coral colonies. This allowed us to produce a sectional diagram as shown in Fig. S21 and further construct the 3D structural changes of the polyp–canal system under lower pH conditions, as presented in Fig. 1.

### Skeleton-to-void ratio measurement

The calculation of the skeletal matter to void volumetric ratio of coral samples, referred to as “skeleton-to-void ratio”, was conducted using VG Studio Max 3.3 [45]. In this study, this method was employed to quantify the volume of the skeleton and canal network within each sample, enabling the determination of the proportion of total volume occupied by the skeleton. This approach allowed us to quantitatively evaluate the effects of lower pH conditions on the coral skeleton and the polyp–canal system. The detailed procedures are outlined below. Initially, the “surface determination” function was utilized to differentiate between the areas of reconstructed skeleton and canal system in the colony. Subsequently, the volume of the reconstructed skeleton was calculated and the “erode/dilate” mode was employed to encompass the entire area of both the skeleton and canals. Following this, the “porosity/inclusion analysis module” was used to reconstruct the lumen of the canal system for volume calculation. Consequently, with volumes obtained for both the skeleton and internal canal system, we were able to determine a skeleton-to-void ratio for each sample. Three samples per species per time point were measured along with 3 technical replicates per sample in this study. These areas were randomly selected from coral samples to ensure representation of skeletal variability across the colony.

### SEM-EDS test

The SEM-EDS test was conducted on skeleton samples, which were argon-ion polished prior to analysis. This test was focused on analyzing elemental variations within the coral skeleton. For the SEM-EDS analysis, 3 mm × 3 mm × 1 mm cubes were extracted from the central region of each coral colony utilized in the micro-CT examination. To ensure the precision of the SEM analysis, conductive coatings were meticulously applied to polished 3 mm × 3 mm cross-sections of each sample. Subsequently, the coral samples underwent SEM imaging to capture cross-sectional views of their skeletons [46]. Following this, 3 random rectangular areas measuring 0.15 mm × 0.10 mm were selected from the SEM images of each sample for EDS scanning [47]. The atomic ratios of Ca, O, and C within each area were determined and averaged across all samples for statistical analysis purposes. A total of 3 samples per species per time point were analyzed using SEM-EDS.

### RNA-seq test

#### Sampling

The simulation device was turned on, then when the tank pH stabilized at 7.6 to 7.8, the test started on Day 1. Before the simulation, samples were denoted as “Day 0”, then denoted according to the experimental day.

#### Total RNA extraction

Three samples per species per time point were measured along with 3 technical replicates per sample in this test, and the 3 samples from each colony were treated independently for the transcriptome sequencing. In each coral, triplicate biological samples were isolated from 3 healthy branches in the same colony to ensure that enough high-quality RNA (>15 μg) could be obtained for a PacBio cDNA library and 3 Illumina cDNA libraries. All the RNA extraction procedures followed the manufacturer’s instructions. The total RNA was isolated with TRIzol LS Reagent (Thermo Fisher Scientific, 10296028, Waltham, MA, USA) and treated with DNase I (Thermo Fisher Scientific, 18068015, Waltham, MA, USA). The high-quality mRNA was isolated with a FastTrack MAG Maxi mRNA Isolation Kit (Thermo Fisher Scientific, K1580-02, Waltham, MA, USA). The RNA extraction procedure was performed according to the following instructions: (a) grind the coral samples into small pieces (submerged in liquid nitrogen at all times); (b) add TRIzol LS reagent at a sample-to-reagent ratio of about 1:3; (c) let the samples stand and thaw naturally; (d) continue adding TRIzol LS reagent until the samples are dissolved, and dispense into 50-ml centrifuge tubes; (e) centrifuge at 4 °C and 3,000 rpm for 5 to 15 min; (f) dispense the supernatant into 50-ml centrifuge tubes; (g) add BCP (Molecular Research Center, BP 151, Cincinnati, OH, USA) to the centrifuge tubes at a sample-to-reagent ratio of about 5:1, shake well, and stand for 10 min; (h) centrifuge at 4 °C and 10,500 rpm for 15 min; (i) remove the supernatant, add an equal volume of isopropanol (Amresco, 0918-500ML, Radnor, PA, USA) and mix well, and stand overnight at –20 °C; (j) centrifuge at 4 °C and 10,500 rpm for 30 min and discard the supernatant; (k) rinse twice with 75% ice ethyl alcohol, pure (Sigma-Aldrich, E7023-500ML, Taufkirchen, München, Germany). Finally, extract 3 samples of each coral in equal amounts (total > 10 μg) and mix for PacBio full-length transcriptome sequencing. The remainder (>1.5 μg per sample) was used for Illumina sequencing.

#### Total RNA quality testing

Before establishing the library, the quality of total RNA must be tested. RNA degradation and contamination were monitored by 1% agarose gels electrophoresis; RNA purity (OD260/280 ratio) was checked using a NanoPhotometer spectrophotometer (IMPLEN, CA, USA); RNA concentration was quantified using a Qubit RNA Assay Kit in Qubit 2.0 Fluorometer (Life Technologies, CA, USA); and RNA integrity was assessed using the RNA Nano 6000 Assay Kit of the Agilent Bioanalyzer 2100 system (Agilent Technologies, CA, USA).

#### Illumina cDNA library construction and sequencing

A total amount of 1.5 μg of RNA per sample was used as input material for the RNA sample preparations. Sequencing libraries were generated using NEBNext Ultra RNA Library Prep Kits (E7530L) for Illumina (NEB, Ipswich, MA, USA) following the manufacturer’s recommendations and index codes were added to attribute sequences to each sample. Briefly, mRNA was purified from total RNA using poly-T oligo-attached magnetic beads. Fragmentation was carried out using divalent cations under elevated temperature in NEBNext First Strand Synthesis Reaction Buffer (5×). First-strand cDNA was synthesized using random hexamer primer and M-MuLV Reverse Transcriptase (RNase H^−^). Second-strand cDNA synthesis was subsequently performed using DNA Polymerase I and RNase H. Remaining overhangs were converted into blunt ends via exonuclease/polymerase activities. After adenylation of 3′ ends of DNA fragments, the NEBNext Adaptor with a hairpin loop structure was ligated to prepare for hybridization. To select cDNA fragments preferentially of 250 to 300 bp in length, the library fragments were purified with the AMPure XP system (Beckman Coulter, Beverly, USA). Then, 3 μl of USER Enzyme (NEB, USA) was used with size-selected, adaptor-ligated cDNA at 37 °C for 15 min followed by 5 min at 95 °C before polymerase chain reaction (PCR). Then, PCR was performed with Phusion High-Fidelity DNA polymerase, Universal PCR primers, and Index (X) Primer. Finally, the PCR products were purified (AMPure XP system) and library quality was assessed on the Agilent Bioanalyzer 2100 system. Clustering of index-coded samples was performed on a cBot Cluster Generation System using a TruSeq PE Cluster Kit v3-cBot-HS (Illumina) according to the manufacturer’s instructions. After cluster generation, the library preparations were sequenced on an Illumina HiSeq X Ten platform and paired-end reads were generated.

#### PacBio cDNA library construction and sequencing

An isoform sequencing (Iso-Seq) library was prepared according to the Iso-Seq protocol using the Clontech SMARTer PCR cDNA Synthesis Kit (Clontech Laboratories, now Takara Laboratories, 634926, Mountain View, CA, USA) and the BluePippin Size Selection System protocol as described by Pacific Biosciences (PN 100-092-800-03). Briefly, Oligo(dT)-enriched mRNA was reversely transcribed to cDNA by a SMARTer PCR cDNA Synthesis Kit. The synthesized cDNA was then amplified by PCR using the BluePippin Size Selection System protocol. The Iso-Seq library was constructed by full-length cDNA damage repair, terminal repair, and attaching SMRT dumbbell adapters. The sequences of the unattached adapters at both ends of the cDNA were removed by exonuclease digestion. The obtained cDNA was combined with primers and DNA polymerase to form a complete SMRT bell library. While the library was qualified, the PacBio Sequel II platform was used for sequencing based on the effective concentration and data output requirements of the library.

#### Data filtering and processing

The Illumina sequencing raw reads in fastq format were first processed using in-house Perl scripts. In this step, clean data were obtained by removing reads containing adapter or ploy-N and low-quality reads from raw data. At the same time, the Q20, Q30, GC-content, and sequence duplication levels of the clean data were calculated. All the downstream analyses were based on clean data with high quality.

The PacBio sequencing raw data were processed by SMRTlink v8.0 software. A circular consensus sequence (CCS) was generated from subread BAM files using the following parameters: min_length 50, min_passes 1, and max_length 15,000. CCS.BAM files were output, which were then classified into full-length and non-full-length reads using lima, removing polyA using refine. Full-length fasta files were produced and then fed into the cluster step, which performed isoform-level hierarchical clustering [*n*log(*n*)], followed by final Arrow polishing using the settings hq_quiver_min_accuracy 0.99, bin_by_primer false, bin_size_kb 1, qv_trim_5p 100, and qv_trim_3p 30.

#### Coral and Symbiodiniaceae sequences separation

Aligned consensus reads to coral or Symbiodiniaceae reference genomes were performed using GMAP v2017-06-20 software [48]. The sequences mapped to Symbiodiniaceae reference genomes belonged to Symbiodiniaceae sequences, while sequences mapped to coral reference genomes belonged to coral sequences.

#### Correction and de-redundancy

The RNA-seq data sequenced by the Illumina HiSeq X Ten platform were used to correct additional nucleotide errors in polish consensus sequences obtained in the previous step with LoRDEC v0.7 software [49]. Using CD-HIT v4.6.8 software (parameters: -c 0.95 -T 6 -G 0 - aL 0.00 -aS 0.99), all redundancies were removed in corrected consensus reads to acquire final full-length transcripts and unigenes for subsequent bioinformatics analysis [50].

#### Gene functional annotation

Gene functions were annotated using the following databases: NT (NCBI non-redundant nucleotide sequences), NR (NCBI non-redundant protein sequences), Pfam (protein family), KOG/COG (Clusters of Orthologous Groups of proteins), Swiss-Prot (a manually annotated and reviewed protein sequence database), KEGG (Kyoto Encyclopedia of Genes and Genomes), and GO (Gene Ontology). We used BLAST 2.7.1+ software [51] with the e-value “1e−5” for NT database analysis, Diamond v0.8.36 BLASTX software [52] with the e-value “1e−5” for NR, KOG, Swiss-Prot, and KEGG databases analyses, and the HMMER 3.1 package [53] for Pfam database analysis.

#### Gene structure analysis

ANGEL v2.4 software [54] was used to predict protein CDSs (coding sequences). We used the same species or closely related species-confident protein sequences for ANGEL training and then ran the ANGEL prediction for the given sequences. Usually, the TFs were identified based on the Pfam files of TF families in the AnimalTFDB 3.0 database [55]; however, corals were not included in this database; thus, we identified coral TFs based on the Pfam files of TF families using the hmmsearch program in the HMMER 3.1 package. The SSR of the transcriptomes was identified using MISA v1 [56]. We used 4 tools—CNCI v2 [57], CPC2 v0.1 [58], PfamScan v1.6 [59], and PLEK v1.2 [60]—to predict the coding potential of the transcripts. Transcripts predicted with coding potential by either/all of the above 3 tools were filtered out, and those lacking coding potential were our candidate set of long non-coding RNAs.

#### Gene expression quantification

The full-length transcriptome obtained above was used as the reference background, and then the clean reads of each sample obtained by Illumina sequencing were mapped to it using bowtie2 v2.3.4 software [61]. The alignment results were estimated by RSEM v1.3.0 software [62] to obtain the read count values for each transcript, which were then transferred to FPKM (fragments per kilobase of exon model per million mapped fragments) for analysis of gene expression levels. Pearson correlation coefficients were used to analyze the relationships among samples.

#### Gene differential expression analysis

The unigenes with the same annotation results in the NR database were merged to form a new read count expression matrix. Differential expression analysis of 2 groups was performed using the DESeq2 R package (v1.30.1) [63]. DESeq2 provides statistical routines for determining differential expressions in digital gene expression data using a model based on the negative binomial distribution. The resulting *P* values were adjusted using Benjamini and Hochberg’s approach for controlling the false discovery rate and were named *P*_adj_. Coral genes with *P*_adj_ < 0.001 and |log2(FoldChange)| ≥ 2 and 10 as determined by DESeq2 were assigned as differentially expressed. Venn diagrams were drawn using the VennDiagram R package (1.6.20) and GO classification bar charts were drawn using the ggplot2 R package (3.3.5) [64].

### Limitations

This study offers insights by integrating micro-CT imaging, SEM-EDS, and RNA-seq, revealing unique coral growth patterns and molecular mechanisms under low-pH conditions. It identifies resilient and vulnerable species, which is crucial for conservation. However, the study on coral responses to ocean acidification has several limitations that should be considered when interpreting the results. These include the following:

1. Absence of direct measurements: The study did not measure in vivo pH or calcium flux directly, relying instead on RNA-seq and micro-CT imaging to infer coral responses. This could limit mechanistic understanding, but future research could use microelectrode techniques for real-time data.
2. Simplified pH conditions: The experiment simulated pH levels of 7.6 to 7.8 with day–night fluctuations, which may not capture the full complexity of natural pH variations, such as seasonal changes and biological interactions. This simplification helps isolate acidification effects but reduces ecological relevance, suggesting a need for more realistic pH studies in the future.
3. Limited species coverage: Only 4 coral species (*A. muricata*, *M. capricornis*, *M. foliosa*, and *P. damicornis*) were studied, representing different genera but not the full diversity. This restricts the generalizability of findings, and expanding to more species could provide a broader understanding.
4. Controlled lab conditions: The experiment maintained constant temperature and salinity, isolating pH effects but not reflecting natural multiple stressors. Future studies should include these to assess combined impacts on coral health.
5. Short-term duration: The 30-day experiment provides insights into acute responses but not long-term adaptation or evolution. Long-term studies are needed to explore transgenerational plasticity, genetic variation, and microbial community shifts.
6. Unused techniques: Techniques like x-ray diffraction were not used, which could offer additional insights into skeletal mineralogical properties and mechanical strength. Future research could adopt these for deeper analysis.

## Ethical Approval

This study did not involve experiments on cephalopods or higher animals. All coral sample collection and processing were performed according to the local laws governing the welfare of invertebrate animals.

## Data Availability

Data produced in this study are available at the Sequence Read Archive (SRA) (https://www.ncbi.nlm.nih.gov/sra/) under accession numbers SAMN16237127-SAMN16237130, SAMN16456055-SAMN16456058, SAMN16365802-SAMN16365813, and SAMN16237439-SAMN16237462. Details can be checked in the Supplementary File.
