# Supplementary material for: Skeleton-Forming Responses of Reef-Building Corals under Ocean Acidification
Source: Research (Wash D C). 2025 Jun 11;8:0736. doi: 10.34133/research.0736 (PMC12152308; doi:10.34133/research.0736)
Supplement: Supplementary 1 — Figs. S1 to S30 Tables S1 to S5 [file research.0736.f1.docx]

**Supplementary information of:**

Skeleton-forming responses of reef-building corals under ocean acidification

Yixin Li^1,2,*^, Hongwei Zhao^3^, Yunpeng Zhao^1^, Xin Liao^4^, J.-Y. Chen^5^, Yanping Qin^6,7*^, Zuhong Lu^2,*^, Yuehuan Zhang^6,7*^, Chunpeng He^2,*^

^1^State Key Laboratory of Coastal and Offshore Engineering, Dalian University of Technology, Dalian 116024, China.

^2^State Key Laboratory of Bioelectronics, School of Biological Science and Medical Engineering, Southeast University, Nanjing 210096, China.

^3^State Key Laboratory of Marine Resources Utilization in South China Sea, Hainan University, Haikou 570228, China.

^4^Guangxi Key Lab of Mangrove Conservation and Utilization, Guangxi Mangrove Research Center, Guangxi Academy of Sciences, Beihai 536000, China.

^5^Nanjing Institute of Geology and Palaeontology, Chinese Academy of Sciences, 39 East Beijing Road, Nanjing 210008, China.

^6^Southern Marine Science and Engineering Guangdong Laboratory (GuangZhou), Guangzhou, 511458, China.

^7^South China Sea Institute of Oceanology, Chinese Academy of Science, Guangzhou 510301, China.

***Corresponding authors:** Yixin Li, Yanping Qin, Zuhong Lu, Yuehuan Zhang, Chunpeng He.

**Part 1 | Supplementary Figures**

**Part 2 | Supplementary Tables**

**Part 3 | Data Availability Statement**

**Part 1 | Supplementary Figures**


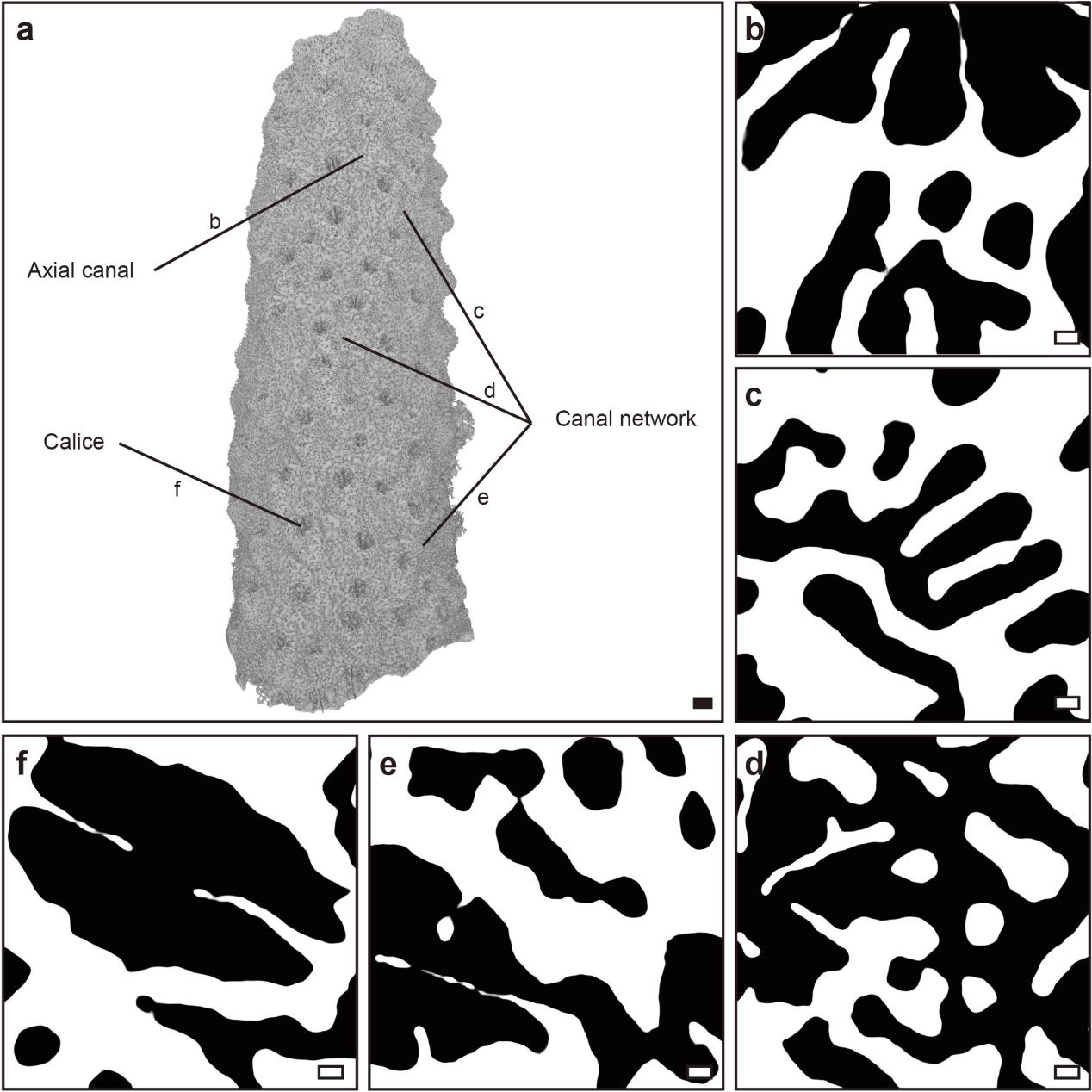


**Supplementary Figure 1 | Micro-CT reconstructions of *A. muricata* on Day 0.** Reconstruction of the polyp-canal system and skeletons in an *A. muricata* sample at Day 0, showing the detailed structural information for small spatial scales prior to lower pH stress exposure as a control. Scale bars: a) 1 mm; b-f) 0.1 mm.


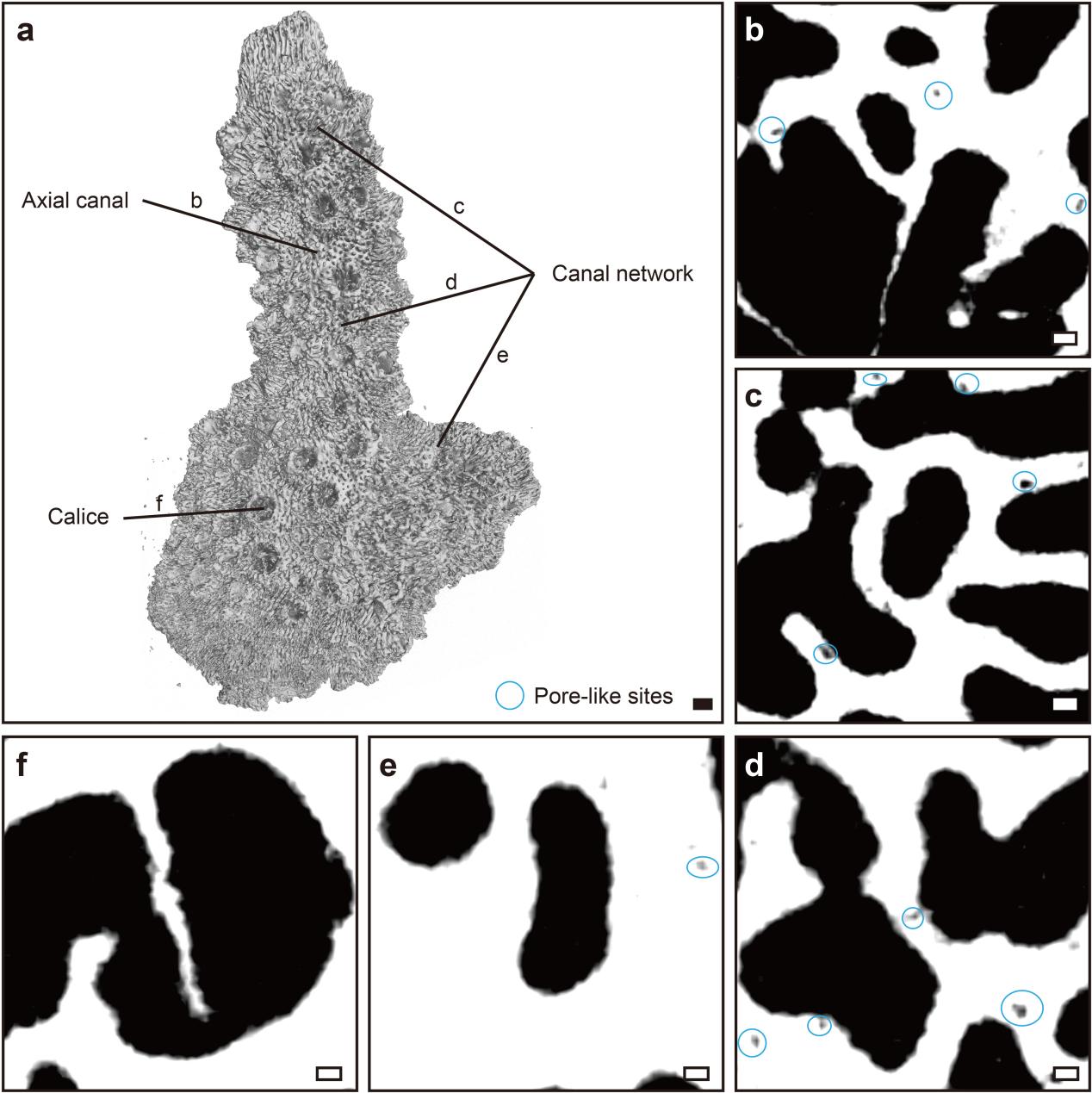


**Supplementary Figure 2 | Micro-CT reconstructions of *A. muricata* on Day 3.** Occurrence of acidic damage sites (pore-like sites) in *A. muricata* at Day 3, appearing initially in skeletons near the canal system. Scale bars: a) 1 mm; b-f) 0.1 mm.


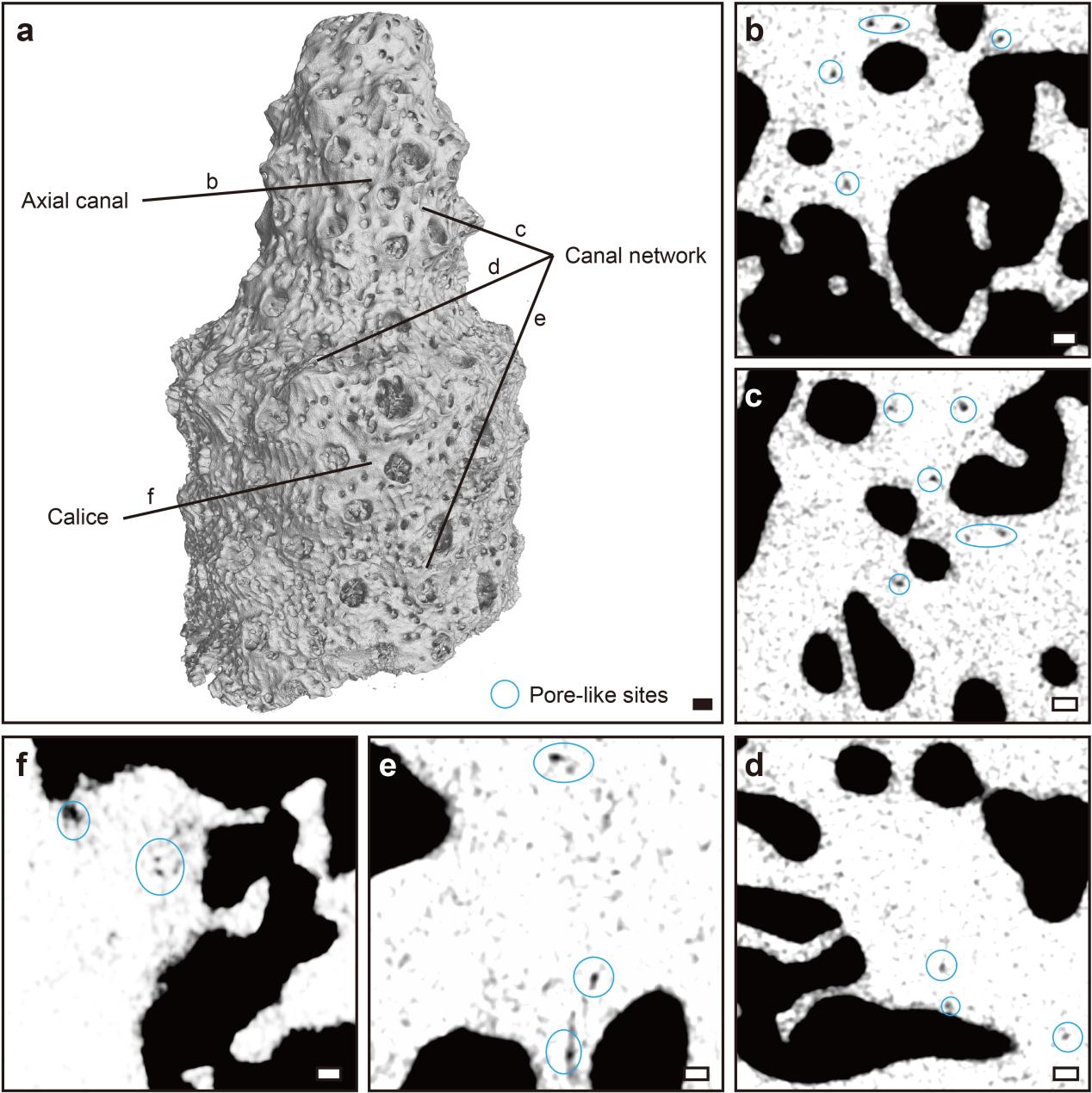


**Supplementary Figure 3 | Micro-CT reconstructions of *A. muricata* on Day 6.** Pore-like sites in axial skeletons and corallites near coral polyps of *A. muricata* at Day 6, with internal corrosion and surface erosion visible. Scale bars: a) 1 mm; b-f) 0.1 mm.


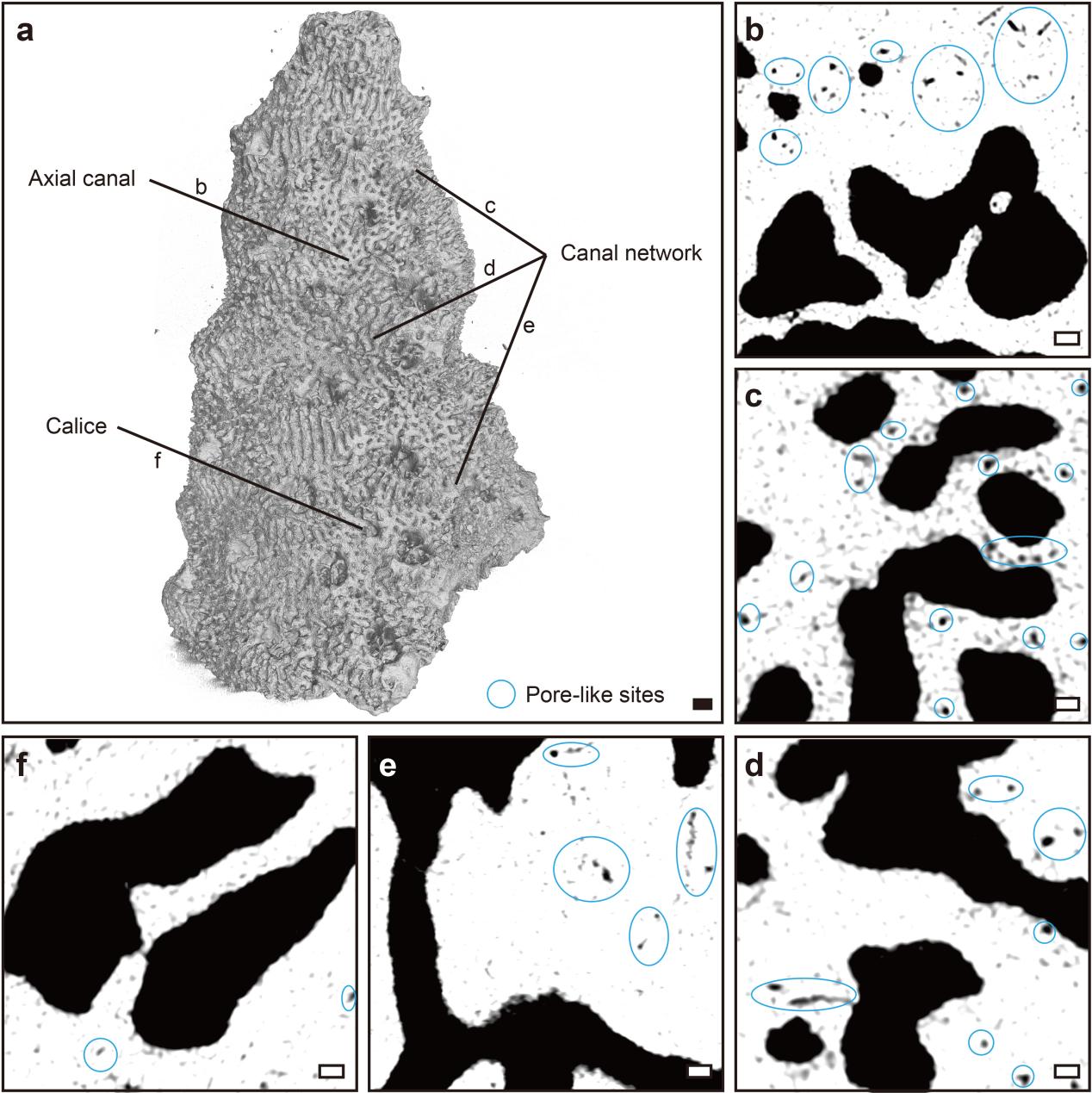


**Supplementary Figure 4 | Micro-CT reconstructions of *A. muricata* on Day 9.** Continued internal corrosion and surface erosion of *A. muricata* skeletons at Day 9, expanding from the initial acidic damage sites. Scale bars: a) 1 mm; b-f) 0.1 mm.


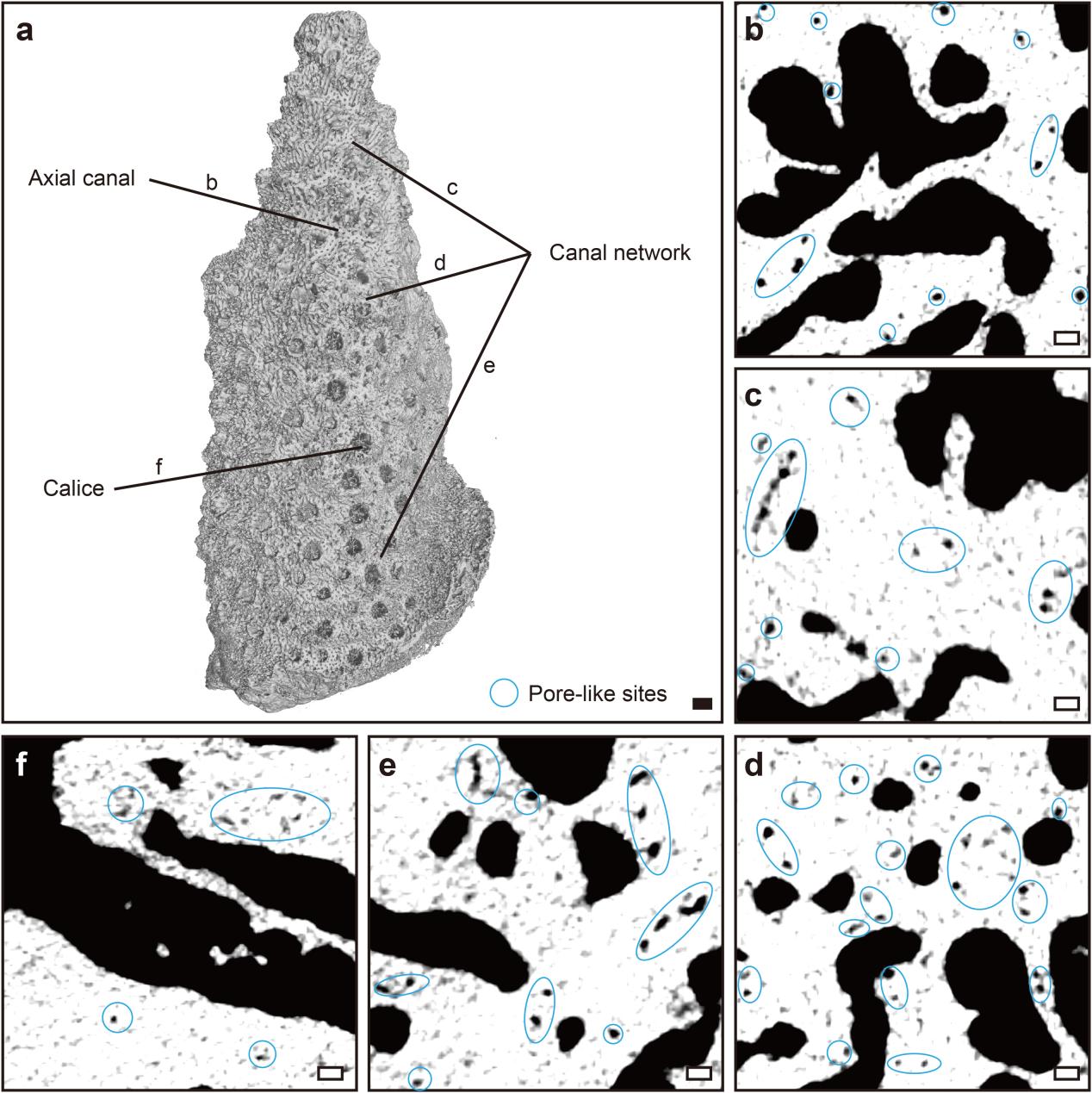


**Supplementary Figure 5 | Micro-CT reconstructions of *A. muricata* on Day 30.** Extensive surface erosion and merging of adjacent pore-like acid sites within the skeletal structure of *A. muricata* at Day 30. Scale bars: a) 1 mm; b-f) 0.1 mm.


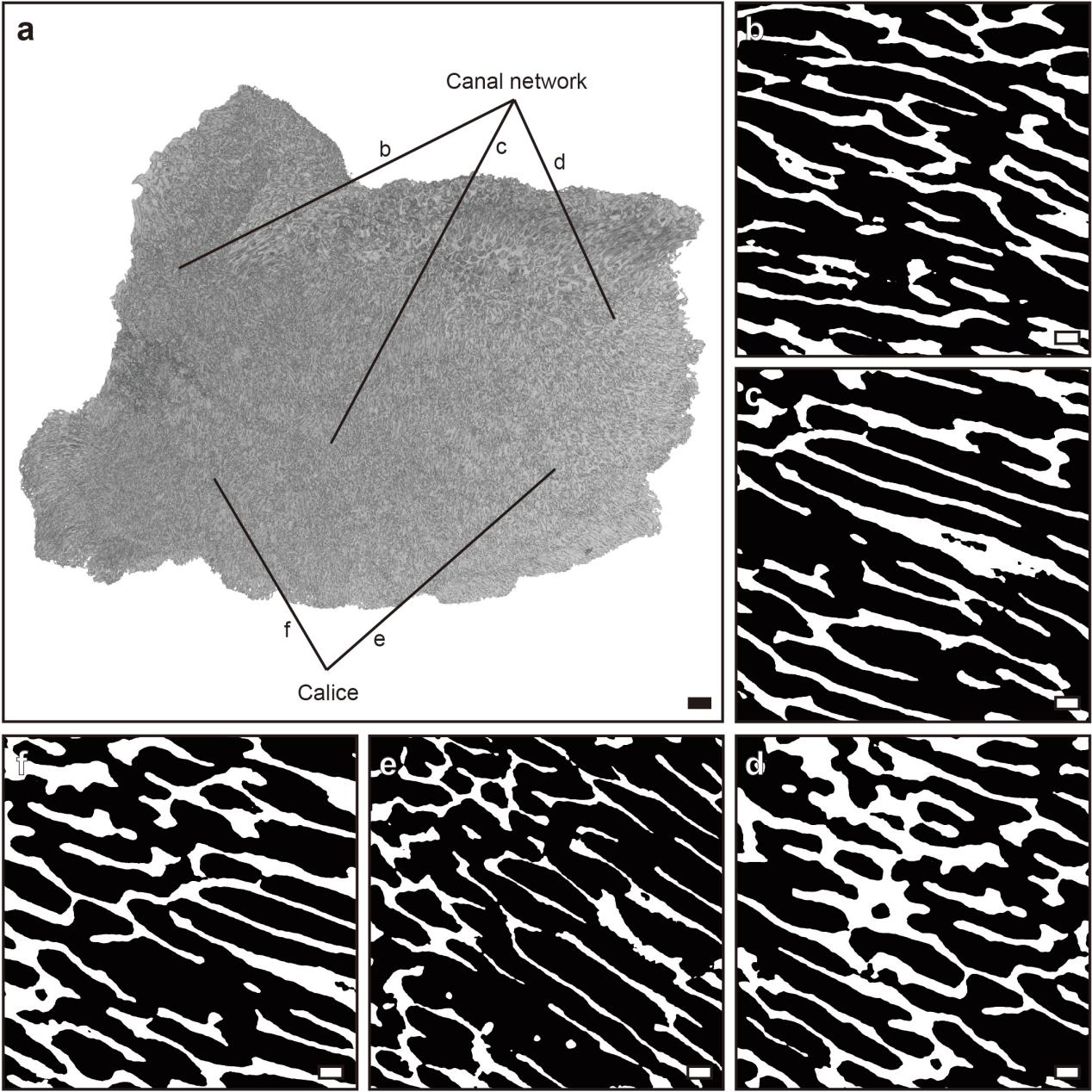


**Supplementary Figure 6 | Micro-CT reconstructions of *M. capricornis* on Day 0.** Coral samples of *M. capricornis* at Day 0, showing intact polyp-canal systems before the onset of lower pH. Scale bars: a) 1 mm; b-d) 0.1 mm; e,f) 0.2mm.


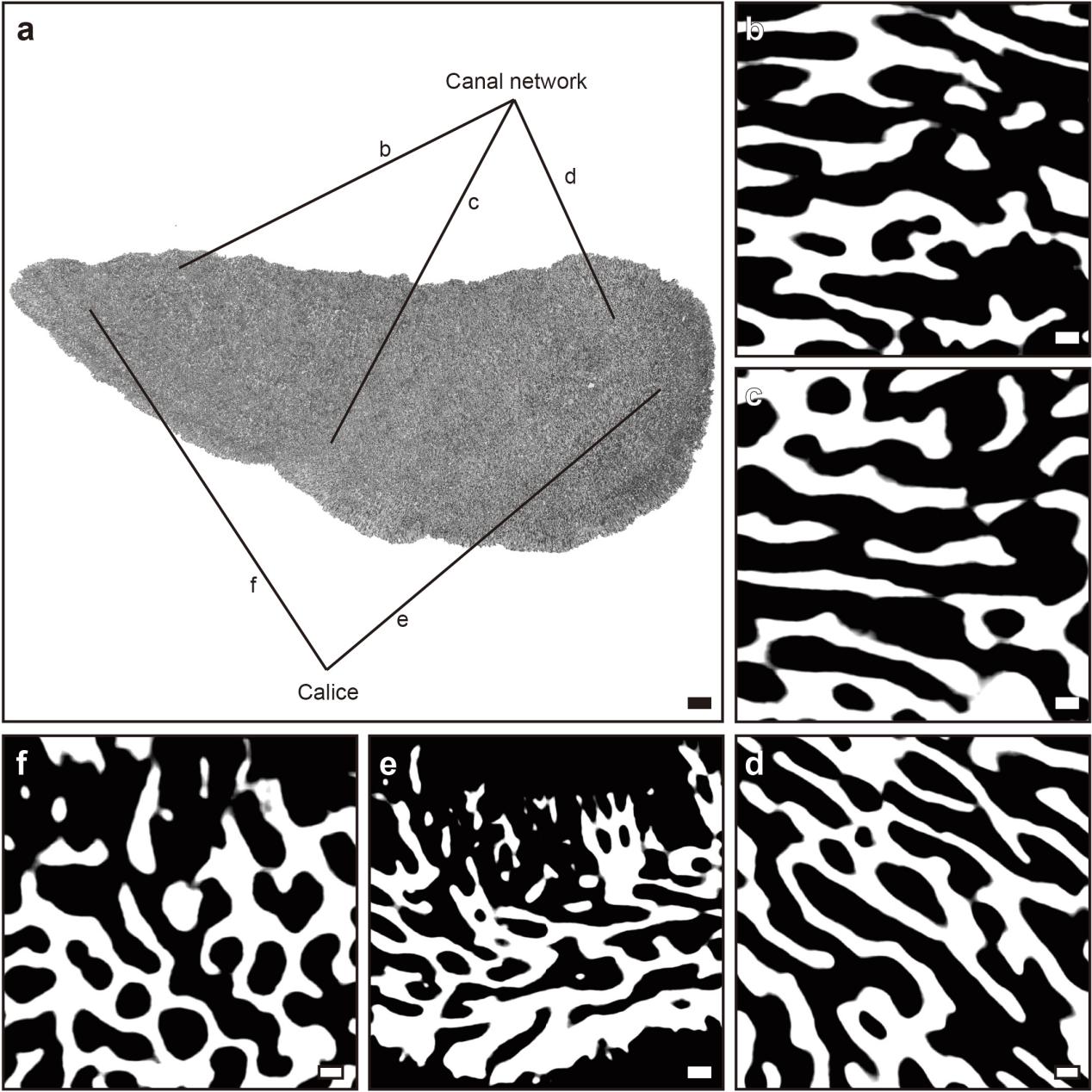


**Supplementary Figure 7 | Micro-CT reconstructions of *M. capricornis* on Day 3.** In the coral samples of *M. capricornis* at Day 3, the structure of the skeleton and canal system showed almost no changes, remaining similar to Day 0. Scale bars: a) 1 mm; b-d) 0.1 μm; e) 0.2 mm; f) 0.1 mm.


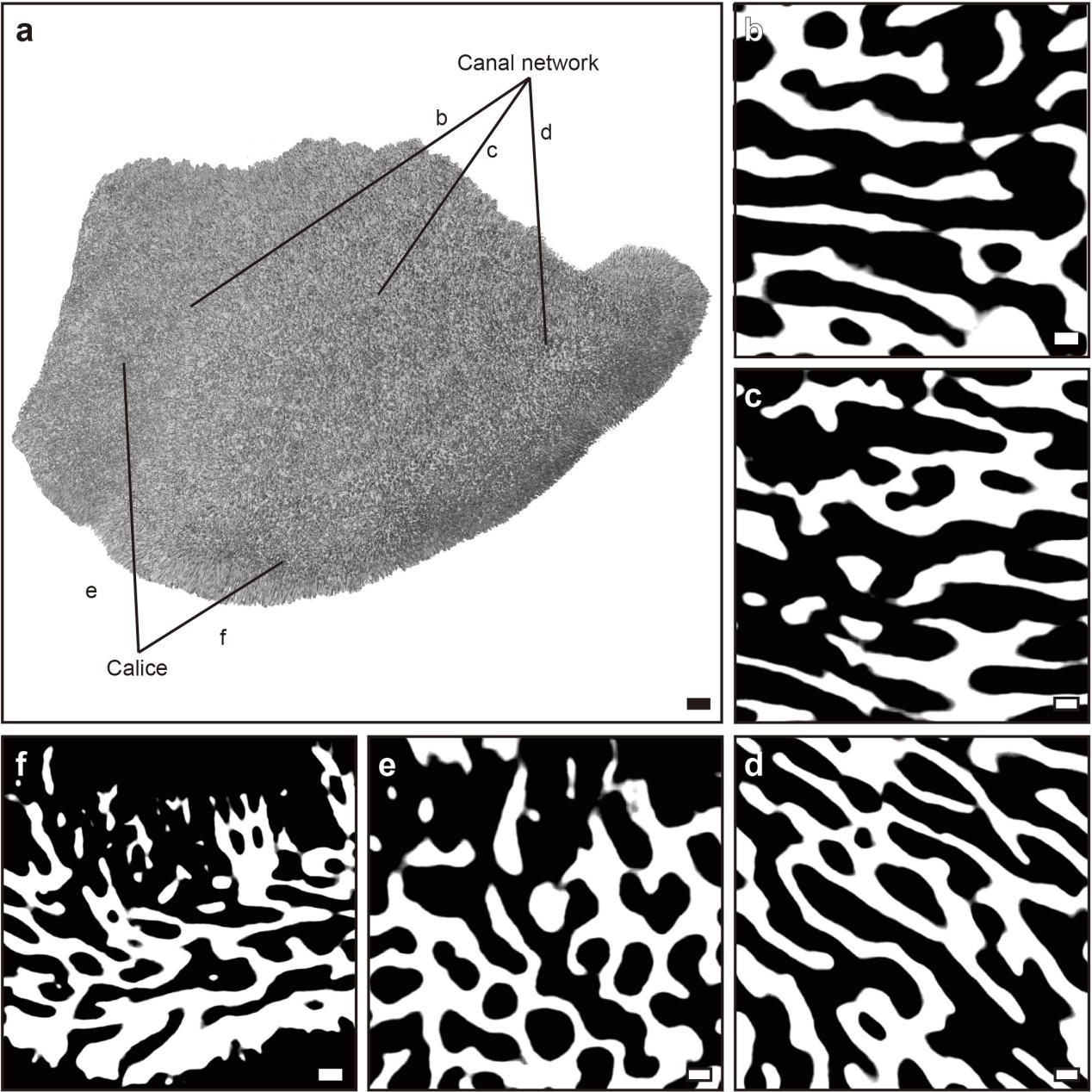


**Supplementary Figure 8 | Micro-CT reconstructions of *M. capricornis* on Day 6.** Emergence of acid corrosion areas on the surface of *M. capricornis* skeletons at Day 6, slightly increasing the volume of the canal system. Scale bars: a) 1 mm; b-e) 0.1 mm; f) 0.2 mm.


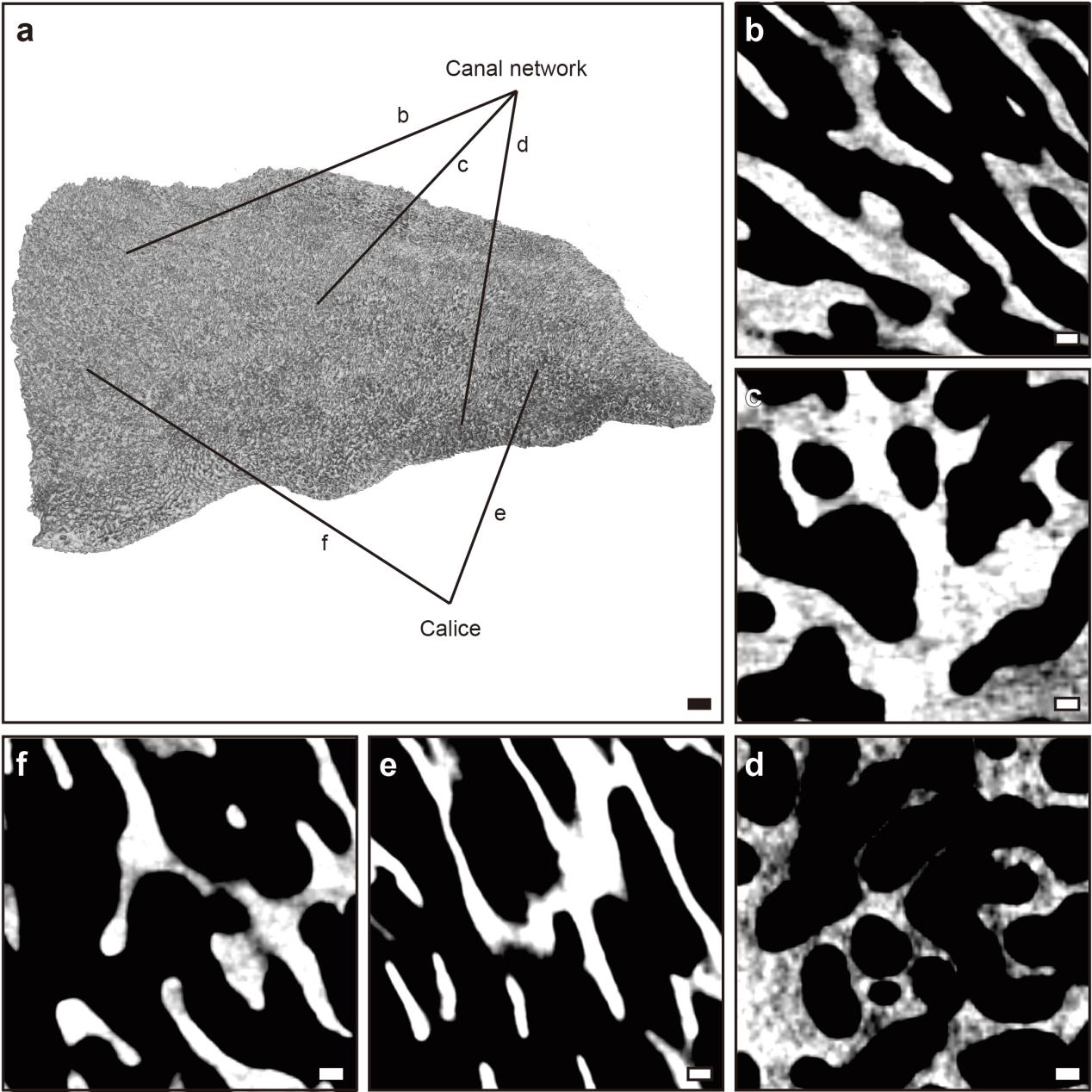


**Supplementary Figure 9 | Micro-CT reconstructions of *M. capricornis* on Day 9.** Expansion of corrosion areas across *M. capricornis* skeletons near the canal system at Day 9, with minimal damage over corallites. Scale bars: a) 1 mm; b-d) 0.1 mm; e,f) 0.2 mm.


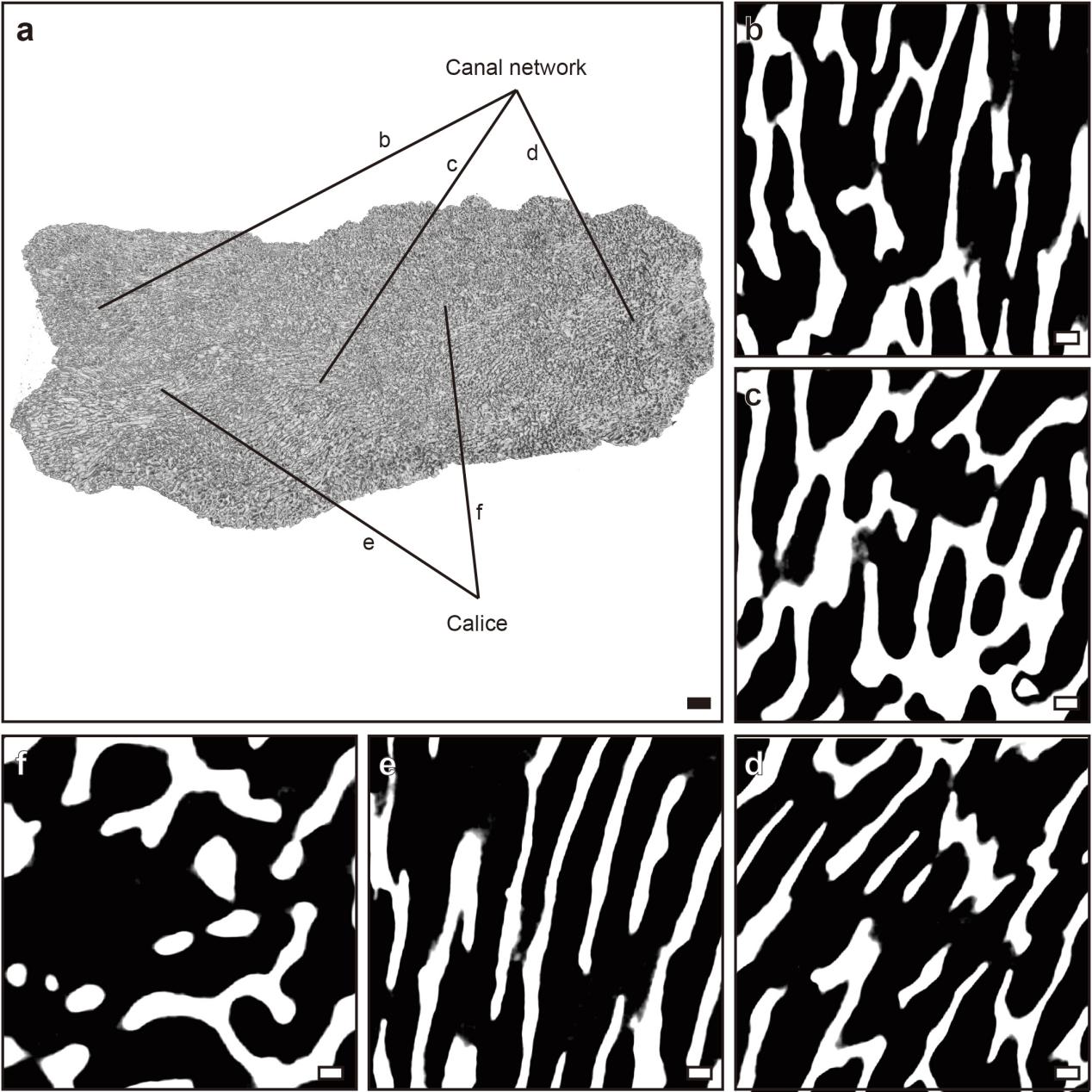


**Supplementary Figure 10 | Micro-CT reconstructions of *M. capricornis* on Day 30.** *M. capricornis* coral samples at Day 30, showing reduced corrosion areas shrank to almost invisible and structural similarity to Day 0, with a few reformed skeletons present. Scale bars: a) 1 mm; b-d) 0.1 mm; e) 0.2 mm; f) 0.1 mm.


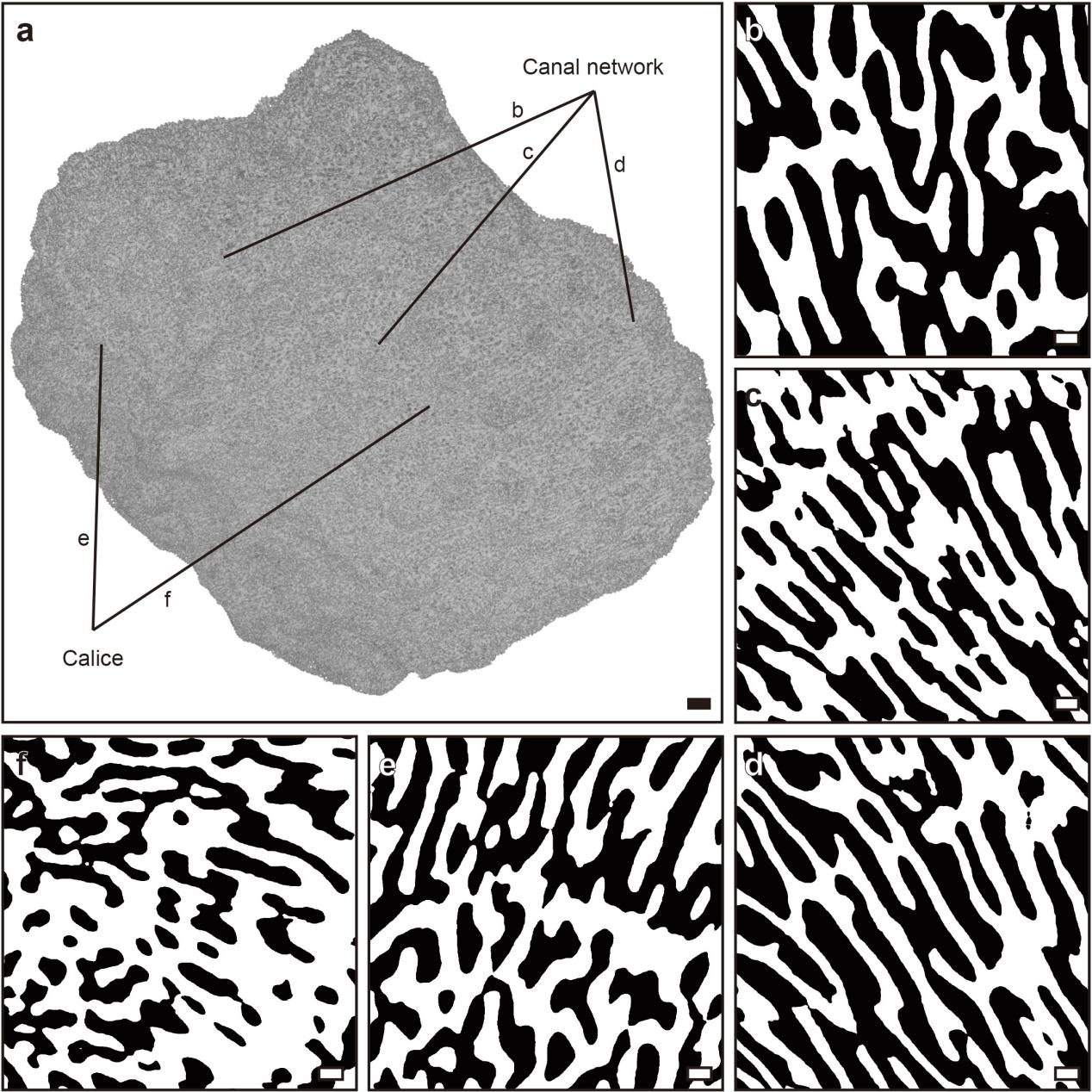


**Supplementary Figure 11 | Micro-CT reconstructions of *M. foliosa* on Day 0.** *M. foliosa* coral samples at Day 0, showing the intact skeletal and canal structures prior to lower pH stress. Scale bars: a) 1 mm; b-f) 0.1 mm.


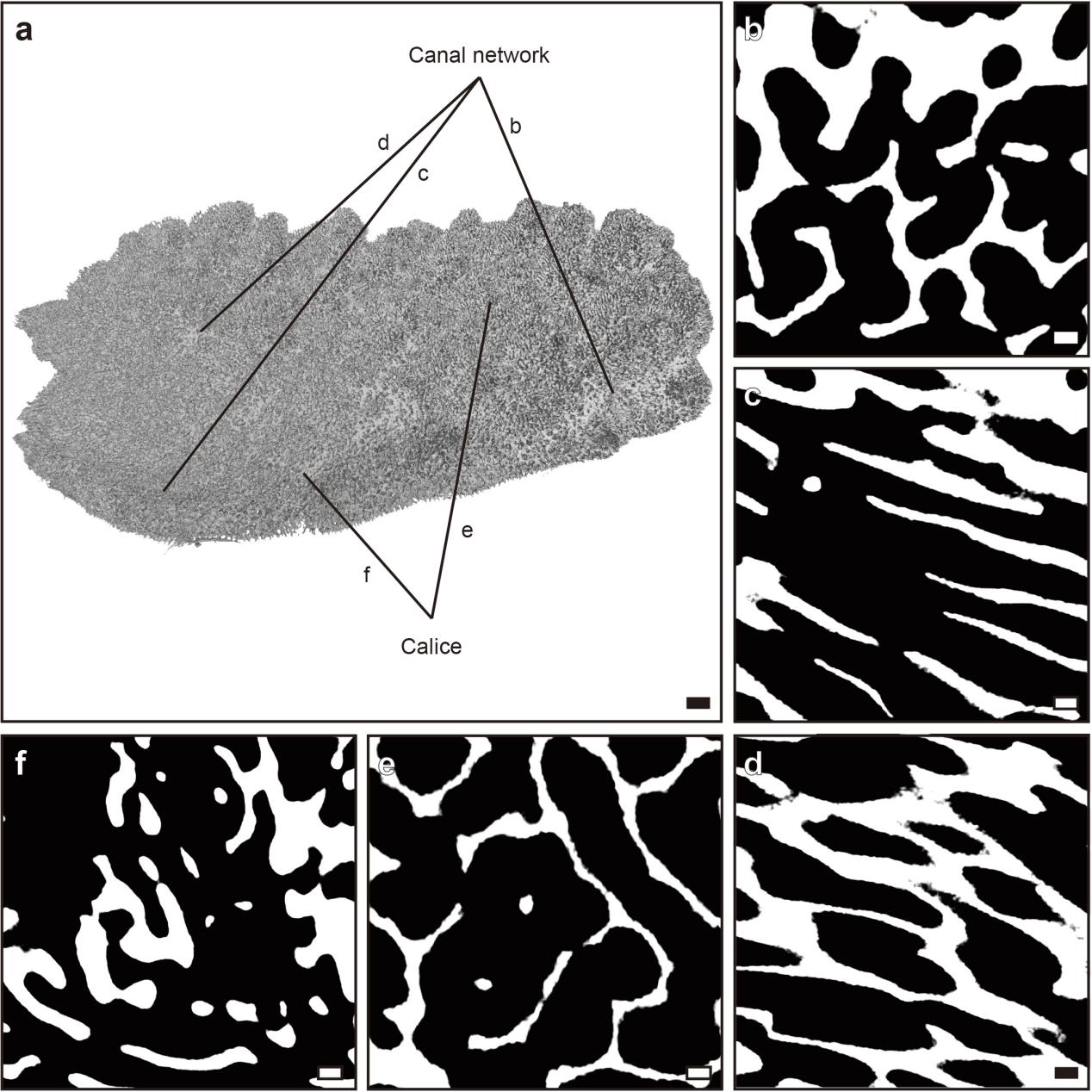


**Supplementary Figure 12 | Micro-CT reconstructions of *M. foliosa* on Day 3.** Acidic corrosion emerging on *M. foliosa* coral sample surfaces at Day 6, slightly expanding canal system volume. Scale bars: a) 1 mm; b-f) 0.1 mm.


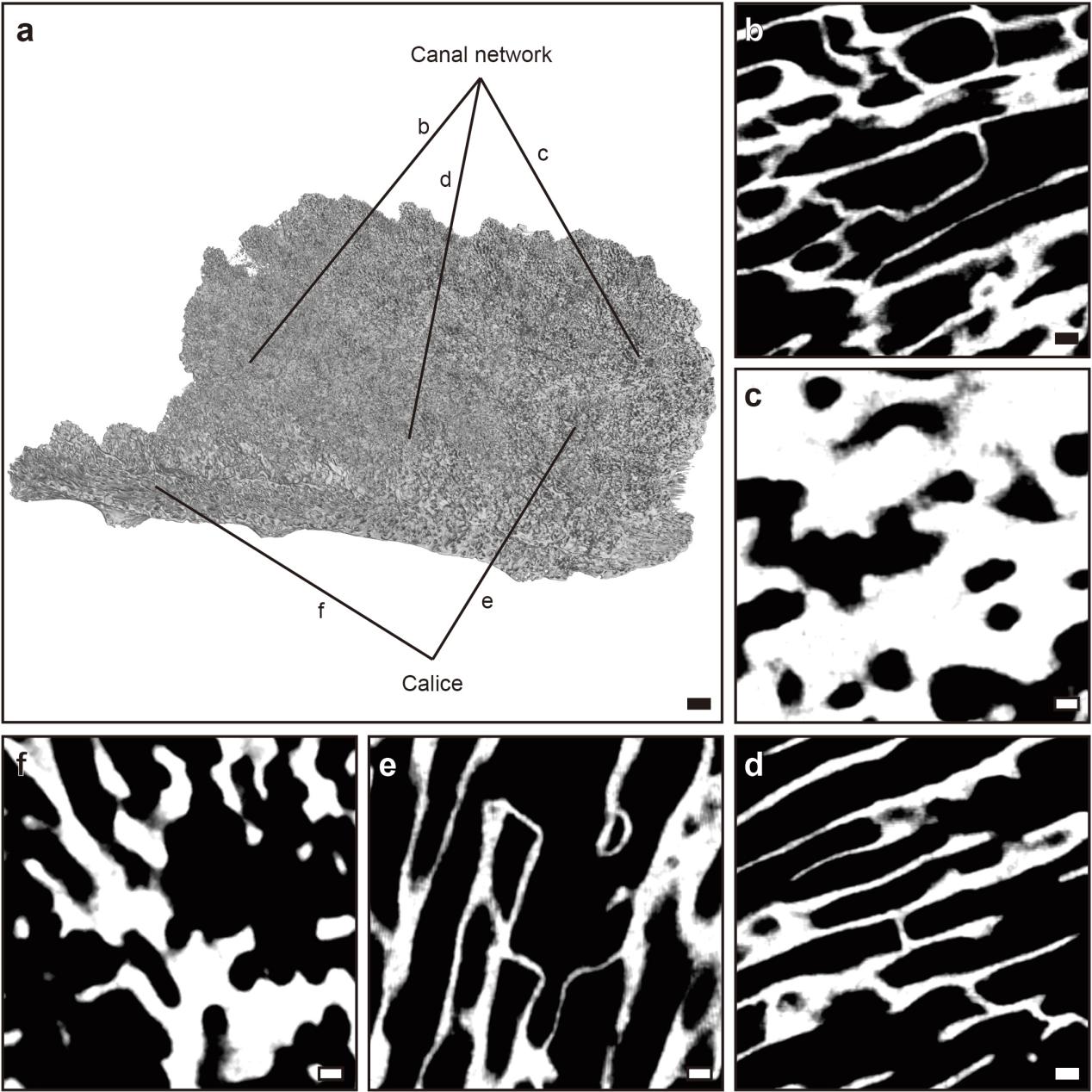


**Supplementary Figure 13 | Micro-CT reconstructions of *M. foliosa* on Day 6.** Corrosion areas on *M. foliosa* skeletons at Day 9, spreading near the canal system but showing fewer impacts on corallites. Scale bars: a) 1 mm; b-f) 0.1 mm.


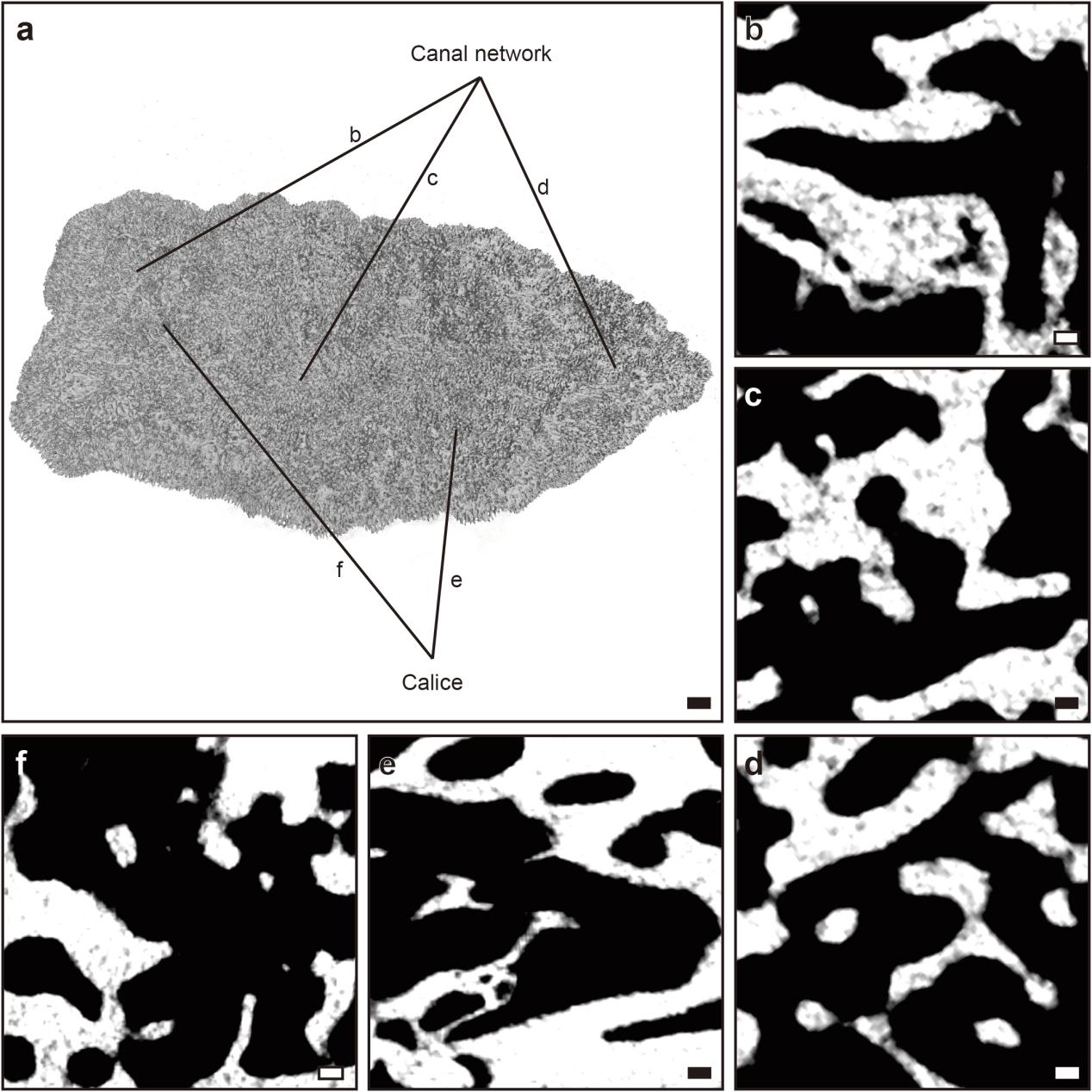


**Supplementary Figure 14 | Micro-CT reconstructions of *M. foliosa* on Day 9.** Spread of acid corrosion areas over the surface skeletons of *M. foliosa* at Day 9. Scale bars: a) 1 mm; b-f) 0.1 mm.


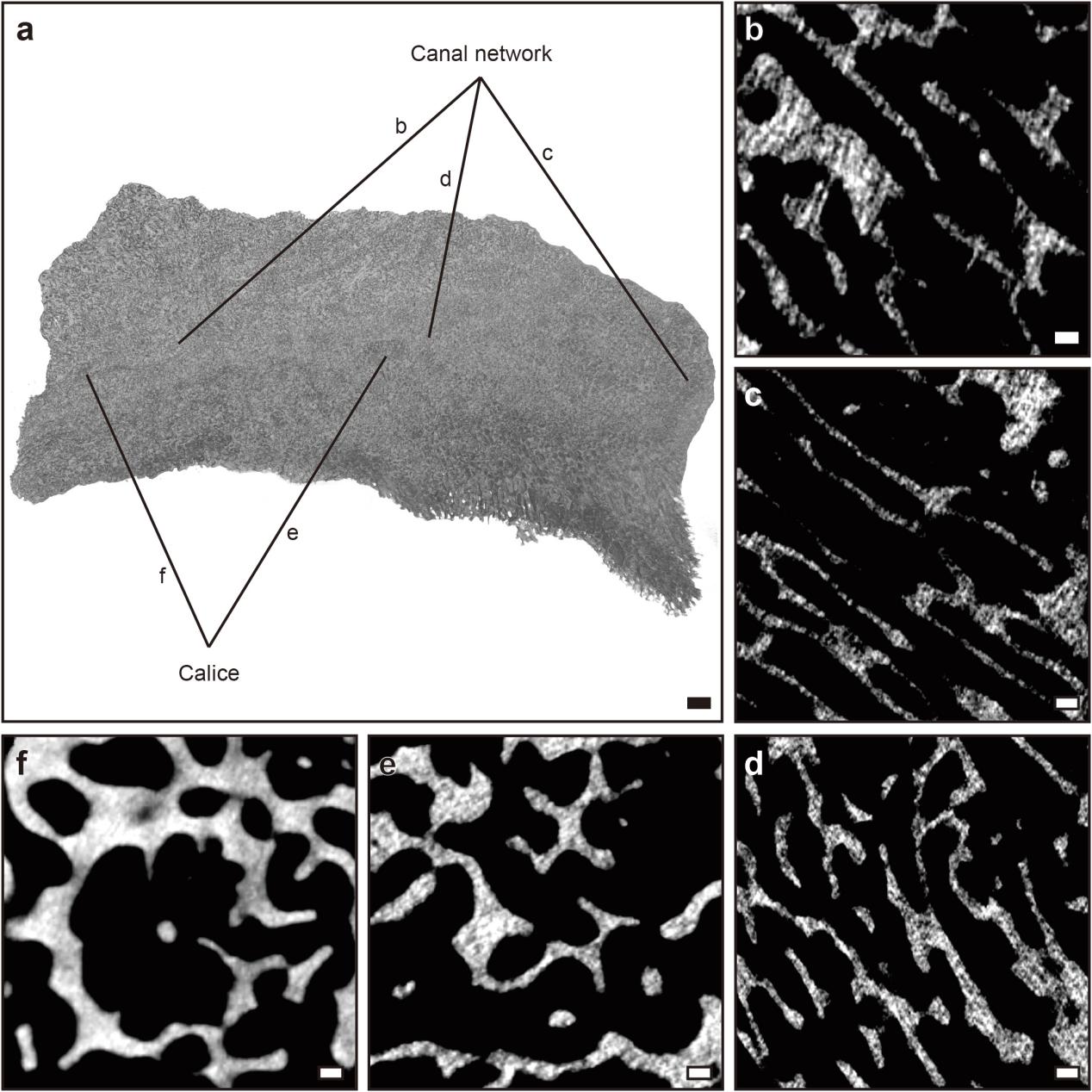


**Supplementary Figure 15 | Micro-CT reconstructions of *M. foliosa* on Day 30.** Extensive damage to the polyp-canal system in *M. foliosa* at Day 30, showing severe effects on coral growth. Scale bars: a) 1 mm; b-f) 0.1 mm.


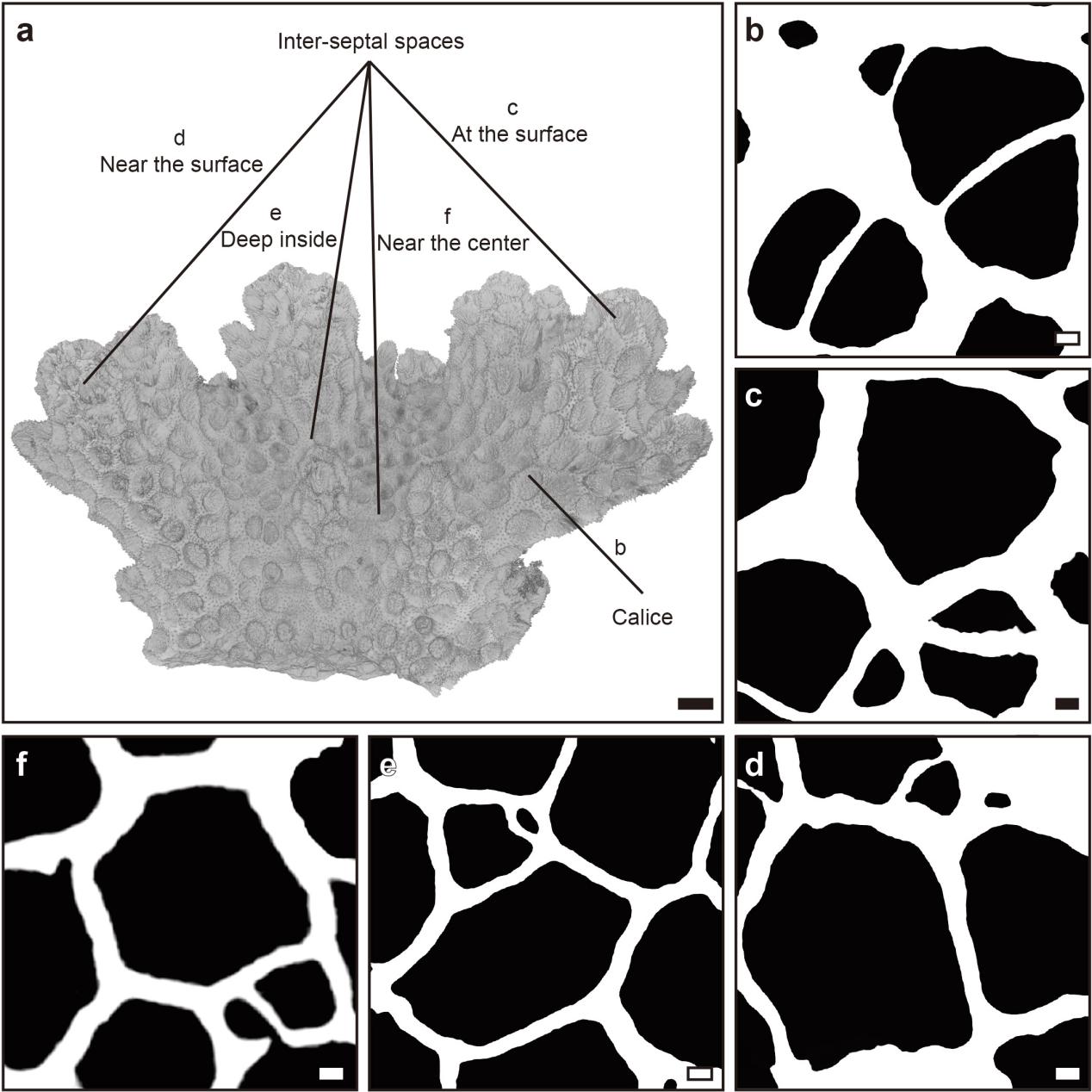


**Supplementary Figure 16 | Micro-CT reconstructions of *P. damicornis* on Day 0.** *P. damicornis* coral samples at Day 0, showing intact inter-septal spaces and skeletal structure prior to lower pH stress. Scale bars: a) 1 mm; b-f) 0.1 mm.


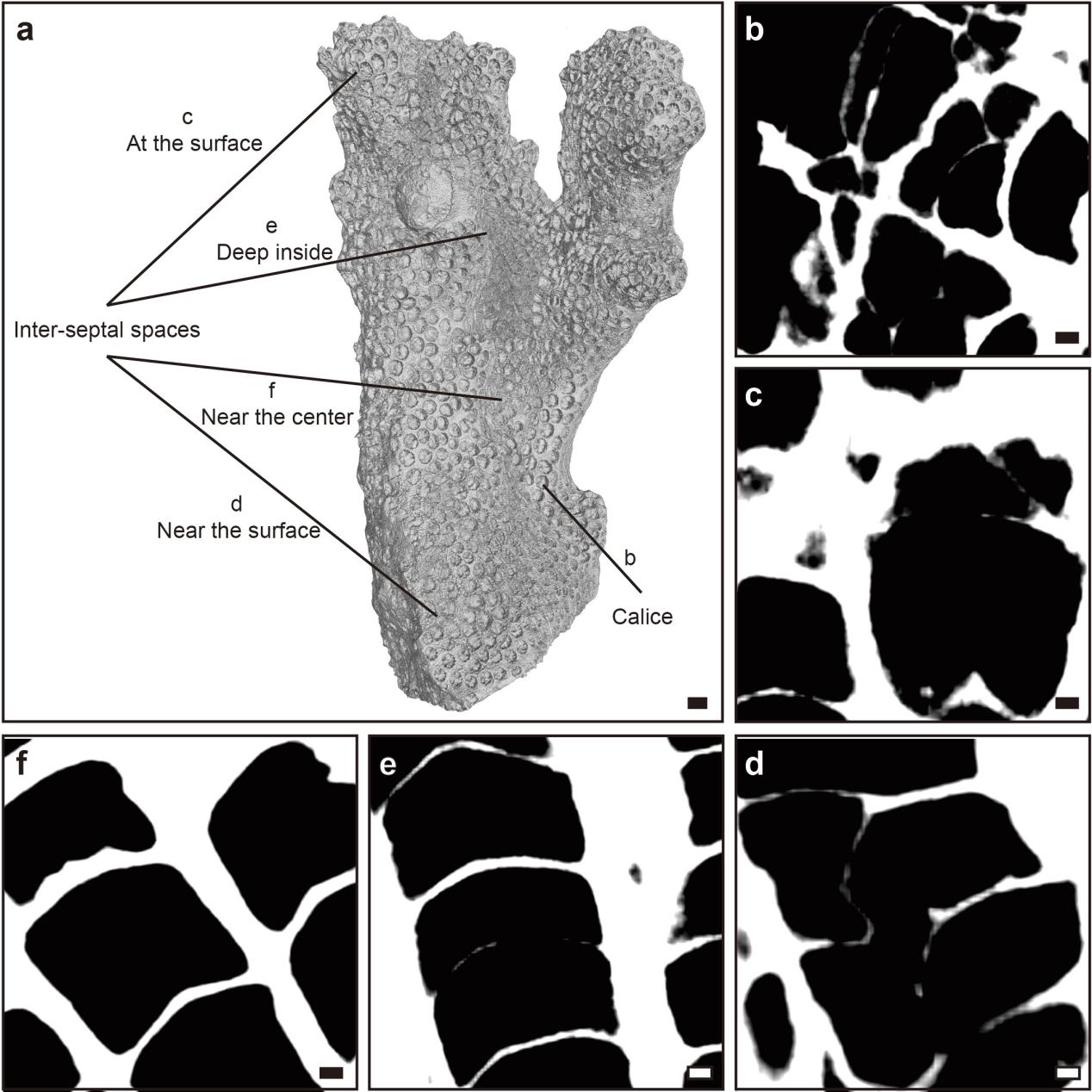


**Supplementary Figure 17 | Micro-CT reconstructions of *P. damicornis* on Day 3.** Initial acid corrosion observed in *P. damicornis* at Day 3, occurring on corallites and dissepiments near the colony surface. Scale bars: a) 1 mm; b-f) 0.1 mm.


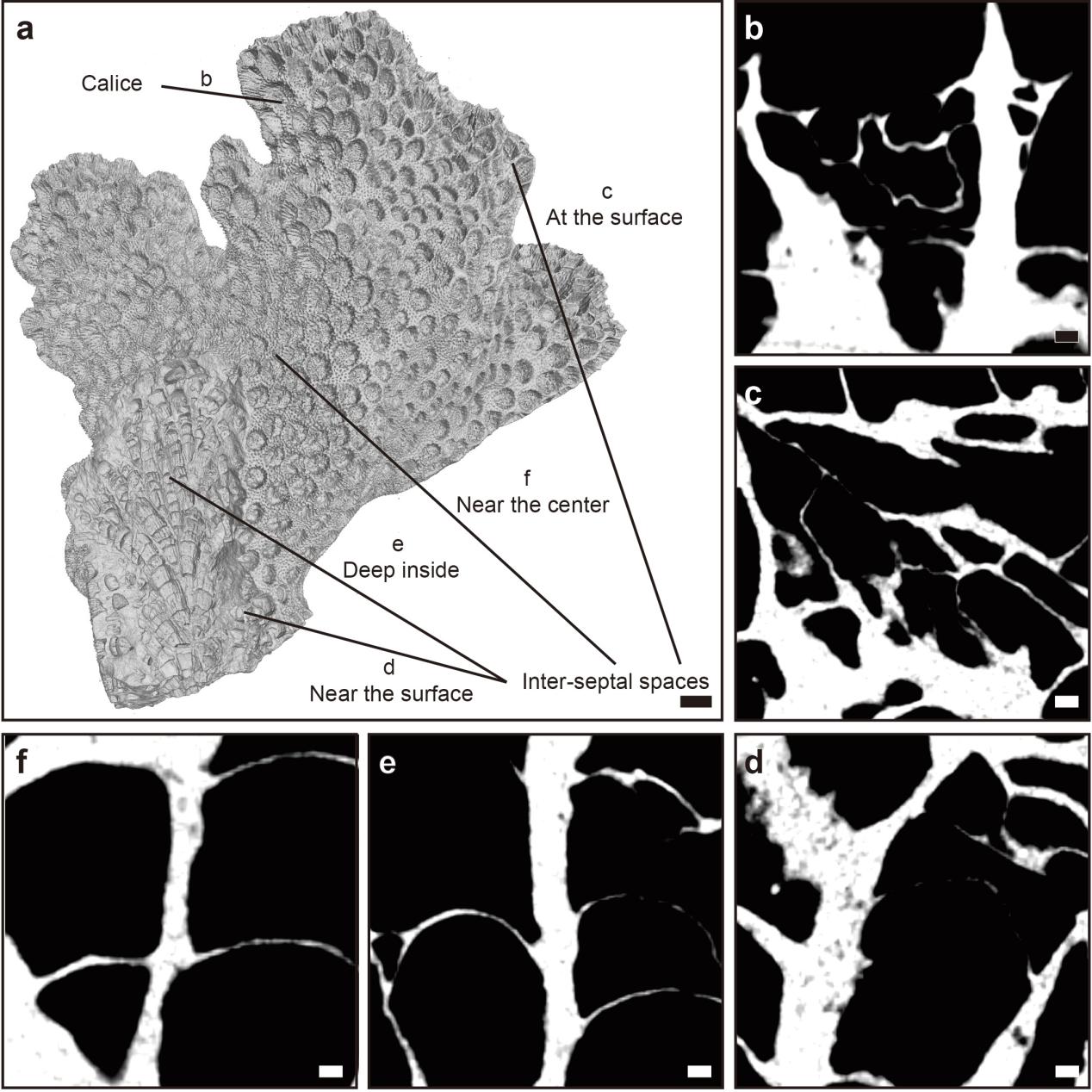


**Supplementary Figure 18 | Micro-CT reconstructions of *P. damicornis* on Day 6.** Spread of corrosion areas along coenosteums and coenosteums in *P. damicornis* at Day 6, with emerging reformed skeletons fragmenting the lumen in calices. Scale bars: a) 1 mm; b-f) 0.1 mm.


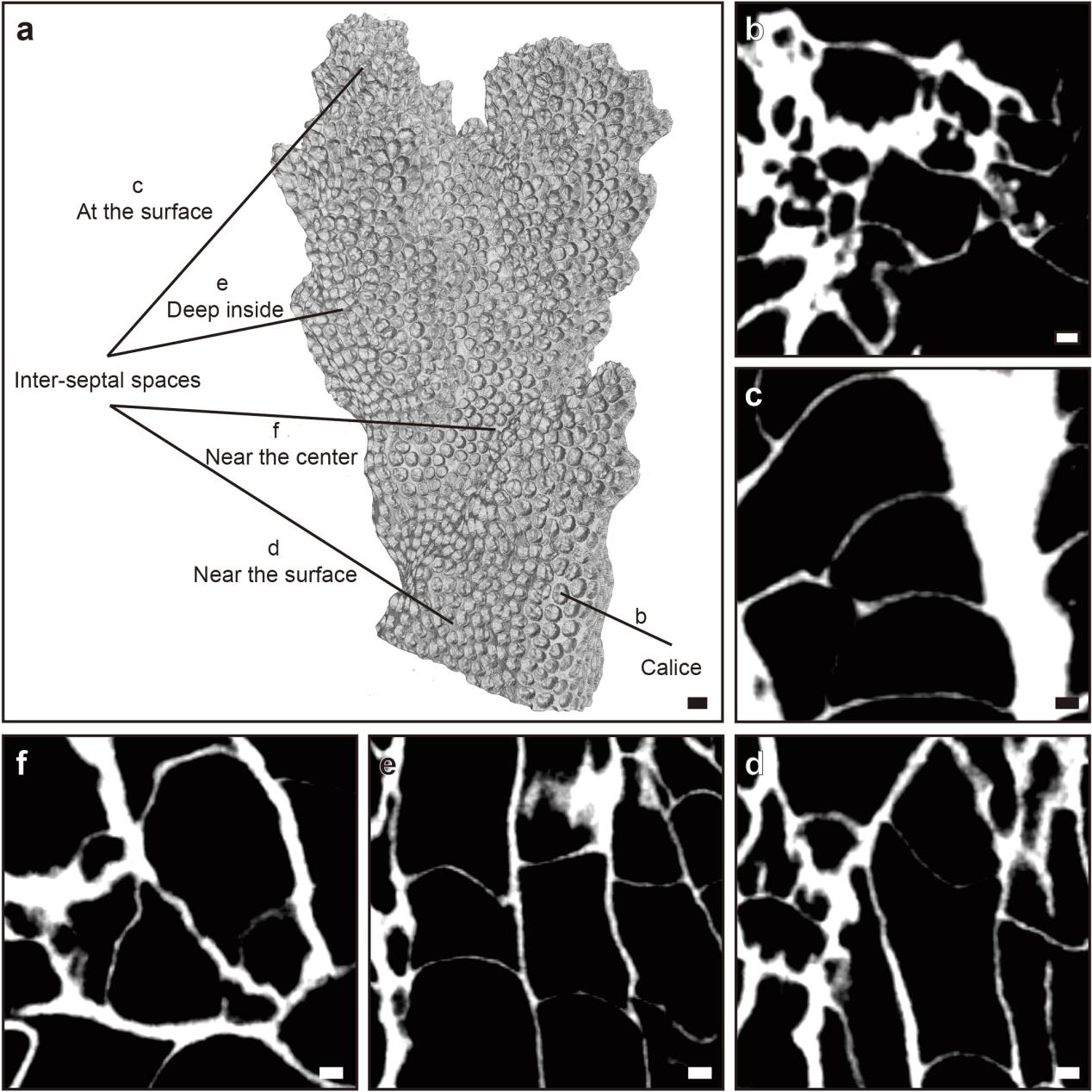


**Supplementary Figure 19 | Micro-CT reconstructions of *P. damicornis* on Day 9.** Severe damage to inter-septal spaces in *P. damicornis* coral samples at Day 9, showing merged spaces due to skeleton loss and breakage. Scale bars: a) 1 mm; b-f) 0.1 mm.


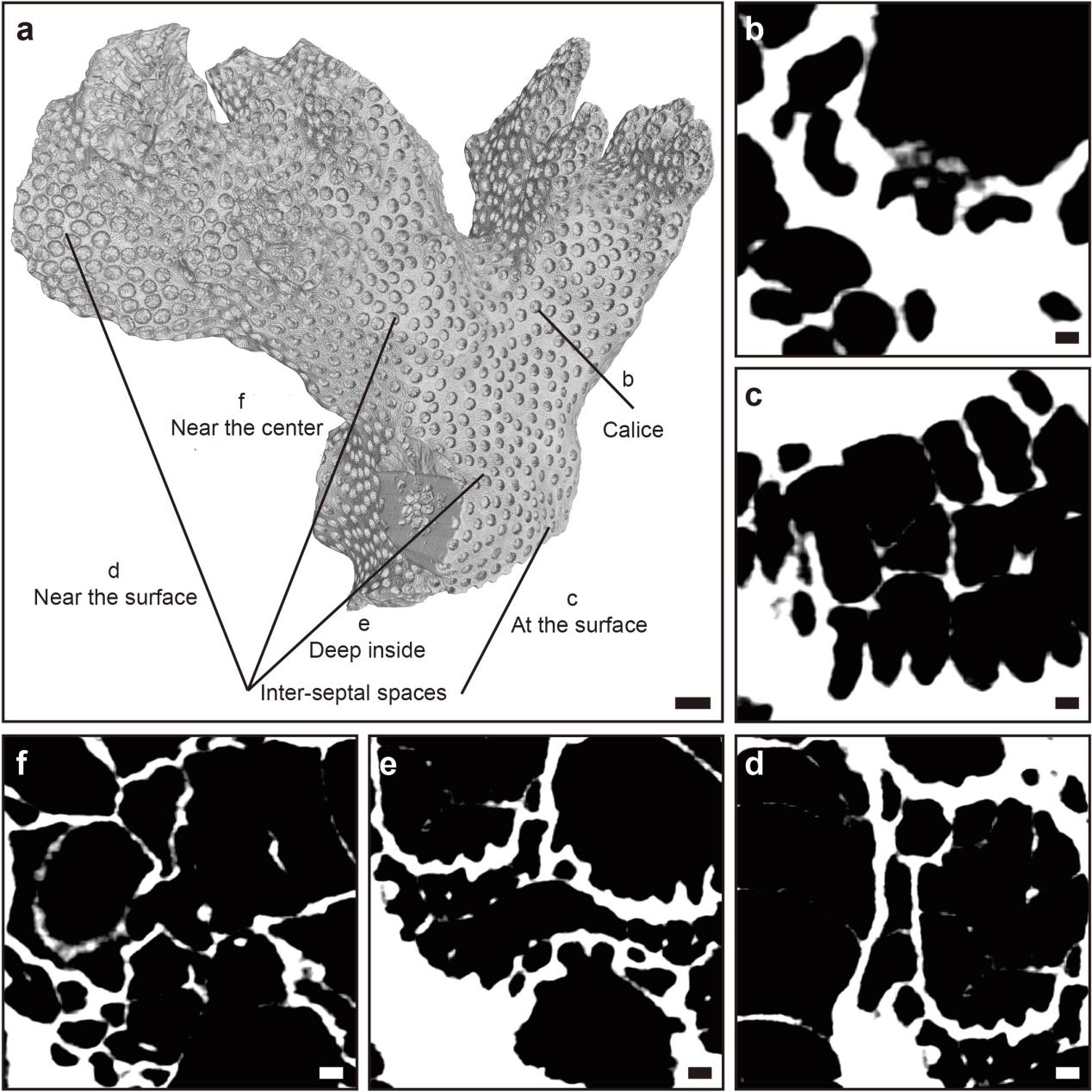


**Supplementary Figure 20 | Micro-CT reconstructions of *P. damicornis* on Day 30.** Reformed skeletons within calices of *P. damicornis* at Day 30, contributing to reduced living spaces for coral polyps. Scale bars: a) 1 mm; b-f) 0.1 mm.


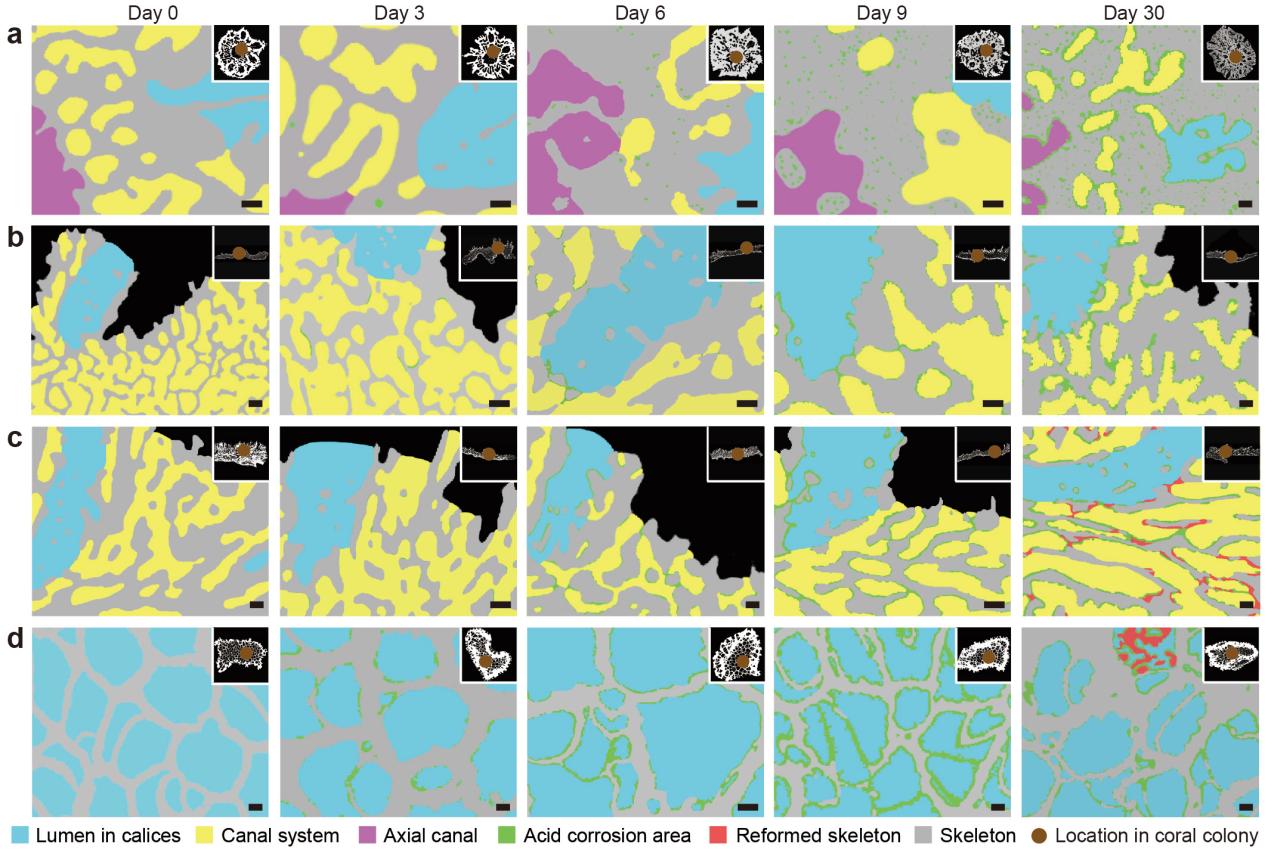


**Supplementary Figure 21 | Micro-CT reconstructions of skeletons and polyp-canal systems in coral samples from Day 0 to Day 30.** Micro-CT reconstructions visualized the 30-day growth pattern under lower pH in a) *A. muricata*, showing internal cavity formation with almost no visible damage on the surface, with corrosion pores expanding from initial internal damage sites and affecting the internal skeleton structure while maintaining the integrity of the polyp-canal system; b) *M. capricornis*, effects on the skeletons first appeared on Day 6 and peaked on Day 9, and showing reduced corrosion areas and reformed skeletons at Day 30, with a structure similar to the pre-acid stress condition. The polyp-canal system is largely maintained, and some reformed skeletons caused small gaps in the original canal system and lumen in calices; c) *M. foliosa*, showing continuous acid corrosion primarily affecting areas near the canal system. Corrosion near coral polyps is less pronounced, but the polyp-canal system suffers from serious damage that impacts coral growth; d) *P. damicornis*, showing progressively increasing corrosion damage till Day 30 to the inter-septal spaces and extensive corrosion areas covering internal skeletons. Some dissepiments and coenosteums are fractured, leading to merged inter-septal spaces and irregular skeletal structures newly formed within the calices reduced living areas for coral polyps. Scale bars: 0.1 mm.


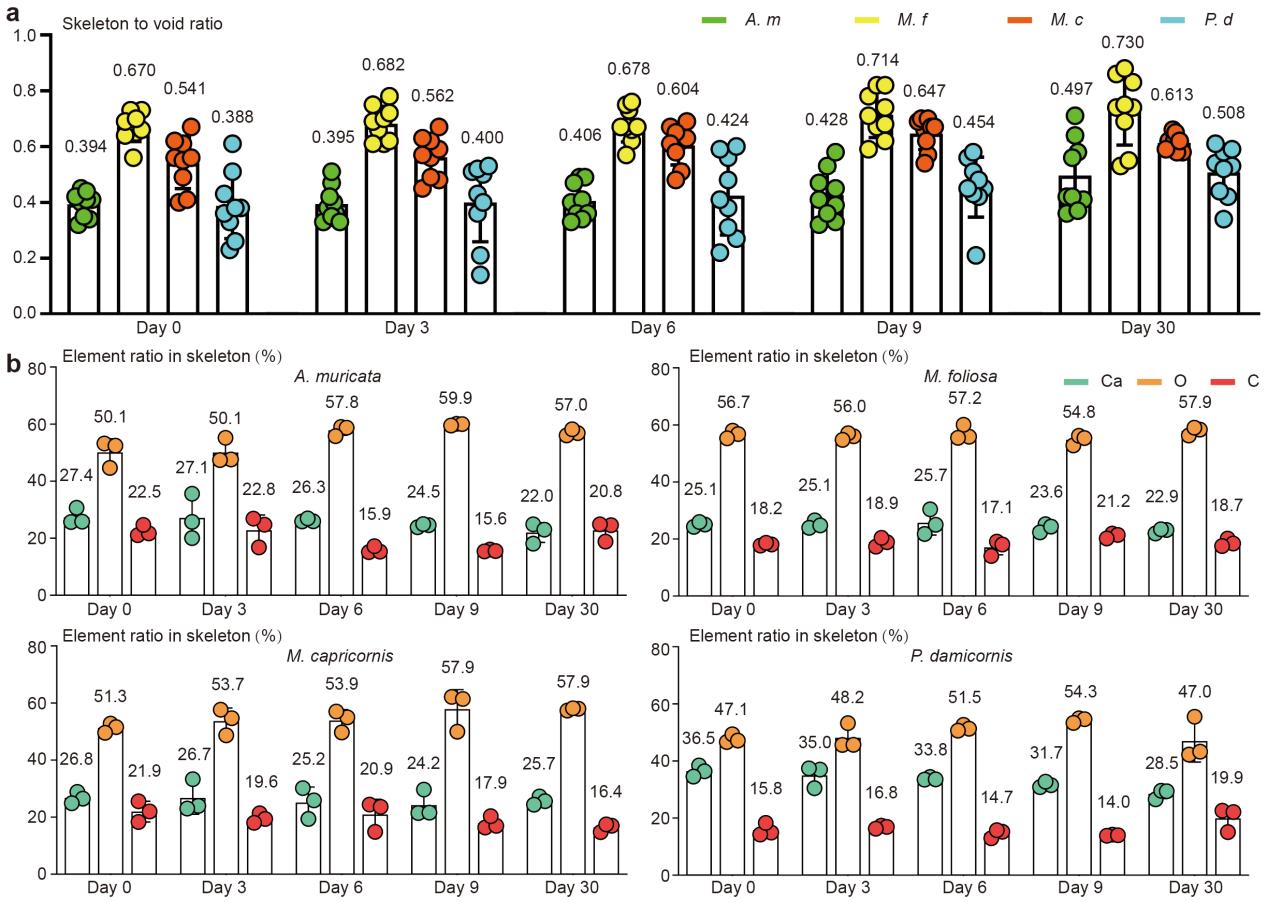


**Supplementary Figure 22 | Skeleton and element loss in coral samples from Day 0 to Day 30.** a) Skeleton to void ratio reveal the skeleton loss during acidic stress. b) Element changes of Ca, O, C reveal the affect of ocean acidification on coral skeletons.


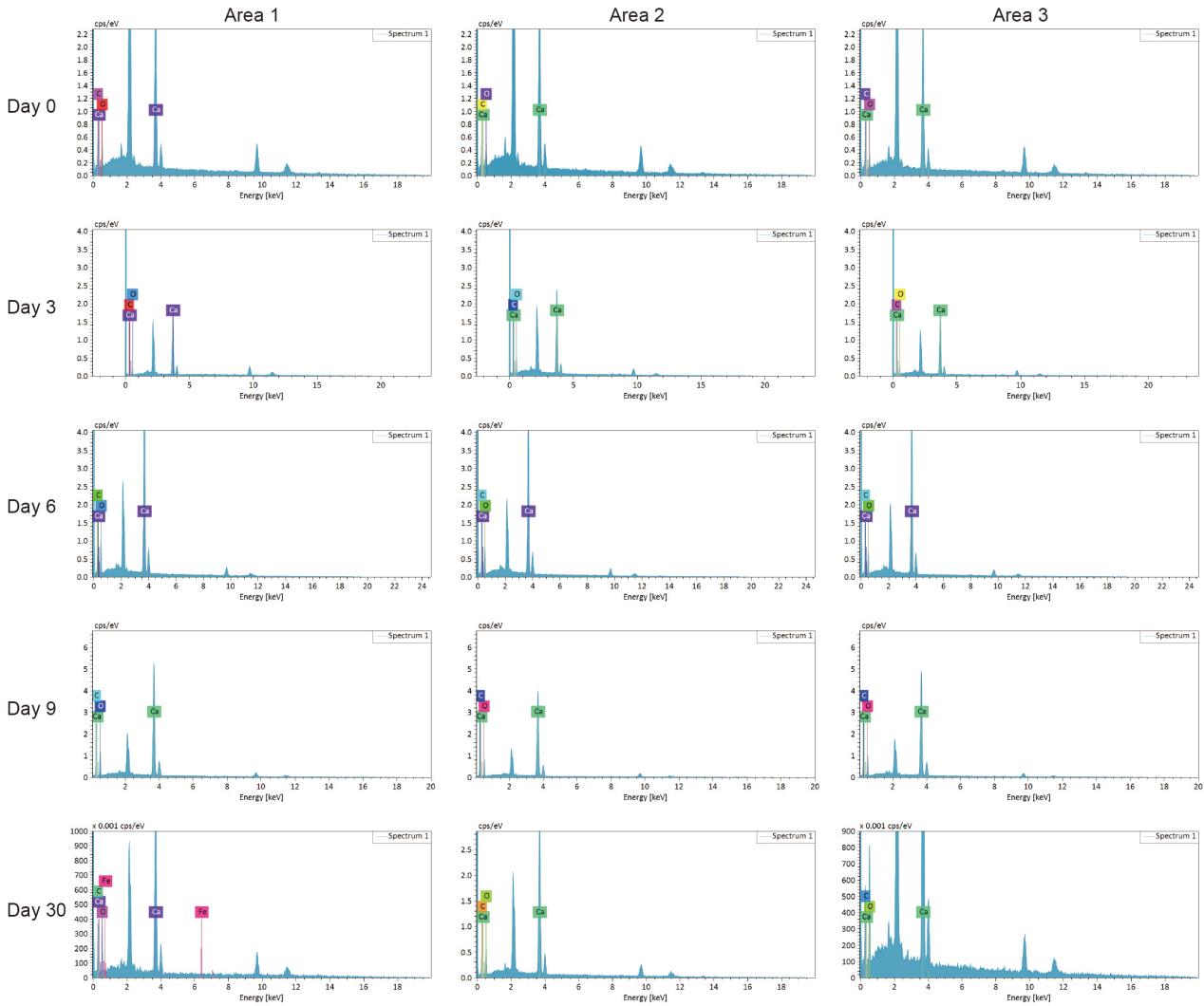


**Supplementary Figure 23 | EDS test of *A. muricata*.** The atomic ratio of Ca decreased between Days 0 and 30, with rates of decrease of 5.40% in *A. muricata*.


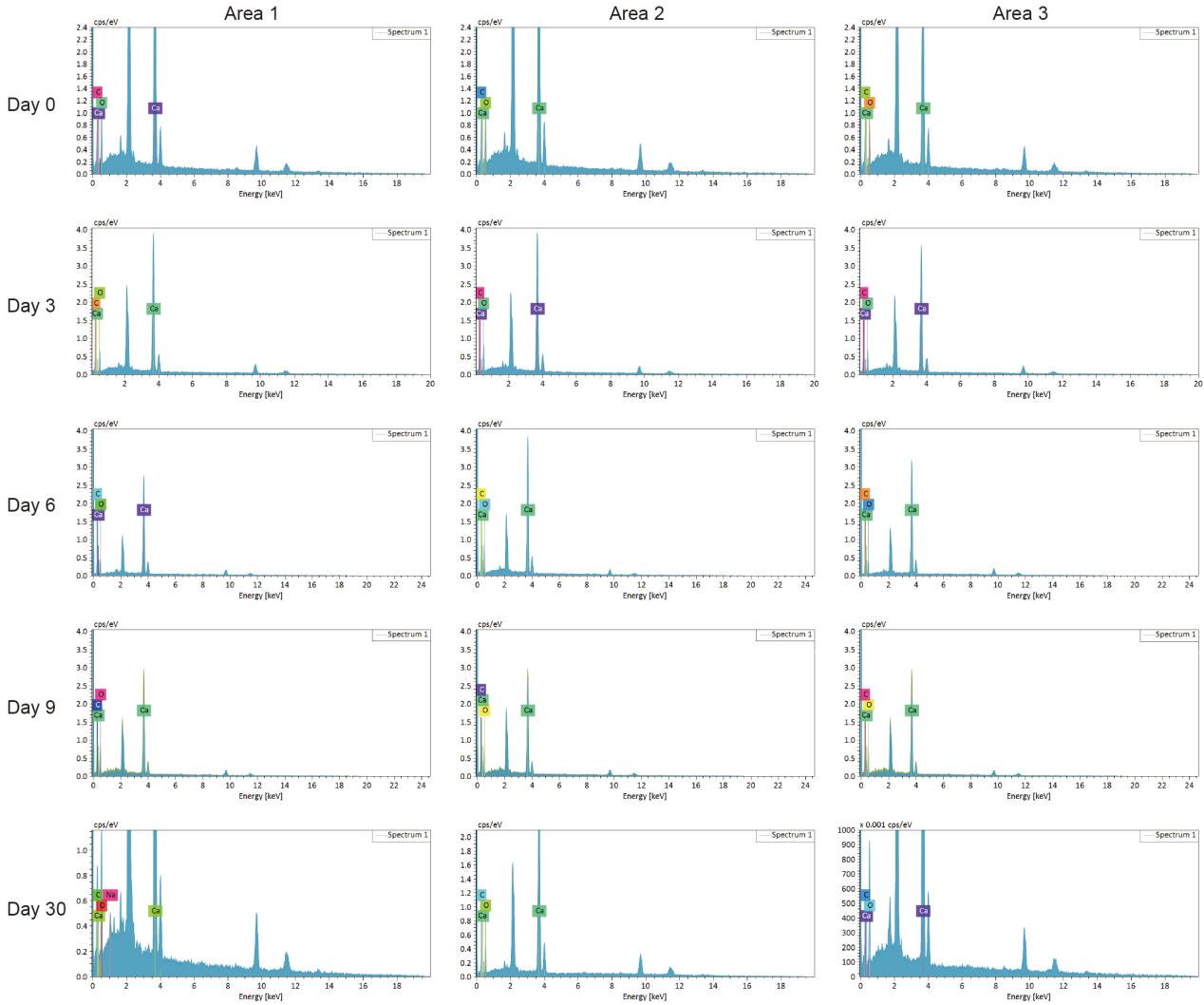


**Supplementary Figure 24 | EDS test of *M. capricornis*.** The atomic ratio of Ca decreased between Days 0 and 30, with rates of decrease of 1.01% in *M. capricornis*. The Ca atomic ratio remained constant between Days 0 and 3 in *M. capricornis*, and appeared to slightly rebound at Day 30 in *M. capricornis* from 24.24% to 25.74%.


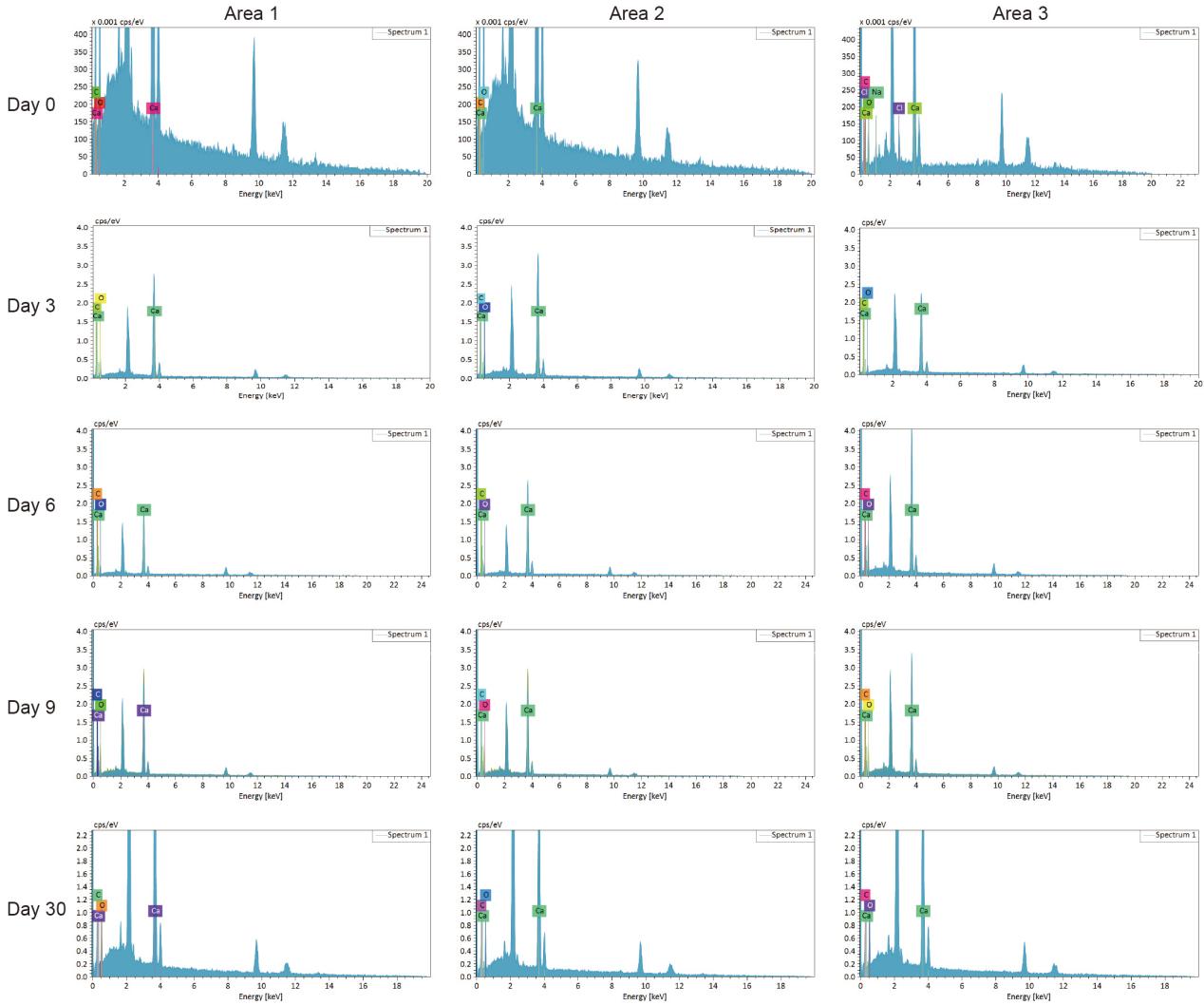


**Supplementary Figure 25 | EDS test of *M. foliosa*.** The atomic ratio of Ca decreased between Days 0 and 30, with rates of decrease of 2.26% in *M. foliosa*. The Ca atomic ratio remained constant between Days 0 and 3 in *M. foliosa*, and appeared to slightly rebound at Day 6 from 25.11% to 25.74%.


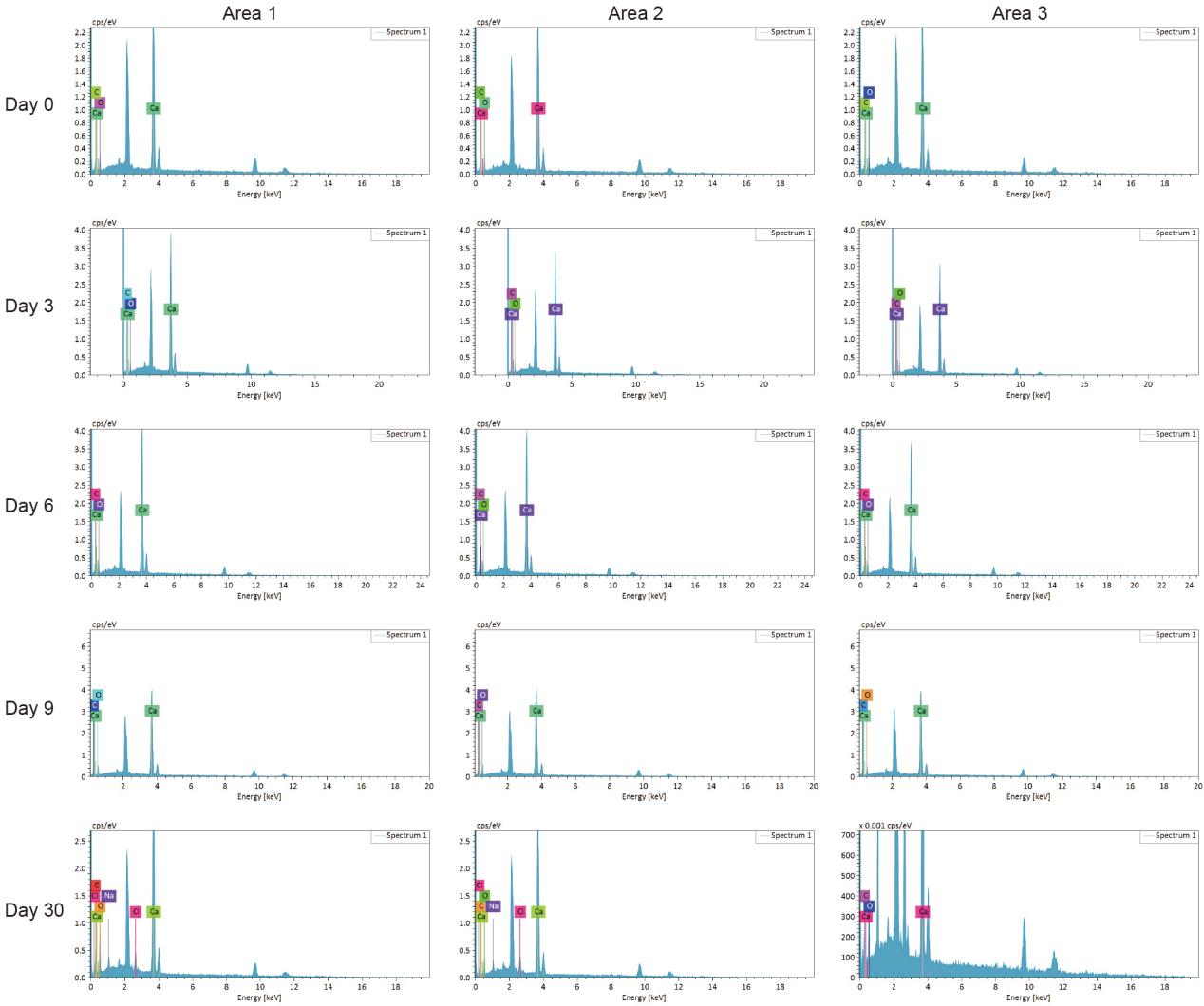


**Supplementary Figure 26 | EDS test of *P. damicornis*.** The atomic ratio of Ca decreased between Days 0 and 30, with rates of decrease of 5.94% in *P. damicornis*.


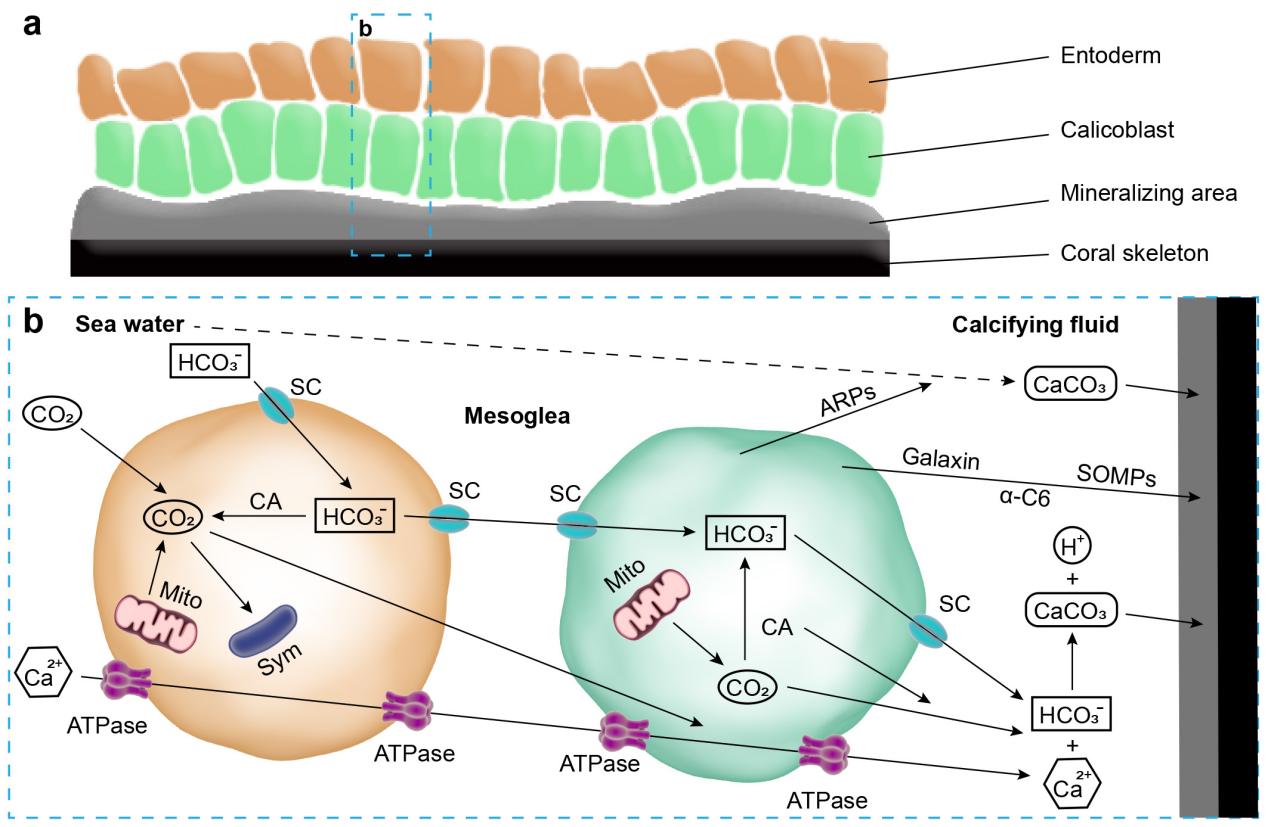


**Supplementary Figure 27 | Skeleton formation in reef-building corals.** a) Schematic diagram of coral skeletons and cells near the mineralizing area. b) ATPase means calcium ATPase; SC means solute carrier 4 and solute carrier 26; CA means carbonic anhydrase; Sym means Symbiodiniaceae; Mito means mitochondrion; ARPs means coral acid-rich proteins; α-C6 means collagen alpha-6(VI) chain-like; Galaxin means galaxin proteins; SOMPs means uncharacterized skeletal organic matrix proteins; the left cell belongs to entoderm, and the right cell belongs to calicoblast. The solid lines represent definite paths the dashed lines represent possible paths. Ca^2+^ transport by calcium ATPase or diffusion. CO_2_ can be converted into HCO^3−^ by CA and then exits the cells via bicarbonate transporters SC. ARPs can precipitate calcium carbonate from unamended seawater and modify the mineral polymorph. α-C6 and galaxin can cement the aragonite crystals to each other and to the underlying skeleton. The bioprecipitation of aragonite crystals in corals requires SOMPs.


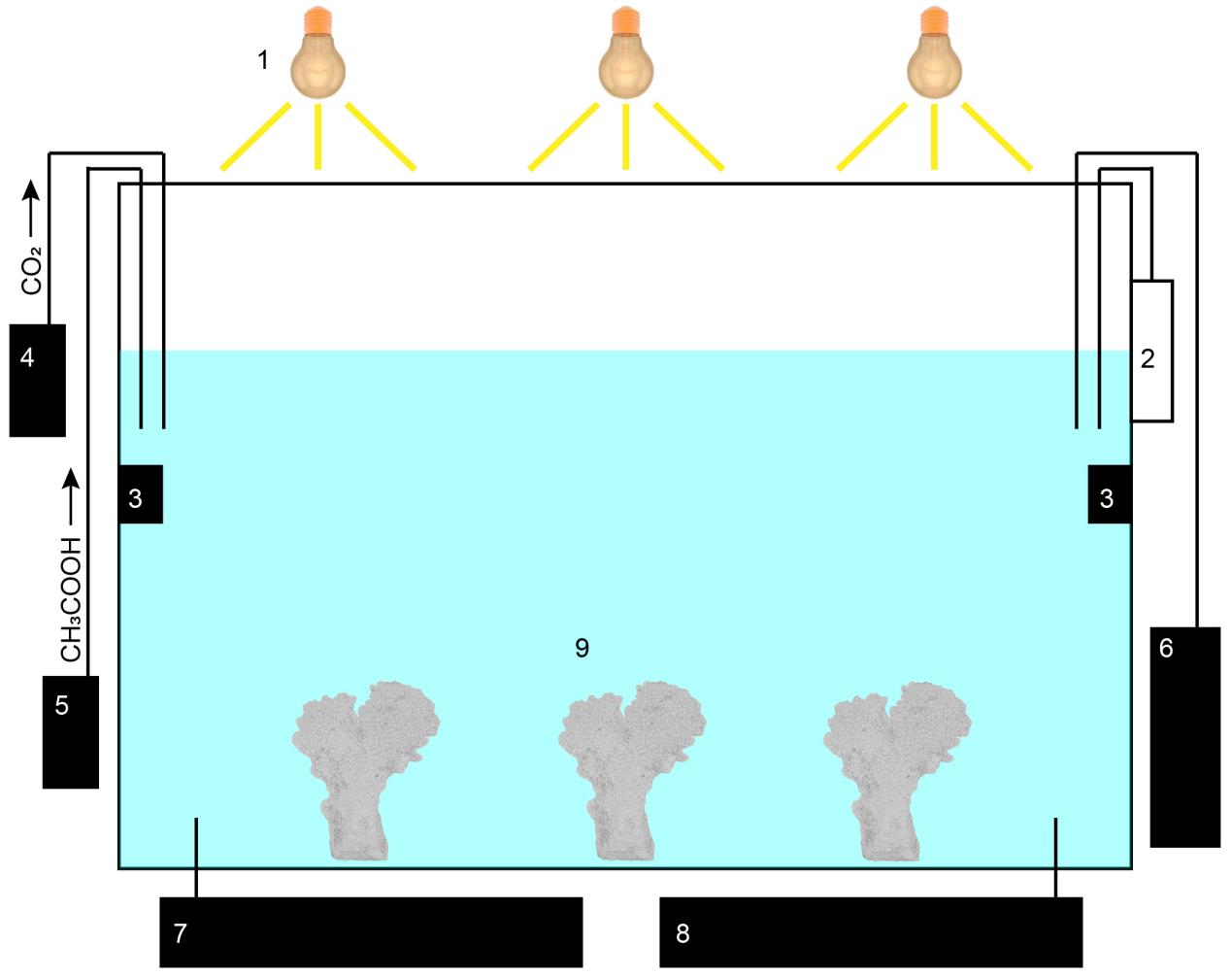


**Supplementary Figure 28 | Schematic diagram of the ocean acidification simulation device.** 1 means coral lamp, 2 means temperature and pH measuring instrument, 3 means wave device, 4 means CO_2_ bubbling device, 5 means microfluidic device for CH_3_COOH, 6 means calcium reactor, 7 means protein skimmer, 8 means water chiller, 9 means coral sample.


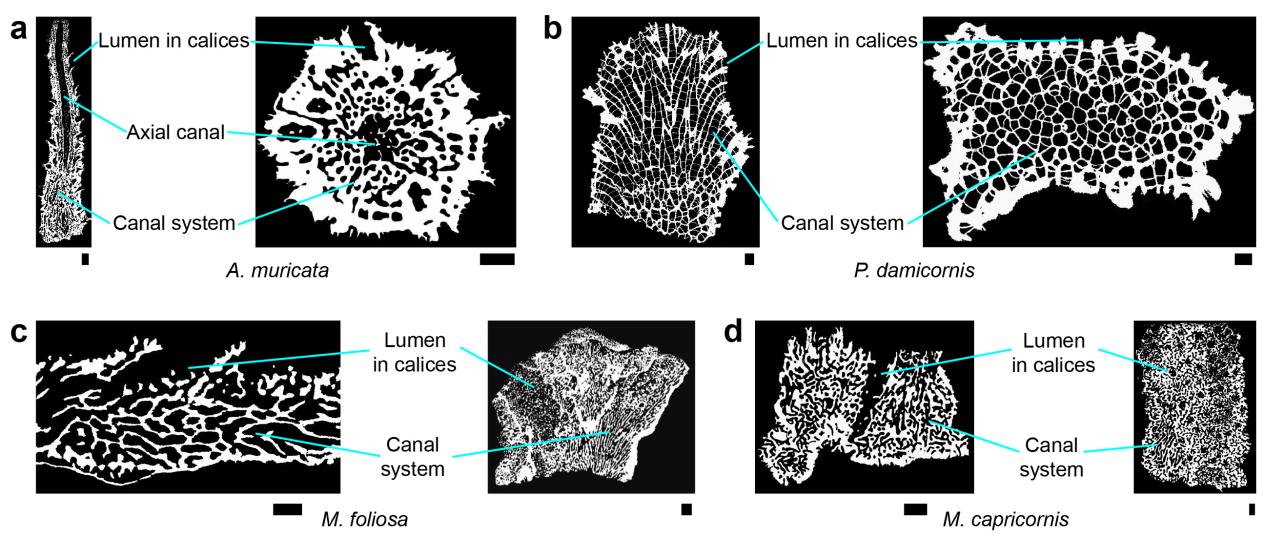


**Figure S29 | Distribution and differentiation of the canal system, axial canal, and lumen in calices within coral colonies.** The axial canal exists only in *Acropora* corals and is a large cylindrical canal located in the center of the coral branches, extending along the growth axis. Lumen in the calices refers to the oral cavity enclosed by the corallite within the calices. The remaining canals within the colony together form the canal system, connecting all calices in the colony for material and information exchange between polyps. Since the axial canal also has this function, in *Acropora* corals, the canal system includes the axial canal. Scale bars: 1mm.


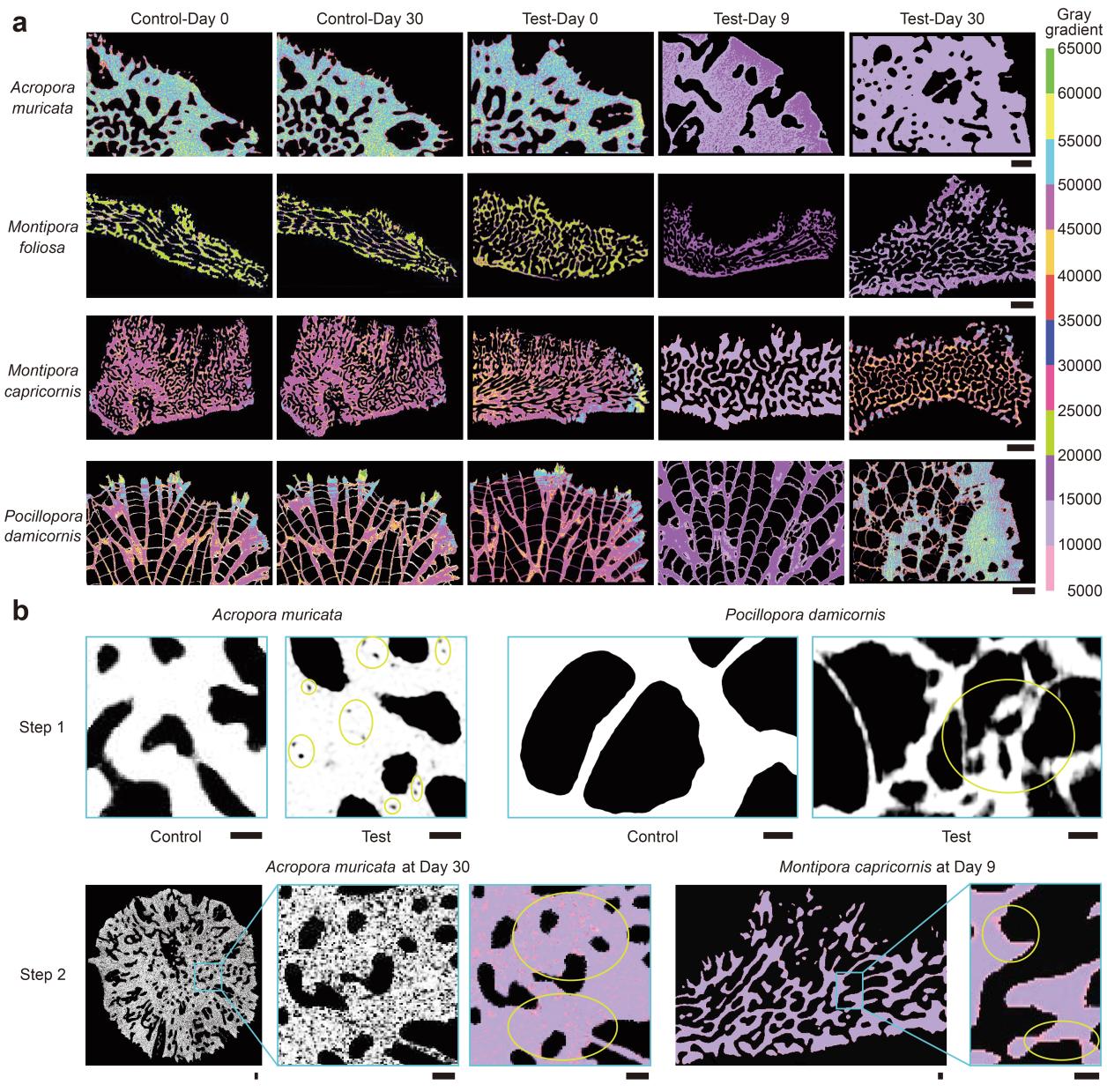


**Figure S30 | Grayscale heatmaps of four coral species and the identification method for acidic damage sites.** a) Branchlets from the same coral species and growth period typically displayed consistent grayscale distribution, while prolonged exposure to a low pH environment can alter this value. b) Step 1: Under normal conditions, the acidic damage sites within A. muricata skeletons, the damage to the inter-septal spaces and corallites of P. damicornis, as well as the newly constructed irregular skeleton inside calices, can be identified by comparing reconstructed images. Step 2: Since the grayscale of the skeleton ranges between 10,000 and 65,000, the canals and polyps have grayscale values approaching 0, and the acidic damage pores caused by reduces pH have grayscale values between 5,000 and 10,000, grayscale heatmaps can help us distinguish between the extensive acidic damage sites of A. muricata on Day 30 and the original channels. It can also assist in identifying acidic erosion areas in Montipora corals. These sites are difficult to distinguish directly from the reconstructed images compared to the original canal system. Scale bars: a) 1mm; b) 0.1 mm.

**Part 2 | Supplementary Tables**

**Supplementary Table 1 | Trends of skeleton to void ratio in coral colonies under acid stress.** Skeleton to void ratio is calculated as skeletal volume / total colony volume.

| **Species** | **Group** | **Day 0** | **Day 3** | **Day 6** | **Day 9** | **Day 30** |
| --- | --- | --- | --- | --- | --- | --- |
| ***Acropora***  ***muricata*** | G1 | 0.41 | 0.33 | 0.49 | 0.39 | 0.36 |
|  | G2 | 0.32 | 0.38 | 0.40 | 0.45 | 0.42 |
|  | G3 | 0.40 | 0.36 | 0.41 | 0.32 | 0.41 |
|  | G4 | 0.41 | 0.47 | 0.36 | 0.53 | 0.42 |
|  | G5 | 0.45 | 0.40 | 0.36 | 0.47 | 0.37 |
|  | G6 | 0.42 | 0.51 | 0.34 | 0.33 | 0.58 |
|  | G7 | 0.44 | 0.35 | 0.49 | 0.41 | 0.56 |
|  | G8 | 0.34 | 0.33 | 0.47 | 0.36 | 0.64 |
|  | G9 | 0.35 | 0.42 | 0.33 | 0.58 | 0.71 |
|  | Average | 0.394 | 0.395 | 0.406 | 0.428 | 0.497 |
| ***Montipora***  ***foliosa*** | G1 | 0.73 | 0.61 | 0.62 | 0.62 | 0.53 |
|  | G2 | 0.65 | 0.62 | 0.66 | 0.67 | 0.69 |
|  | G3 | 0.73 | 0.66 | 0.67 | 0.71 | 0.74 |
|  | G4 | 0.67 | 0.69 | 0.67 | 0.74 | 0.74 |
|  | G5 | 0.64 | 0.70 | 0.73 | 0.68 | 0.75 |
|  | G6 | 0.69 | 0.72 | 0.75 | 0.59 | 0.83 |
|  | G7 | 0.66 | 0.75 | 0.76 | 0.78 | 0.86 |
|  | G8 | 0.56 | 0.61 | 0.67 | 0.82 | 0.88 |
|  | G9 | 0.70 | 0.78 | 0.57 | 0.82 | 0.55 |
|  | Average | 0.670 | 0.682 | 0.678 | 0.714 | 0.730 |
| ***Montipora***  ***capricornis*** | G1 | 0.56 | 0.56 | 0.51 | 0.57 | 0.60 |
|  | G2 | 0.40 | 0.57 | 0.62 | 0.66 | 0.62 |
|  | G3 | 0.49 | 0.59 | 0.63 | 0.69 | 0.62 |
|  | G4 | 0.55 | 0.62 | 0.67 | 0.70 | 0.58 |
|  | G5 | 0.61 | 0.48 | 0.69 | 0.70 | 0.66 |
|  | G6 | 0.62 | 0.67 | 0.65 | 0.67 | 0.58 |
|  | G7 | 0.67 | 0.63 | 0.61 | 0.62 | 0.59 |
|  | G8 | 0.41 | 0.45 | 0.58 | 0.67 | 0.65 |
|  | G9 | 0.56 | 0.49 | 0.48 | 0.54 | 0.62 |
|  | Average | 0.541 | 0.562 | 0.604 | 0.647 | 0.613 |
| ***Pocillopora damicornis*** | G1 | 0.23 | 0.50 | 0.38 | 0.21 | 0.54 |
|  | G2 | 0.33 | 0.51 | 0.56 | 0.45 | 0.42 |
|  | G3 | 0.36 | 0.52 | 0.59 | 0.56 | 0.44 |
|  | G4 | 0.43 | 0.53 | 0.41 | 0.51 | 0.34 |
|  | G5 | 0.51 | 0.36 | 0.27 | 0.42 | 0.58 |
|  | G6 | 0.26 | 0.43 | 0.22 | 0.43 | 0.59 |
|  | G7 | 0.38 | 0.21 | 0.31 | 0.48 | 0.53 |
|  | G8 | 0.38 | 0.40 | 0.60 | 0.58 | 0.61 |
|  | G9 | 0.61 | 0.14 | 0.48 | 0.45 | 0.52 |
|  | Average | 0.388 | 0.400 | 0.424 | 0.454 | 0.508 |

**Supplementary Table 2 | Trends of element ratio in coral skeletons under acid stress.** A.e is an abbreviation for Abs.error [%] (3 sigma).

| **Species** | **Group** | **Element** | **Day 0** | **A.e** | **Day 3** | **A.e** | **Day 6** | **A.e** | **Day 9** | **A.e** | **Day 30** | **A.e** |
| --- | --- | --- | --- | --- | --- | --- | --- | --- | --- | --- | --- | --- |
| ***Acropora***  ***muricata*** | G1 | Ca | 25.70% | 0.75 | 35.58% | 0.73 | 25.89% | 0.96 | 23.85% | 0.99 | 18.12% | 0.99 |
|  |  | O | 53.23% | 2.51 | 47.63% | 4.30 | 59.00% | 4.65 | 60.11% | 6.18 | 56.85% | 5.90 |
|  |  | C | 21.07% | 1.26 | 16.79% | 1.64 | 15.12% | 1.20 | 16.04% | 1.68 | 24.57% | 1.68 |
|  | G2 | Ca | 25.83% | 0.69 | 20.06% | 0.71 | 25.94% | 0.98 | 24.57% | 0.98 | 24.87% | 0.98 |
|  |  | O | 52.47% | 3.14 | 55.19% | 3.79 | 58.77% | 5.50 | 60.04% | 5.43 | 56.04% | 8.26 |
|  |  | C | 21.70% | 1.30 | 24.75% | 1.36 | 15.29% | 1.53 | 15.40% | 1.35 | 19.09% | 3.16 |
|  | G3 | Ca | 30.67% | 0.71 | 25.72% | 0.72 | 26.96% | 0.83 | 24.95% | 0.94 | 23.01% | 0.91 |
|  |  | O | 44.65% | 4.09 | 47.41% | 3.92 | 55.81% | 5.50 | 59.51% | 5.23 | 58.19% | 4.35 |
|  |  | C | 24.68% | 1.54 | 26.88% | 1.37 | 17.23% | 1.68 | 15.54% | 1.54 | 18.81% | 1.32 |
|  | Average | Ca | 27.40% | - | 27.12% | - | 26.26% | - | 24.46% | - | 22.00% | - |
|  |  | O | 50.12% | - | 50.08% | - | 57.86% | - | 59.89% | - | 57.03% | - |
|  |  | C | 22.48% | - | 22.81% | - | 15.88% | - | 15.66% | - | 20.82% | - |
| ***Montipora***  ***foliosa*** | G1 | Ca | 24.28% | 0.93 | 26.57% | 0.90 | 25.00% | 0.79 | 25.22% | 0.90 | 21.94% | 0.87 |
|  |  | O | 58.01% | 5.69 | 56.07% | 4.48 | 55.89% | 3.99 | 52.85% | 6.37 | 56.32% | 4.19 |
|  |  | C | 17.71% | 1.72 | 17.36% | 1.14 | 19.11% | 1.61 | 21.93% | 2.09 | 20.16% | 1.14 |
|  | G2 | Ca | 25.09% | 0.89 | 23.95% | 0.88 | 21.81% | 0.93 | 22.34% | 0.83 | 23.20% | 0.93 |
|  |  | O | 56.81% | 4.06 | 57.12% | 4.10 | 60.08% | 4.51 | 56.23% | 4.48 | 58.36% | 4.54 |
|  |  | C | 18.10% | 1.39 | 18.93% | 1.13 | 18.11% | 1.08 | 21.43% | 1.28 | 18.44% | 1.20 |
|  | G3 | Ca | 25.97% | 0.82 | 24.80% | 0.85 | 30.42% | 0.97 | 24.36% | 0.93 | 23.42% | 0.84 |
|  |  | O | 55.31% | 4.41 | 54.78% | 4.38 | 55.49% | 4.74 | 55.43% | 6.18 | 59.04% | 4.77 |
|  |  | C | 18.71% | 1.35 | 20.42% | 1.24 | 14.10% | 1.28 | 20.21% | 1.79 | 17.54% | 1.53 |
|  | Average | Ca | 25.11% | - | 25.11% | - | 25.74% | - | 23.64% | - | 22.85% | - |
|  |  | O | 56.71% | - | 55.99% | - | 57.15% | - | 54.84% | - | 57.91% | - |
|  |  | C | 18.17% | - | 18.90% | - | 17.11% | - | 21.19% | - | 18.71% | - |
| ***Montipora***  ***capricomis*** | G1 | Ca | 28.88% | 0.89 | 23.94% | 0.97 | 25.90% | 0.88 | 21.45% | 0.87 | 27.24% | 0.72 |
|  |  | O | 52.80% | 4.19 | 54.69% | 3.96 | 49.68% | 4.55 | 62.16% | 4.46 | 58.02% | 4.26 |
|  |  | C | 18.32% | 1.32 | 21.37% | 1.34 | 24.42% | 1.35 | 16.38% | 1.40 | 14.74% | 1.58 |
|  | G2 | Ca | 26.47% | 0.98 | 23.00% | 0.83 | 30.16% | 0.86 | 21.59% | 0.72 | 25.61% | 0.87 |
|  |  | O | 51.56% | 3.93 | 57.64% | 5.11 | 55.01% | 4.42 | 61.44% | 3.92 | 57.37% | 5.42 |
|  |  | C | 21.98% | 1.42 | 19.37% | 1.67 | 14.83% | 1.37 | 16.96% | 1.37 | 17.02% | 1.94 |
|  | G3 | Ca | 24.89% | 0.90 | 33.32% | 0.93 | 19.38% | 0.84 | 29.68% | 0.73 | 24.37% | 0.79 |
|  |  | O | 49.56% | 4.49 | 48.67% | 5.02 | 57.01% | 3.80 | 49.97% | 3.03 | 58.17% | 4.07 |
|  |  | C | 25.55% | 1.62 | 18.01% | 1.44 | 23.61% | 1.50 | 20.35% | 1.42 | 17.46% | 1.41 |
|  | Average | Ca | 26.75% | - | 26.75% | - | 25.15% | - | 24.24% | - | 25.74% | - |
|  |  | O | 51.31% | - | 53.67% | - | 53.90% | - | 57.86% | - | 57.85% | - |
|  |  | C | 21.95% | - | 19.58% | - | 20.95% | - | 17.90% | - | 16.41% | - |
| ***Pocillopora damicornis*** | G1 | Ca | 38.45% | 0.73 | 36.99% | 0.96 | 34.42% | 0.96 | 30.85% | 0.83 | 26.56% | 0.94 |
|  |  | O | 46.64% | 3.37 | 45.65% | 4.30 | 52.65% | 4.65 | 54.93% | 5.50 | 42.23% | 5.23 |
|  |  | C | 14.91% | 1.43 | 17.35% | 1.45 | 12.94% | 1.20 | 14.22% | 1.68 | 22.61% | 1.54 |
|  | G2 | Ca | 36.39% | 0.76 | 37.43% | 0.97 | 33.47% | 0.96 | 31.53% | 0.99 | 29.59% | 0.99 |
|  |  | O | 49.34% | 4.67 | 45.67% | 4.83 | 51.30% | 4.65 | 54.66% | 6.18 | 43.26% | 5.90 |
|  |  | C | 14.27% | 1.67 | 16.90% | 1.61 | 15.23% | 1.20 | 13.81% | 1.68 | 22.13% | 1.68 |
|  | G3 | Ca | 34.54% | 0.84 | 30.48% | 0.79 | 33.57% | 0.98 | 32.74% | 0.98 | 29.41% | 0.93 |
|  |  | O | 47.16% | 3.53 | 53.25% | 4.43 | 50.63% | 5.50 | 53.32% | 5.43 | 55.50% | 4.89 |
|  |  | C | 18.30% | 1.37 | 16.27% | 1.45 | 15.79% | 1.53 | 13.94% | 1.35 | 15.09% | 1.33 |
|  | Average | Ca | 36.46% | - | 34.97% | - | 33.82% | - | 31.71% | - | 28.52% | - |
|  |  | O | 47.10% | - | 48.19% | - | 51.53% | - | 54.30% | - | 47.00% | - |
|  |  | C | 15.78% | - | 16.85% | - | 14.65% | - | 13.99% | - | 19.94% | - |

**Supplementary Table 3 | Gene expression changes of coral skeletome under acid stress.**

| **Species** | **Gene type** | **Protein** | **Day 0** | | | | **Day 3** | | | | **Day 9** | | | |
| --- | --- | --- | --- | --- | --- | --- | --- | --- | --- | --- | --- | --- | --- | --- |
|  |  |  | **G1** | **G2** | **G3** | **Ave.** | **G1** | **G2** | **G3** | **Ave.** | **G1** | **G2** | **G3** | **Ave.** |
| ***Pocillopora damicornis*** | Calcium-transporting ATPase | PMC-t ATPase 2 | 177 | 313 | 363 | 284 | 86 | 86 | 92 | 88 | 35 | 14 | 44 | 31 |
|  |  | PMC ATPase | 323 | 418 | 389 | 377 | 153 | 129 | 259 | 180 | 94 | 98 | 177 | 123 |
|  | Carbonic anhydrase | CA-1 | 173 | 226 | 187 | 195 | 393 | 372 | 483 | 416 | 300 | 437 | 351 | 363 |
|  |  | CA-2 | 90 | 145 | 143 | 126 | 141 | 163 | 188 | 164 | 81 | 62 | 54 | 66 |
|  |  | CA-3 | 5913 | 8226 | 7513 | 7217 | 10374 | 10210 | 12543 | 11042 | 4170 | 5358 | 4695 | 4741 |
|  | Acidic protein | SAARP-1 | 6382 | 8179 | 7618 | 7393 | 4877 | 5012 | 5890 | 5260 | 1588 | 2040 | 1788 | 1805 |
|  |  | SAARP-2 | 190 | 212 | 189 | 197 | 2485 | 2548 | 3088 | 2707 | 5459 | 6781 | 5660 | 5967 |
|  |  | ASOMP | 98 | 132 | 130 | 120 | 207 | 146 | 254 | 202 | 77 | 102 | 86 | 88 |
|  | skeletal organic matrix protein | USOMP-3 | 798 | 1045 | 1080 | 974 | 265 | 259 | 296 | 273 | 145 | 195 | 148 | 162 |
|  |  | USOMP-5 | 824 | 1190 | 973 | 996 | 173 | 163 | 234 | 190 | 0 | 2 | 1 | 1 |
|  |  | USOMP-7 | 302 | 418 | 389 | 370 | 357 | 355 | 446 | 386 | 66 | 105 | 86 | 86 |
|  | Binding Protein | Galaxin | 438 | 593 | 576 | 536 | 8390 | 8680 | 10324 | 9131 | 9123 | 11378 | 9598 | 10033 |
| ***Acropora muricata*** | Calcium-transporting ATPase | PMC-t ATPase 2 | 517 | 478 | 656 | 550 | 37 | 70 | 79 | 62 | 6 | 0 | 18 | 8 |
|  |  | PMC-t ATPase 3 | 141 | 156 | 150 | 149 | 86 | 78 | 88 | 84 | 56 | 83 | 39 | 60 |
|  |  | PMC ATPase | 0 | 0 | 0 | 0 | 0 | 0 | 0 | 0 | 29 | 24 | 52 | 35 |
|  | Carbonic anhydrase | CA-1 | 689 | 595 | 539 | 608 | 273 | 353 | 327 | 318 | 178 | 261 | 236 | 225 |
|  |  | CA-2 | 1517 | 1626 | 1693 | 1612 | 63 | 79 | 79 | 74 | 9 | 20 | 15 | 15 |
|  |  | CA-12 | 2402 | 2163 | 2350 | 2305 | 5009 | 5846 | 6940 | 5931 | 2610 | 4054 | 3246 | 3303 |
|  | Acidic protein | SAARP-1 | 1225 | 1080 | 1241 | 1182 | 867 | 956 | 1123 | 982 | 238 | 326 | 309 | 291 |
|  |  | SAARP-2 | 638 | 577 | 616 | 610 | 239 | 266 | 290 | 265 | 1111 | 1589 | 1260 | 1320 |
|  |  | ASOMP | 7022 | 6296 | 6864 | 6727 | 1330 | 1486 | 1715 | 1510 | 1314 | 1796 | 1460 | 1523 |
|  |  | SAP-1 | 1308 | 1146 | 1380 | 1278 | 151 | 191 | 213 | 185 | 93 | 160 | 101 | 118 |
|  |  | SAP-2 | 6864 | 6450 | 7216 | 6843 | 4405 | 5098 | 5966 | 5156 | 11973 | 18929 | 14937 | 15280 |
|  | skeletal organic matrix protein | USOMP-1 | 199 | 232 | 245 | 225 | 0 | 0 | 0 | 0 | 0 | 0 | 0 | 0 |
|  |  | USOMP-2 | 494 | 434 | 429 | 453 | 42 | 42 | 65 | 50 | 7 | 11 | 1 | 6 |
|  |  | USOMP-3 | 22 | 56 | 29 | 36 | 0 | 0 | 0 | 0 | 0 | 0 | 0 | 0 |
|  |  | USOMP-4 | 4328 | 3847 | 4033 | 4069 | 3027 | 3939 | 4574 | 3847 | 755 | 1123 | 878 | 919 |
|  |  | USOMP-5 | 284 | 254 | 285 | 274 | 54 | 70 | 74 | 66 | 6 | 38 | 20 | 21 |
|  |  | USOMP-6 | 77742 | 69677 | 78447 | 75288 | 100322 | 119931 | 142703 | 120985 | 41548 | 62678 | 49748 | 51324 |
|  |  | USOMP-7 | 1251 | 1064 | 1179 | 1165 | 689 | 886 | 1022 | 866 | 560 | 967 | 726 | 751 |
|  |  | USOMP-8 | 367 | 325 | 322 | 338 | 296 | 323 | 318 | 312 | 134 | 257 | 192 | 194 |
|  | Binding Protein | α-C6 | 279 | 238 | 310 | 276 | 408 | 391 | 649 | 483 | 357 | 698 | 550 | 535 |
|  |  | Galaxin | 1981 | 1737 | 1924 | 1881 | 2803 | 3330 | 3614 | 3249 | 64382 | 98403 | 76690 | 79825 |
| ***Montipora capricornis*** | Calcium-transporting ATPase | PMC-t ATPase 2 | 1517 | 1699 | 1795 | 1670 | 1006 | 1242 | 1157 | 1135 | 1210 | 1179 | 785 | 1058 |
|  |  | PMC-t ATPase 3 | 67 | 50 | 88 | 69 | 10 | 0 | 28 | 13 | 13 | 13 | 16 | 14 |
|  |  | PMC-t ATPase 4 | 131 | 119 | 140 | 130 | 45 | 79 | 64 | 63 | 61 | 65 | 79 | 68 |
|  |  | PMC ATPase | 1367 | 1398 | 1710 | 1492 | 305 | 452 | 473 | 410 | 610 | 594 | 429 | 544 |
|  | Solute carrier | SC-4 | 426 | 490 | 573 | 497 | 353 | 438 | 499 | 430 | 550 | 340 | 438 | 443 |
|  |  | SC-26 | 3279 | 3545 | 3582 | 3469 | 818 | 944 | 849 | 870 | 2747 | 2649 | 1985 | 2460 |
|  | Carbonic anhydrase | CA-1 | 200 | 228 | 215 | 214 | 293 | 294 | 285 | 291 | 396 | 328 | 275 | 333 |
|  |  | CA-2 | 1408 | 1561 | 1440 | 1470 | 333 | 469 | 395 | 399 | 1352 | 1348 | 1014 | 1238 |
|  |  | CA-12 | 2465 | 2753 | 2906 | 2708 | 1368 | 1599 | 1465 | 1477 | 3201 | 2758 | 1863 | 2607 |
|  | Acidic protein | SAARP-1 | 981 | 1006 | 991 | 993 | 114 | 191 | 176 | 160 | 948 | 812 | 597 | 785 |
|  |  | SAARP-2 | 500 | 498 | 518 | 505 | 949 | 1144 | 1017 | 1037 | 13342 | 12549 | 9634 | 11842 |
|  |  | ASOMP | 1857 | 1920 | 1888 | 1889 | 139 | 204 | 186 | 176 | 2216 | 1880 | 1478 | 1858 |
|  |  | SAP-1 | 1207 | 1357 | 1303 | 1289 | 572 | 636 | 624 | 611 | 836 | 764 | 538 | 712 |
|  |  | SAP-2 | 1987 | 2188 | 2118 | 2097 | 2260 | 2674 | 2410 | 2448 | 5758 | 5668 | 4267 | 5231 |
|  |  | AGARP | 3373 | 3601 | 3576 | 3517 | 3239 | 3530 | 3282 | 3350 | 2669 | 2585 | 1987 | 2414 |
|  | skeletal organic matrix protein | USOMP-2 | 3207 | 3492 | 3310 | 3337 | 196 | 256 | 172 | 208 | 995 | 797 | 660 | 817 |
|  |  | USOMP-3 | 1912 | 1882 | 1947 | 1914 | 243 | 242 | 296 | 260 | 575 | 518 | 423 | 505 |
|  |  | USOMP-5 | 122 | 126 | 128 | 125 | 230 | 297 | 242 | 256 | 1317 | 1311 | 964 | 1197 |
|  |  | USOMP-6 | 13676 | 15792 | 16076 | 15181 | 21927 | 25530 | 22440 | 23299 | 25793 | 25900 | 18981 | 23558 |
|  |  | USOMP-7 | 1238 | 1319 | 1310 | 1289 | 669 | 771 | 712 | 717 | 1647 | 1516 | 1118 | 1427 |
|  |  | USOMP-8 | 32 | 56 | 52 | 47 | 72 | 83 | 51 | 69 | 24 | 25 | 23 | 24 |
|  | Binding Protein | α-C6 | 437 | 433 | 430 | 433 | 37 | 43 | 69 | 50 | 277 | 241 | 203 | 240 |
|  |  | Galaxin | 452 | 433 | 435 | 440 | 84 | 104 | 83 | 90 | 42 | 51 | 29 | 41 |
| ***Montipora foliosa*** | Calcium-transporting ATPase | PMC-t ATPase 2 | 2025 | 1980 | 1827 | 1944 | 2024 | 1427 | 1381 | 1611 | 3156 | 2402 | 3655 | 3071 |
|  |  | PMC-t ATPase 3 | 3 | 17 | 13 | 11 | 2 | 3 | 4 | 3 | 24 | 49 | 56 | 43 |
|  |  | PMC-t ATPase 4 | 455 | 431 | 455 | 447 | 181 | 110 | 104 | 132 | 167 | 134 | 192 | 164 |
|  |  | PMC ATPase | 2726 | 2598 | 2652 | 2659 | 1939 | 1496 | 1315 | 1583 | 2877 | 2057 | 3052 | 2662 |
|  | Solute carrier | SC-4 | 409 | 415 | 369 | 398 | 459 | 321 | 332 | 371 | 2486 | 1849 | 2872 | 2402 |
|  |  | SC-26 | 500 | 468 | 398 | 455 | 731 | 536 | 508 | 592 | 988 | 713 | 1193 | 965 |
|  | Carbonic anhydrase | CA-1 | 838 | 836 | 748 | 807 | 568 | 414 | 359 | 447 | 638 | 484 | 769 | 630 |
|  |  | CA-2 | 24608 | 23301 | 21649 | 23186 | 1085 | 829 | 775 | 896 | 2061 | 1469 | 2266 | 1932 |
|  |  | CA-12 | 1673 | 1727 | 1606 | 1668 | 1323 | 961 | 977 | 1087 | 773 | 560 | 882 | 738 |
|  | Acidic protein | SAARP-1 | 732 | 706 | 713 | 717 | 1065 | 702 | 765 | 844 | 1807 | 1434 | 2018 | 1753 |
|  |  | SAARP-2 | 161 | 150 | 169 | 160 | 1276 | 807 | 811 | 965 | 829 | 676 | 937 | 814 |
|  |  | ASOMP | 258 | 283 | 296 | 279 | 85 | 42 | 50 | 59 | 60 | 48 | 95 | 68 |
|  |  | SAP-1 | 1923 | 1704 | 1739 | 1789 | 986 | 703 | 628 | 772 | 1339 | 845 | 1423 | 1202 |
|  |  | SAP-2 | 9974 | 9564 | 9037 | 9525 | 10963 | 7894 | 7184 | 8680 | 5537 | 3371 | 5406 | 4771 |
|  |  | AGARP | 2630 | 2408 | 2217 | 2418 | 4522 | 3230 | 3042 | 3598 | 3161 | 2598 | 3789 | 3183 |
|  | skeletal organic matrix protein | USOMP-2 | 477 | 495 | 473 | 482 | 25 | 15 | 23 | 21 | 115 | 95 | 113 | 108 |
|  |  | USOMP-3 | 3138 | 3058 | 2849 | 3015 | 637 | 429 | 435 | 500 | 183 | 108 | 179 | 157 |
|  |  | USOMP-5 | 265 | 280 | 225 | 257 | 750 | 529 | 500 | 593 | 735 | 582 | 848 | 722 |
|  |  | USOMP-6 | 48207 | 46137 | 39596 | 44647 | 68739 | 48864 | 46750 | 54784 | 73926 | 60864 | 92171 | 75654 |
|  |  | USOMP-7 | 28 | 22 | 27 | 26 | 76 | 37 | 40 | 51 | 112 | 73 | 123 | 103 |
|  |  | USOMP-8 | 22 | 38 | 29 | 30 | 101 | 60 | 64 | 75 | 76 | 66 | 107 | 83 |
|  | Binding Protein | α-C6 | 396 | 459 | 415 | 423 | 768 | 510 | 486 | 588 | 780 | 605 | 895 | 760 |
|  |  | Galaxin | 5171 | 5105 | 4725 | 5000 | 4999 | 3634 | 3430 | 4021 | 1571 | 1222 | 1698 | 1497 |

**Supplementary Table 4 | Parameters of the micro-CT tests.**

| **Samples** | **Voltage** | **Current** | **Voxel size** | **Timing** | **Number of images** | **Image width** | **Image height** |
| --- | --- | --- | --- | --- | --- | --- | --- |
| **Day 0 *Acropora muricata*** | 120 kV | 90 μA | 9 μm | 500 ms | 4,000 | 2,300 pixels | 4,000 pixels |
| **Day 3 *Acropora muricata*** | 160 kV | 80 μA | 7 μm | 334 ms | 6,000 | 3,000 pixels | 4,000 pixels |
| **Day 6 *Acropora muricata*** | 110 kV | 80 μA | 4 μm | 1 s | 2,000 | 3,990 pixels | 4,000 pixels |
| **Day 9 *Acropora muricata*** | 130 kV | 70 μA | 3 μm | 1 s | 2,000 | 3,000 pixels | 3,800 pixels |
| **Day 30 *Acropora muricata*** | 150 kV | 70 μA | 8 μm | 500 ms | 4,000 | 2,000 pixels | 4,000 pixels |
| **Day 0 *Montipora foliosa*** | 200 kV | 100 μA | 18 μm | 500 ms | 4,000 | 2,024 pixels | 2,024 pixels |
| **Day 3 *Montipora foliosa*** | 180 kV | 90 μA | 14 μm | 500 ms | 2,500 | 4,000 pixels | 4,000 pixels |
| **Day 6 *Montipora foliosa*** | 200 kV | 90 μA | 11 μm | 500 ms | 4,000 | 3,500 pixels | 4,000 pixels |
| **Day 9 *Montipora foliosa*** | 180 kV | 80 μA | 9 μm | 500 ms | 4,000 | 3,990 pixels | 4,000 pixels |
| **Day 30 *Montipora foliosa*** | 180 kV | 80 μA | 10 μm | 500 ms | 4,000 | 3,990 pixels | 4,000 pixels |
| **Day 0 *Montipora capricomis*** | 120 kV | 90 μA | 9 μm | 500 ms | 4,000 | 3,300 pixels | 2,500 pixels |
| **Day 3 *Montipora capricomis*** | 180 kV | 90 μA | 8 μm | 500 ms | 4,000 | 3,990 pixels | 4,000 pixels |
| **Day 6 *Montipora capricomis*** | 180 kV | 80 μA | 11 μm | 500 ms | 4,000 | 3,990 pixels | 4,000 pixels |
| **Day 9 *Montipora capricomis*** | 180 kV | 80 μA | 8 μm | 500 ms | 4,000 | 3,990 pixels | 4,000 pixels |
| **Day 30 *Montipora capricomis*** | 180 kV | 90 μA | 9 μm | 500 ms | 4,000 | 3,990 pixels | 4,000 pixels |
| **Day 0 *Pocillopora damicornis*** | 220 kV | 120 μA | 7 μm | 1 s | 2,000 | 3,990 pixels | 4,000 pixels |
| **Day 3 *Pocillopora damicornis*** | 200 kV | 90 μA | 14 μm | 500 ms | 4,000 | 2,800 pixels | 4,000 pixels |
| **Day 6 *Pocillopora damicornis*** | 190 kV | 80 μA | 9 μm | 500 ms | 4,000 | 3,400 pixels | 4,000 pixels |
| **Day 9 *Pocillopora damicornis*** | 210 kV | 90 μA | 14 μm | 500 ms | 4,000 | 3,990 pixels | 4,000 pixels |
| **Day 30 *Pocillopora damicornis*** | 190 kV | 80 μA | 13 μm | 500 ms | 4,000 | 3,990 pixels | 4,000 pixels |

**Supplementary Table 5 | Carbonate chemistry in coral tank during the process of ocean acidification simulation (14 days), while pCO_2_ rose from 380 μatm at Day 0 to 800 μatm at Pre-14.**

|  | Day0 | Pre-1 | Pre-2 | Pre-3 | Pre-4 | Pre-5 | Pre-6 | Pre-7 | Pre-8 | Pre-9 | Pre-10 | Pre-11 | Pre-12 | Pre-13 | Pre-14 |
| --- | --- | --- | --- | --- | --- | --- | --- | --- | --- | --- | --- | --- | --- | --- | --- |
| pH | 8.20 | 8.10 | 8.04 | 8.01 | 7.98 | 7.95 | 7.93 | 7.90 | 7.88 | 7.86 | 7.85 | 7.84 | 7.82 | 7.81 | 7.80 |
| Ca^2+^ | 399 | 396 | 400 | 399 | 401 | 400 | 400 | 402 | 402 | 402 | 402 | 402 | 404 | 405 | 410 |
| CO_3_^2-^ | 20.0 | 16.5 | 14.6 | 13.7 | 12.9 | 12.1 | 11.6 | 10.8 | 10.4 | 9.9 | 9.7 | 9.5 | 9.1 | 8.9 | 8.7 |
| HCO_3_^-^ | 112.5 | 116.5 | 118.4 | 119.3 | 120.0 | 120.7 | 121.1 | 121.6 | 122.0 | 122.2 | 122.4 | 122.5 | 122.7 | 122.8 | 122.9 |

**Part 3 | Data Availability Statement**

The datasets (reef-building coral holobionts full-length and short-read transcriptome sequencing raw data) generated during the current study are available at the Sequence Read Archive (SRA) publicly available repository, [https://www.ncbi.nlm.nih.gov/sra/].

Three generations of full-length transcriptome raw data: SAMN16237127 : Coral_OA1_day0 RNA-Seq of Pocillopora damicornis2: polyps; SAMN16237128 : Coral_OA2_day0 RNA-Seq of Acropora muricata: polyps; SAMN16237129 : Coral_OA3_day0 RNA-Seq of Montipora capricornis: polyps; SAMN16237130 : Coral_OA4_day0 RNA-Seq of Montipora foliosa: polyps.

Three generations of full-length transcriptome annotation data: SAMN16456055 : Coral_OA1_day0_Gene_Expression RNA-Seq of Pocillopora damicornis2: polyps; SAMN16456056 : Coral_OA2_day0_Gene_Expression RNA-Seq of Acropora muricata: polyps; SAMN16456057 : Coral_OA3_day0_Gene_Expression RNA-Seq of Montipora capricornis: polyps; SAMN16456058 : Coral_OA4_day0_Gene_Expression RNA-Seq of Montipora foliosa: polyps.

Second-generation transcriptome raw data : SAMN16365802：Coral_OA_1_day0_1 Pocillopora damicornis_2; SAMN16365803：Coral_OA_1_day0_2 Pocillopora damicornis_2; SAMN16365804：Coral_OA_1_day0_3 Pocillopora damicornis_2; SAMN16365805：Coral_OA_2_day0_1 Acropora muricata: polyps; SAMN16365806：Coral_OA_2_day0_2 Acropora muricata: polyps; SAMN16365807：Coral_OA_2_day0_3 Acropora muricata: polyps; SAMN16365808：Coral_OA_3_day0_1 Montipora capricornis: polyps; SAMN16365809：Coral_OA_3_day0_2 Montipora capricornis: polyps; SAMN16365810：Coral_OA_3_day0_3 Montipora capricornis: polyps; SAMN16365811：Coral_OA_4_day0_1 Montipora foliosa: polyps; SAMN16365812：Coral_OA_4_day0_2 Montipora foliosa: polyps; SAMN16365813：Coral_OA_4_day0_3 Montipora foliosa: polyps; SAMN16237439：Coral_OA_1_day3_1 RNA-Seq of Pocillopora damicornis2: polyps; SAMN16237440：Coral_OA_1_day3_2 RNA-Seq of Pocillopora damicornis2: polyps; SAMN16237441：Coral_OA_1_day3_3 RNA-Seq of Pocillopora damicornis2: polyps; SAMN16237442：Coral_OA_2_day3_1 RNA-Seq of Acropora muricata: polyps; SAMN16237443：Coral_OA_2_day3_2 RNA-Seq of Acropora muricata: polyps; SAMN16237444：Coral_OA_2_day3_3 RNA-Seq of Acropora muricata: polyps; SAMN16237445：Coral_OA_3_day3_1 RNA-Seq of Montipora capricornis: polyps; SAMN16237446：Coral_OA_3_day3_2 RNA-Seq of Montipora capricornis: polyps; SAMN16237447：Coral_OA_3_day3_3 RNA-Seq of Montipora capricornis: polyps; SAMN16237448：Coral_OA_4_day3_1 RNA-Seq of Montipora foliosa: polyps; SAMN16237449：Coral_OA_4_day3_2 RNA-Seq of Montipora foliosa: polyps; SAMN16237450：Coral_OA_4_day3_3 RNA-Seq of Montipora foliosa: polyps; SAMN16237451：Coral_OA_1_day9_1 RNA-Seq of Pocillopora damicornis2: polyps; SAMN16237452：Coral_OA_1_day9_2 RNA-Seq of Pocillopora damicornis2: polyps; SAMN16237453：Coral_OA_1_day9_3 RNA-Seq of Pocillopora damicornis2: polyps; SAMN16237454：Coral_OA_2_day9_1 RNA-Seq of Acropora muricata: polyps; SAMN16237455：Coral_OA_2_day9_2 RNA-Seq of Acropora muricata: polyps; SAMN16237456：Coral_OA_2_day9_3 RNA-Seq of Acropora muricata: polyps; SAMN16237457：Coral_OA_3_day9_1 RNA-Seq of Montipora capricornis: polyps; SAMN16237458：Coral_OA_3_day9_2 RNA-Seq of Montipora capricornis: polyps; SAMN16237459：Coral_OA_3_day9_3 RNA-Seq of Montipora capricornis: polyps; SAMN16237460：Coral_OA_4_day9_1 RNA-Seq of Montipora foliosa: polyps; SAMN16237461：Coral_OA_4_day9_2 RNA-Seq of Montipora foliosa: polyps; SAMN16237462：Coral_OA_4_day9_3 RNA-Seq of Montipora foliosa: polyps.
